# Targeting the Membrane‐Embedded Rhomboid Protease GlpG: A Multimodal Strategy for Inhibitor Discovery and Mechanistic Insight

**DOI:** 10.1002/anie.202514067

**Published:** 2026-01-28

**Authors:** Claudia Bohg, Yurii Dubanych, Spyridon Kosteletos, Taoran Xiao, Martin Neuenschwander, Tillmann Utesch, Michael Lisurek, Carl Öster, Andreas Oder, Carola Seyffarth, Kathrin Bach, Denise‐Liù Gracias Leone, František Filandr, Marc Wegert, Sascha Lange, Henry Sawczyc, Jens Peter von Kries, Christian P. R. Hackenberger, Edgar Specker, Han Sun, Kvido Stříšovský, Adam Lange

**Affiliations:** ^1^ Research Unit Molecular Biophysics Leibniz Forschungsinstitut für Molekulare Pharmakologie (FMP) Berlin Germany; ^2^ Institute of Organic Chemistry and Biochemistry Czech Academy of Sciences Prague Czech Republic; ^3^ Faculty of Food and Biochemical Technology University of Chemistry and Technology Prague Czech Republic; ^4^ Core Facility Screening Unit Leibniz Forschungsinstitut für Molekulare Pharmakologie (FMP) Berlin Germany; ^5^ Research Unit Structural Chemistry & Computational Biophysics Leibniz Forschungsinstitut für Molekulare Pharmakologie (FMP) Berlin Germany; ^6^ Research Unit Biomolecule Modification and Delivery Leibniz Forschungsinstitut für Molekulare Pharmakologie (FMP) Berlin Germany; ^7^ Institute of Chemistry Humboldt‐Universität zu Berlin Berlin Germany; ^8^ Core Facility Compound Management Leibniz Forschungsinstitut für Molekulare Pharmakologie (FMP) Berlin Germany; ^9^ Institute of Chemistry Technische Universität Berlin Berlin Germany; ^10^ Institute of Biology Humboldt‐Universität zu Berlin Berlin Germany

**Keywords:** drug discovery, high‐throughput screening, intramembrane proteolysis, membrane enzymes, protein drug interactions

## Abstract

Rhomboid proteases, a class of intramembrane proteases characterized by a Ser‐His catalytic dyad, have recently emerged as promising therapeutic targets. While inhibitors for soluble serine proteases have been extensively studied, the spectrum of potent rhomboid protease inhibitor chemotypes is limited to active‐site targeted nucleophiles. To address this limitation, we conducted a high‐throughput screen of over 68,000 compounds targeting the *E. coli* rhomboid protease GlpG, using a fluorescent liposome‐based assay. A selection of 326 inhibitory compounds was evaluated in a subsequent IC_50_ screen against two variants of GlpG (core domain and full length), a soluble serine protease (chymotrypsin), as well as the human mitochondrial rhomboid PARL. Of these, the selective inhibitory effects of 2 compounds and their analogues on GlpG were confirmed through further biochemical and biophysical characterisation, molecular docking, and solid‐state NMR spectroscopy. This study paves the way for developing small‐molecule tool compounds and drug‐like molecules targeting rhomboid proteases.

## Graphical abstract

Created in BioRender. Bohg, C. (2026) https://BioRender.Com/ vi9hi4f.

Rhomboid proteases are a mechanistically unique and evolutionarily conserved protein family. Despite their pharmacological relevance, the development of selective inhibitors has lagged behind that of soluble proteases. Using a high‐throughput screen, novel chemotypes with selective inhibitory activity were identified. Biochemical assays, solid‐state NMR, and molecular docking were used to define inhibitor binding sites and modes.

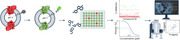

## 1 Introduction

Rhomboid proteases are intramembrane serine proteases, initially discovered in *Drosophila* and later identified across all domains of life [1, 2, 3]. They represent a highly conserved and widely distributed protein family with diverse biological functions ranging from protein quality control to regulation of cellular signalling pathways. In prokaryotes, GlpG contributes to maintaining membrane protein homeostasis, by eliminating orphan membrane proteins as shown in the Gram‐negative *Shigella sonnei* [4] and in uropathogenic *Escherichia coli* (*E. coli*) where it is essential for the assembly of type I pili by regulating FimA protein quality control and turnover [5]. GlpG has emerged as the most extensively studied rhomboid protease in terms of structural and biophysical investigations [6, 7, 8, 9, 10, 11]. In Gram positives, YqgP in *Bacillus subtilis* mediates the sensing and degradation of mis‐metallated magnesium transporters [12]. In mammals, the rhomboid protease family encompasses fourteen members, including five proteases, RHBDL1‐4 and PARL, and nine pseudoproteases [13, 14]. Mammalian rhomboid proteases are involved in a variety of biological processes: RHBDL2 is implicated in tissue homeostasis [15, 16, 17], RHBDL4 is involved in ER‐associated degradation pathways [18, 19], and PARL is linked to mitochondrial quality control and stress response by cleaving the mitophagy regulators PINK1 and PGAM5 [20, 21, 22, 23, 24, 25].

In accordance with their varied molecular functions and wide evolutionary occurrence, rhomboid proteases play critical roles in several disease contexts. More recently, GlpG has been identified to be critical for type I pili formation in uropathogenic *E. coli* (UPEC), directly impacting bacterial colonisation, host cell infection, and biofilm formation [5]. In malaria, caused by the protozoan parasite *Plasmodium falciparum*, rhomboid proteases are essential for parasite survival, as demonstrated using a scalable inducible knockout system [26], and inhibition of PfROM4 effectively clears the parasite in culture [27]. Similarly, a rhomboid protease is required for the pathogenicity of *Aspergillus fumigatus*, a causative agent of aspergillosis [28]. In humans, the mitochondrial rhomboid protease PARL regulates PINK1‐dependent mitophagy and ferroptosis [29], and ketoamide inhibitors of PARL stabilize PINK1 at the mitochondrial surface [30], which is known to promote mitophagy [23]. Independently, PARL dysfunction has been linked to Parkinson's disease, neurodegeneration and insulin resistance [31, 32, 33]. Additionally, RHBDL2 and RHBDL4 have been associated with cancer progression [17, 34], and RHBDL4 with Alzheimer's disease [17, 34, 35], and inflammatory signalling [36]. Collectively, these examples highlight the broad physiological and pathological relevance of rhomboid proteases, supporting their potential as therapeutic targets where small‐molecule inhibitors or modulators could correct dysregulated proteolytic activity or limit bacterial virulence and thereby address increasing antibiotic resistance.

Despite substantial efforts to develop inhibitors for rhomboid proteases since their discovery, progress has been slow. This may have been due to limited three‐dimensional structural information, the unique composition of the active site — a catalytic dyad instead of the classical triad — or the added complexity of their membrane‐embedded nature. Despite these challenges, rhomboid protease inhibitors have been identified, including isocoumarins [37], β‐lactams [38, 39], saccharins [40], benzoxazinones [41, 42], succinimides [43], and peptide‐based covalent inhibitors [11, 27, 30, 44, 45, 46].

**FIGURE 1:**
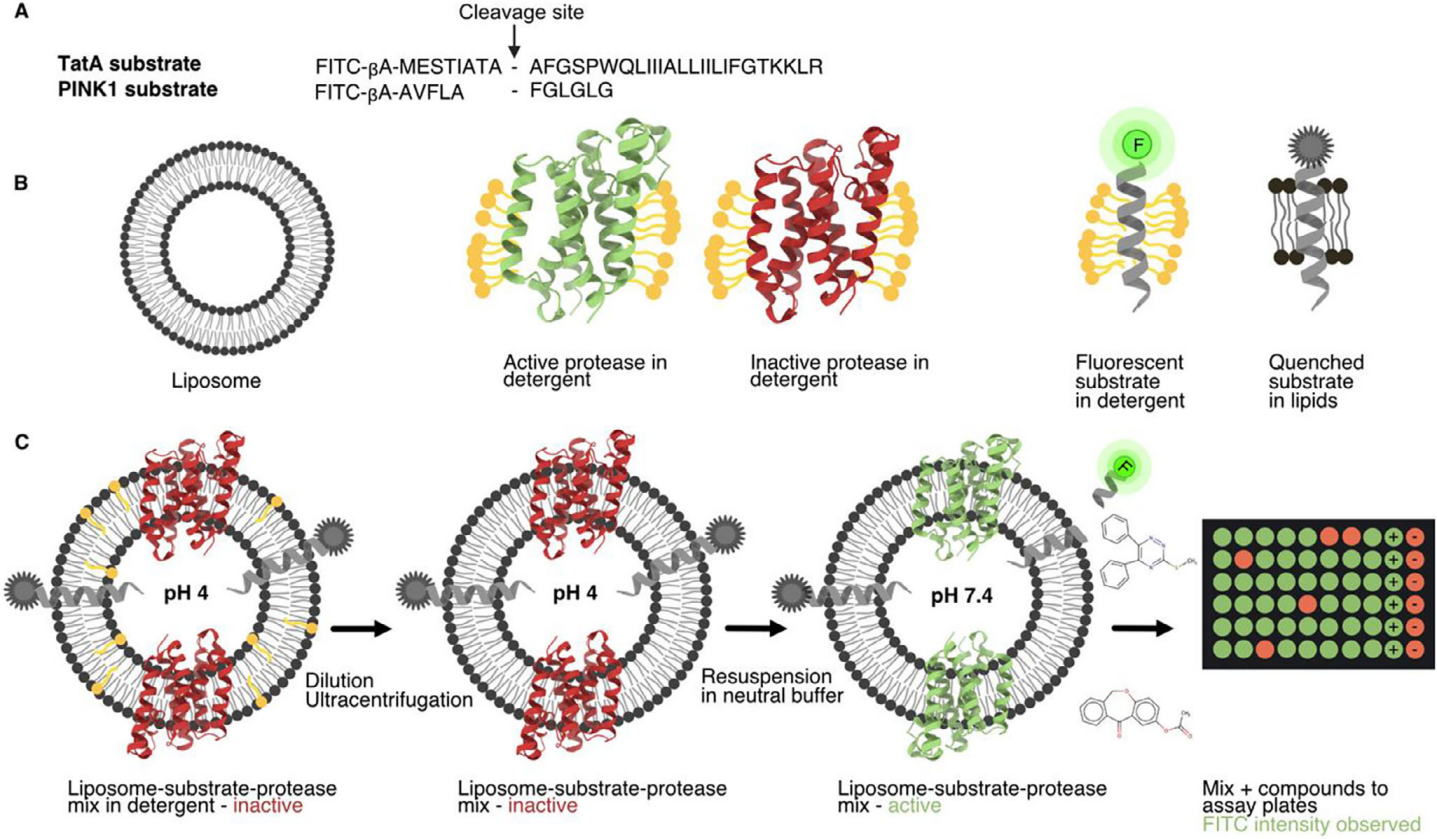
Assay scheme. (A) Sequence of the utilized substrates (TatA and PINK1), each coupled to a fluorescein isothiocyanate (FITC) label with the cleavage site by GlpG (TatA) and PARL (PINK1) indicated. (B) Cartoon representation of the components used in the assay: Liposomes of defined size (grey), active protease in green at pH 7.4, and inactive protease in red at pH 4. The substrate is fluorescent in detergent (neon green dot) but quenched through the proximity of the lipid bilayer (grey dot). (C) Reconstitution of substrate and protease into preformed liposomes at pH 4. Following dilution and ultracentrifugation to remove detergent and form proteoliposomes, the protease is activated by buffer exchange to pH 7.4 (red to green). This allows substrate cleavage within the liposomes, resulting in an increase in fluorescence over time. Liposomes are then added to assay plates preincubated with compounds, and the fluorescence increase is recorded to determine compound activity.

The reversible peptidyl ketoamides currently represent the most promising chemotype with the highest potency and selectivity [45]. Despite some peptidyl ketoamides being active in cells, peptide‐based inhibitors generally suffer from poor druglikeness, with low membrane permeability and limited in vivo stability [47] —key challenges when targeting intramembrane enzymes. Since all of the mentioned compounds are nucleophilic and target the catalytic serine in a mostly covalent manner, we aimed to identify new, selective, non‐peptidic rhomboid protease inhibitors in an unbiased way by employing a high‐throughput screening (HTS) approach across multiple serine proteases [48, 49]. Given that intramembrane proteases are highly dependent on their lipid environment, as they can sense lipid distortion and modulate their lipid environment [50, 51, 52], we used an assay with *E. coli* GlpG and human PARL within liposomes to mimic a more native environment [53, 54], which allowed us to identify compounds that may rely on the hydrophobic membrane context for activity (Figure 1). Previous inhibitors were identified through covalent, mechanism‐based approaches targeting the catalytic serine [11, 37, 38, 55, 56], as well as through high‐throughput screening of fluorogenic peptide/FRET substrates and fluorescence polarisation activity‐based protein profiling [39, 57]. In addition, peptide‐mimicking libraries and structure–activity relationship studies were employed to optimize potency and selectivity [11, 45]. While these strategies successfully identified active compounds, their ability to capture inhibition in a native membrane context may be limited, which is addressed by our liposome‐based high‐throughput screening approach.

Our screening efforts led to the identification of 45 compounds that selectively inhibit GlpG, six of which were analysed in detail. Notably, we identified 14 compounds that were active against the human rhomboid protease PARL, with one showing promise for future chemical optimization. We validated our findings through functional biochemical assays, molecular docking and solid‐state NMR spectroscopy (ssNMR). ssNMR has been successfully applied in numerous studies to investigate the structure, dynamics, ligand and lipid interactions of membrane proteins in liposomal environments, providing insights under native‐like conditions [51, 53, 58, 59, 60].

## 2 Results and Discussion

### 2.1 Screening of 68,288 Compounds Led to 45 Compounds Selectively Inhibiting GlpG

GlpG consists of a six‐transmembrane (TM) helical core and a cytosolic domain of unknown function; both structures have been determined separately by X‐ray crystallography and solution NMR, respectively [6, 10, 61, 62, 63]. We focused on the core domain initially due to technical advantages, such as enhanced expression and simplified NMR analysis [8]. More recently, we have extended our study to the physiologically more relevant full‐length variant [64].

We expressed GlpG core domain (GlpGΔN) in *E. coli* following previously described methods [8, 59]. For the screening campaign, we used an assay in which GlpGΔN, reconstituted into liposomes, processes synthetically produced TatA [65] — a heterologous model substrate labeled with fluorescein isothiocyanate (FITC) — as originally proposed by Baker and Urban and described in detail previously [53, 54]. In this setup, the FITC label is quenched when in close proximity to the membrane. Upon substrate cleavage by GlpGΔN, the FITC‐labeled fragment is released, resulting in a measurable increase in fluorescence (see Figure 1 for a scheme).

In our initial tests of the screening assay, we obtained a Z′‐factor of 0.9, affirming its statistical suitability for the screening campaign [66]. The Z′‐factor is a dimensionless assay quality metric, with Z′ > 0.5 indicating a robust assay. To further assess the robustness of the assay and its suitability for high‐throughput applications, we first tested a small subset of compounds in duplicate (1408 compounds, including FDA‐approved drugs) at a final concentration of 25 µM. Employing the Bland‐Altman method for repeatability analysis, we observed acceptable consistency, as indicated by a coefficient of repeatability (limits of agreement) of 22.9% (Figure S1) [67]. The diagram exhibits a slightly diagonal pattern, suggesting a systematic mismatch between the two replicates, which may explain the marginally unfavorable score. However, subsequent analyses (during the confirmatory screen) did not show any systematic mismatch, leading to an improved score (see below).

**FIGURE 2:**
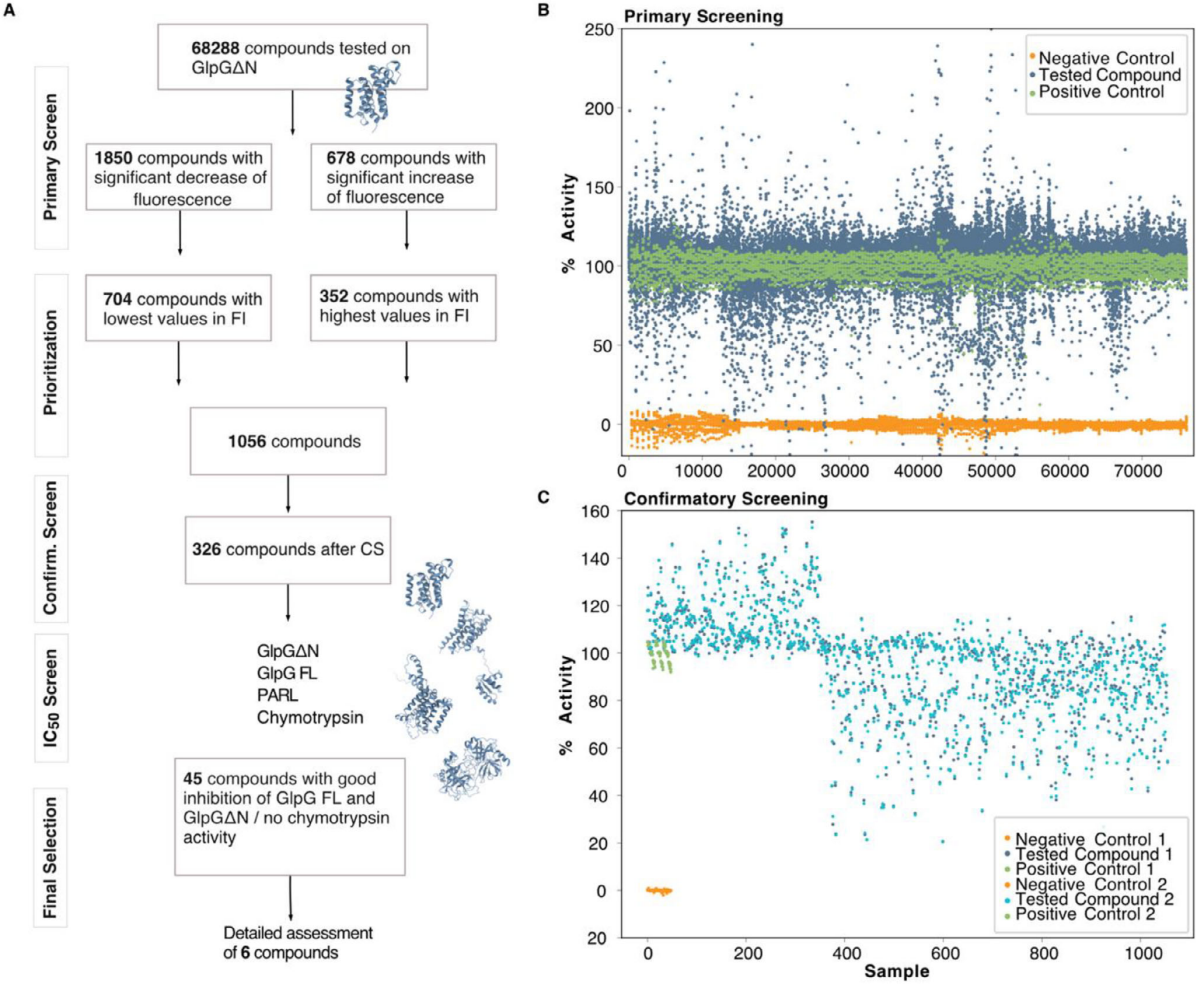
Screening Strategy. (A) Overview of the steps leading to the final compound selection, including primary screening, confirmatory (confirm.) screening, and IC_50_ screening on GlpGΔN, GlpG FL, chymotrypsin, and PARL, featuring a cartoon representation of the 3D structures of these enzymes in blue. FI = fluorescence intensity. (B) Primary screening plot: Percentage activity of GlpGΔN when treated with each of the 68,288 compounds is represented in blue, with the negative control values in orange and the positive control values in green. The sample numbers are arbitrary indices for visualization. (C) Confirmatory screening: GlpGΔN is treated with 1056 compounds. The confirmatory screening was conducted in duplicate, resulting in two subsets of values labeled as 1 and 2 (shown in blue and light blue, respectively).

Following this preliminary assessment, we initiated our screening of 68,288 compounds from our in‐house small‐molecule library (Figure 2A), specifically designed to maximize coverage of chemical space and scaffold diversity [48]. The median Z′‐factor across all plates was 0.7, indicating good separation between positive and negative controls. We did not observe significant enrichment for unfavorable properties such as autofluorescence or cytotoxicity. However, a minor enrichment of redox cycling compounds that demonstrated inhibitory effects was noted (Figure S2).

Notably, 2528 compounds exhibited a significant change in fluorescence levels/protease activity (Figure 2B). For further refinement, we prioritized these compounds based on their highest (147%‐234% increase) or lowest (60%‐26% reduction) values in relative fluorescence (Figure 2A). This was done using Z‐scores, with a threshold of 4. Lower Z‐scores indicate a greater deviation from the assay median, helping us to identify compounds with more pronounced and reproducible effects. After refinement, 1056 compounds were then subjected to confirmatory screening at 25 µM final concentration in duplicate.

After the confirmatory screening, we observed excellent reproducibility with a Z′‐factor of 0.85 and a coefficient of repeatability of 9% (Figures 2C and S3). Applying a threshold of a 20% change in activity, we identified 328 compounds, of which 326 were available for cherry‐picking and were selected for the half‐maximal inhibitory concentration (IC_50_) determination on GlpGΔN in duplicate.

### 2.2 GlpG‐Specific Primary Hits Identified Through IC_50_ and Selectivity Screening

In the IC_50_/counter screening phase (Figure 2A), we introduced additional proteases to evaluate selectivity. Specifically, we utilized the soluble serine protease α‐chymotrypsin from bovine pancreas, in combination with a commercially available kit, where chymotrypsin digests BODIPY‐casein, and the resulting fluorescence is measured.

Additionally, we included the full‐length (FL) variant of GlpG to explore potential differences between the GlpGΔN and the FL variant. We specifically identified compounds that demonstrate strong inhibition on both the FL GlpG and GlpGΔN, while exhibiting no inhibitory effects on chymotrypsin. This step was designed to exclude candidates with a general inhibition of serine proteases. In this manner, we narrowed down the selection to 45 compounds (Table S1). Interestingly, several compounds among the 45 hits contained a benzopyranone core (Table S1), a scaffold previously observed in rhomboid inhibitors [37, 40, 41]. This outcome confirms the ability of our screening approach to detect both established scaffolds and novel chemotypes with potential specificity for rhomboid proteases.

**FIGURE 3:**
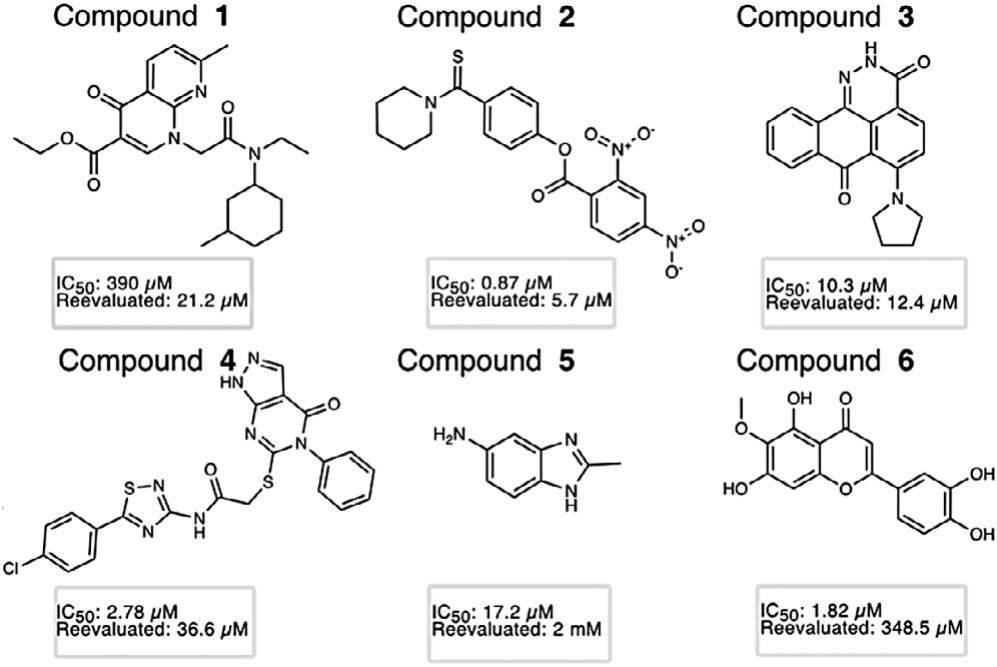
Chemical structures of the 6 final compounds selected for further characterization with favorable inhibition profiles on FL GlpG. The compounds are indicated by their IC_50_ values from the initial screen in liposomes and the reevaluated IC_50_ values in detergent.

From these, we manually selected six molecules with attractive scaffolds, favorable IC_50_ values, and excellent fitted dose‐response correlation (Figures 3 and S4); the most potent was compound **2**, with an IC_50_ of 870 nM. These compounds were selected for more in‐depth analysis. In this context, “favorable IC_50_ values” refer to low values in the µM‐nM range, considering that our reference inhibitors, the isocoumarin JLK6 (7‐amino‐4‐chloro‐3‐methoxy‐1*H*‐2‐benzopyran) and a peptidyl chloromethylketone (Acyl‐RVRHA‐cmk, henceforth CMK) inhibitor, showed IC_50_ values in the range of 6–8 µM [37, 45, 59]. Apart from compound **6**, a natural product featuring a benzopyranone core similar to that of isocoumarins—though lacking an electrophilic center like in JLK6—none of the selected molecules exhibit obvious structural similarity to previously reported rhomboid inhibitors.

We then tested the 6 compounds with FL GlpG in a detergent‐based setup using a non‐membrane substrate as described previously [30, 46]. This substrate consists of a peptide with an optimized sequence (RVRHA) coupled to the fluorescent aminomethyl coumarin moiety, and has been previously validated [30]. Generally, the IC_50_ values were higher than in the primary assay (with the exception of compound **1**), which may be attributed to the altered physicochemical context (liposomes vs. detergent), where the absence of a lipid bilayer could affect the conformational dynamics of GlpG or change the binding mode of the inhibitors, especially those that rely on hydrophobic interactions within the membrane. Compounds **5** and **6** showed poor inhibition in the detergent‐based assay so that they were excluded from further analyses. Compound **3** exhibited poor solubility in the detergent assay, and compound **1** had low activity in the liposome assay, and we thus narrowed down our focus to compounds **2** and **4**, which performed consistently in both assays.

To validate our results in another orthogonal activity assay for the selected compounds, we performed an SDS‐PAGE–based activity assay [8] in liposomes with a FL TatA‐based substrate. In this assay, SUMO–FL TatA was used as the substrate and its cleavage by FL GlpG was monitored on the gel in the presence of selected compounds. Inhibition was seen for every compound tested (Figure S5).

### 2.3 Similarity Screening of Compounds **2** and **4** Indicate Required Binding Motifs

**FIGURE 4:**
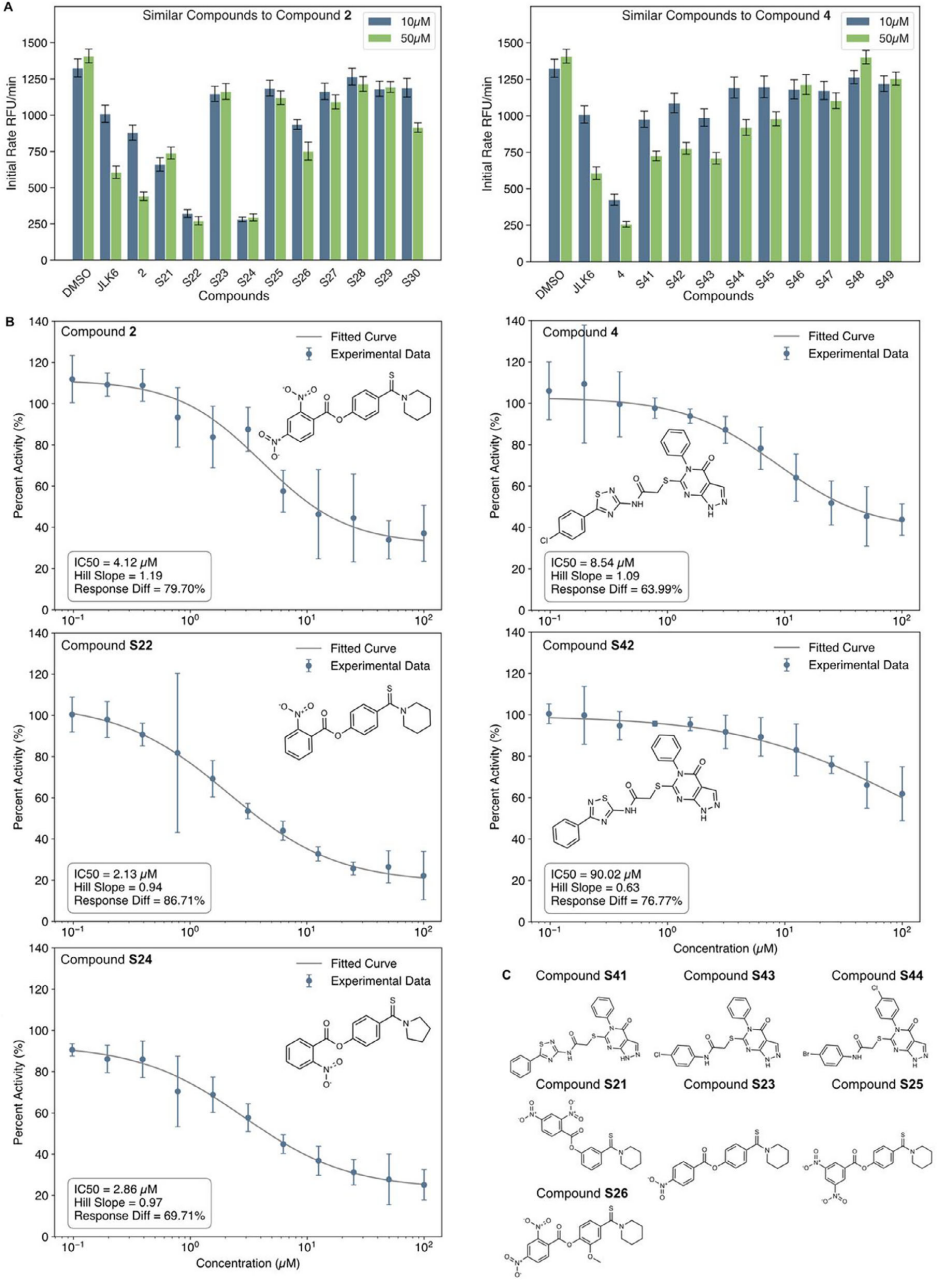
Similarity search for compounds **2** and **4**. (A) Initial rates of RFU (Relative Fluorescence Units) from FL GlpG incubated with different compounds at two concentrations (10 µM, blue and 50 µM, green): DMSO, compound **2** and structurally similar compounds S**21–30**, compound **4** and structurally similar compounds S**41–49**, and control inhibitor JLK6. The experiments had 3 technical replicates, error bars indicate median absolute deviation (MAD). (B) Dose‐response curves (percent protease activity vs. compound concentration) for FL GlpG incubated with different concentrations (0.1 – 100 µM in 1:2 serial dilutions) of compounds **2**, S**22**, S**24**, **4**, and S**42**, showing IC50, Hill slope, and response difference (Diff.), respectively. The experiments had 4 technical replicates, error bars indicate median absolute deviation (MAD). Biological replicates are shown in Figure S6. (C) Chemical structures of compounds similar to compounds **2** & **4** – S**21**, S**23**, S**25**, S**26**, S**41**, S**43**, and S**44**, highlighting the structure‐activity relationship of the compounds.

To explore the structure‐activity relationship (SAR) of compounds **2** and **4** and identify potential avenues for optimization, we searched the PubChem database for structurally similar compounds using FCFP_4 fingerprints [68]. This search yielded 166 compounds with a Jaccard–Tanimoto similarity ≥ 0.7 (range: 0 to 1) for compound **2** and 13 for compound **4**. From these, we manually selected 10 most similar compounds to compound **2** (S**21–**S**30**) and 9 most similar to compound **4** (S**41–**S**49**) for experimental testing (Table S2). All selected compounds were evaluated for their inhibitory activity against FL GlpG using the same assay conditions as described above and compared to the reference inhibitor JLK6 (structurally unrelated, Figure 4A).

Analogues S**21–**S**30** share an ester moiety, which is susceptible for reacting covalently with the catalytic serine of the enzyme via a nucleophilic attack. Enhanced potency in this series correlated with increased electrophilicity of the ester. Among them, S**22** and S**24** showed the strongest inhibition (∼80%), likely due to different positioning of the nitro group, which strongly enhances the reactivity of the ester through both inductive (–I) and mesomeric (–M) electron‐withdrawing effects. By contrast, S**21** (with incorrect ester connectivity) and S**26** (featuring a bulkier methoxy group) displayed only moderate inhibition (∼40–50%). In S**23**, a para‐substituted nitro group should theoretically enhance inhibitory activity via –I and –M effects, but its position may introduce steric clashes or negative repulsion in the binding pocket, diminishing inhibitory activity. Similarly, compound **2** showed weaker inhibition (∼70%) than S**22** and S**24**, likely due to steric interference or unfavorable interactions from its second nitro group. For compound S**25,** with two nitro groups in the meta‐position, only weak –M effects are transmitted to the ester, resulting in diminished activity. Compounds S**27–**S**30** (Table S2), which contain mispositioned esters or weaker electron‐withdrawing groups, showed no significant inhibition.

For the analogues of compound **4** (with compound **4** at ∼80% inhibition), only mild inhibitory effects were observed for S**41–**S**43** (50% inhibition), while S**44–**S**49** (Table S2) showed no significant inhibitory activity (Figure 4A). Notably, compounds S**43**‐S**45** showed reduced inhibition most likely due to the missing thiadiazole ring and the lower hydrophobic interaction with only a single phenyl ring instead of chlorophenyl. In case of compounds S**46**‐S**49** the scaffold change from a pyrazol‐pyrimidin‐4‐one ring to 5‐phenyl‐thieno‐pyrimidin‐4‐one ring resulted in lower inhibitory activity, probably because of a steric clash of the additional phenyl ring at the 5‐position.

To further evaluate these compounds, we conducted manual IC_50_ assays again on compounds **2**, **4**, as well as S**22**, S**24**, and S**42** (Figures 4B and S6) under the same assay conditions as for the IC_50_ screening phase. Compounds S**22** and S**24** demonstrated better dose‐response behaviors than compound **2** (IC_50_ = 2.13 and 2.86 µM vs. 4.12 µM) based on the compensation of the stronger electronic effects (see above). Interestingly, replacing the piperidine ring with pyrrolidine (as seen in S**24**) did not significantly reduce potency, but overall, the response difference is lower than for compound S**22**. For compound **4** (IC_50_ = 8.54 µM), the chlorophenyl and thiadiazole rings appear to be essential motifs, as their removal or substitution—e.g., replacing the chlorophenyl group with a phenyl—led to diminished inhibitory activity (IC_50_ = 90.2 µM for compound S**42**).

Note that JLK6, used here as a reference inhibitor, exhibited lower potency in the liposome‐based assay compared to a detergent‐based setup (IC_50_ ∼21 µM vs. 6 µM [37], respectively), although its maximal inhibitory efficacy remained unchanged (Figure S7). This observation underscores the importance of identifying potential inhibitors under more physiologically relevant conditions that better reflect the native membrane environment of the protease.

### 2.4 Biochemical Characterization of Compounds and their Analogues Reveals Reversible Covalent Inhibition of GlpG In Vivo

**FIGURE 5:**
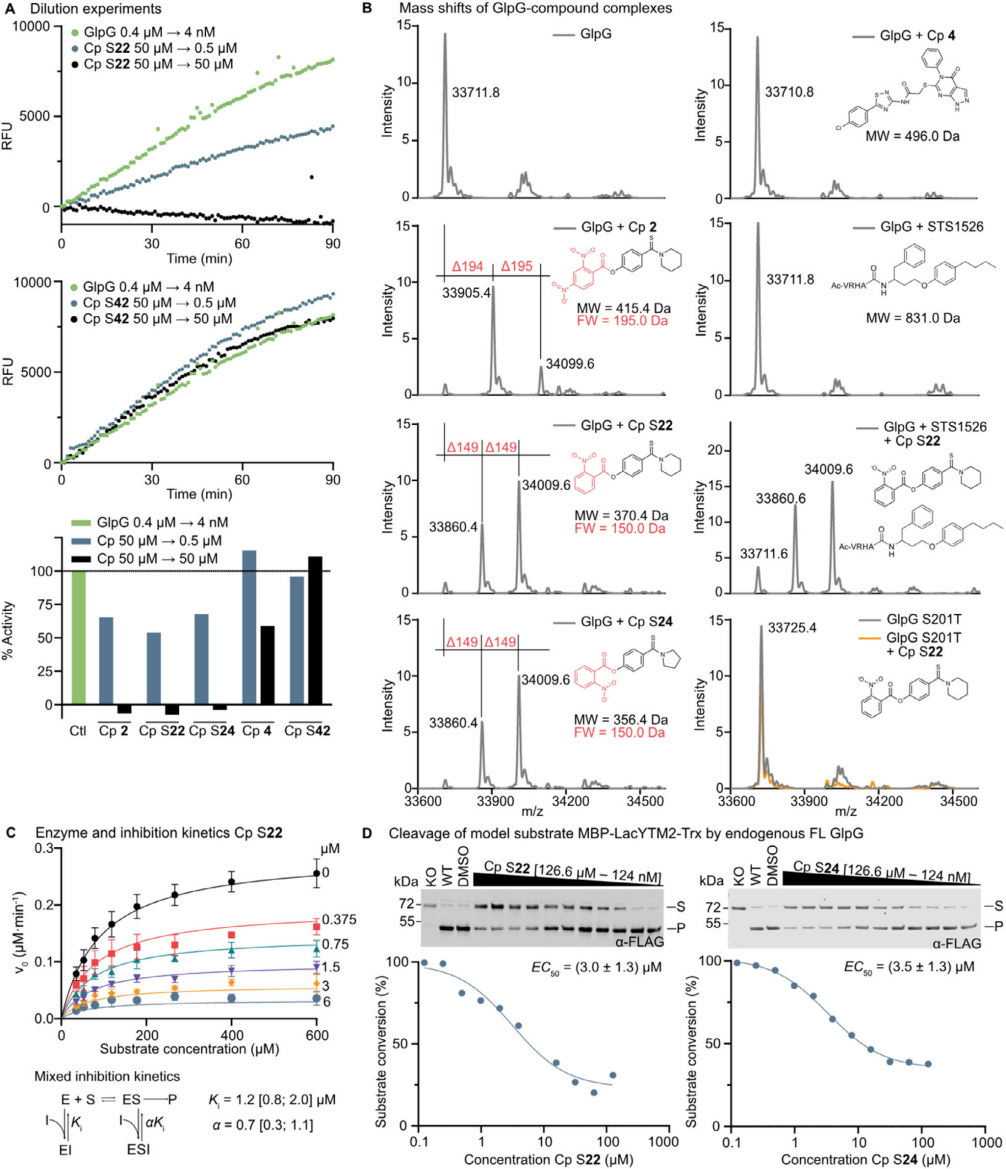
Mechanism of action of compounds **2** and **4** and their structural analogues. (A) Reversibility of inhibition of compound (Cp) **2**, S**22**, S**24**, **4**, and S**42** was analy*s*ed using dilution experiments, monitored as changes in relative fluorescence units (RFU). The top and middle panels show time courses of fluorogenic peptide substrate [46] cleavage by 4 nM FL GlpG. 0.4 µM FL GlpG alone was diluted to 4 nM GlpG as a control (green trace), 0.4 µM FL GlpG was preincubated with 50 µM inhibitor and diluted to 4 nM FL GlpG in the continued presence of 50 µM inhibitor (black trace), and 0.4 µM FL GlpG was preincubated with 50 µM inhibitor and diluted to 4 nM GlpG and 0.5 µM inhibitor (blue trace). Initial reaction rates (calculated as percent enzyme activity (% activity)) were determined in this manner for all indicated inhibitors (compounds **2**, S**22**, S**24**, **4**, and S**42**), and the remaining time courses are shown in Figure S8. (B) Covalent enzyme–compound complex formation was assessed by intact mass spectrometry. FL GlpG or FL GlpG S201T was incubated with the indicated inhibitors (compounds **2**, S**22**, S**24**, **4**, and STS1526 with respective structures and molecular weight (MW)) at an eightfold molar excess and subsequently analyzed by ESI mass spectrometry. Inhibitor‐induced mass shifts are highlighted in red, and the substructure of inhibitor corresponding to each observed mass difference is highlighted in red with its corresponding fragment mass (FW). (C) The inhibition mechanism of compound S**22** was determined by steady‐state enzyme kinetics. Initial reaction rates of fluorogenic peptide substrate cleavage by FL GlpG were measured across a range of substrate concentrations (35.1 µM to 600 µM) extending up to sixfold above K_M_ and at multiple inhibitor concentrations (0, 0.375, 0.75, 1.5, 3 and 6 µM). Global nonlinear fitting of the data showed that a mixed‐type inhibition model provided the best fit. The resulting K_i_ value and the parameter α are reported with 95% confidence intervals. (D) Inhibitory potency of compounds S**22** and S**24** in live *E. coli*. Cleavage of model substrate MBP‐LacYTM2‐Trx [45] by endogenous FL GlpG was followed by immunoblotting at a range of inhibitor concentrations (0.124 – 126.582 µM in 1:2 serial dilutions). The EC_50_ curves were derived by densitometry based on quantitative, near‐infrared fluorescence detection of the substrate (S) and product (P) bands as described previously [45]. Reported EC_50_ values are averages ± standard deviation from four biological replicates (all data available in Figure S9). KO = FL GlpG knockout strain, WT = untreated, DMSO = treated with DMSO.

We next examined the mechanism of action of compounds **2** and **4**, and their analogues biochemically. First we inspected whether compounds **2, 4,** S**22,** and S**24** (and S**42** as a negative control) bind reversibly to GlpG using dilution experiments reported previously [45]. GlpG was pre‐incubated with each compound at 50 µM (∼10× IC_50_) and then diluted 100‐fold into reaction buffer containing either 0.5 µM (∼0.1× IC_50_) or 50 µM inhibitor, together with a fluorogenic substrate [46]. All four compounds behaved as reversible inhibitors, while no inhibition was seen for S**42** (Figures 5A and S8). Progress curves in the presence of inhibitor were linear (Figure S8), indicating fast‐binding kinetics, in contrast to the slow‐binding ketoamides reported previously [45].

To determine whether inhibition involves covalent chemistry, we analysed FL GlpG by denaturing intact mass spectrometry (Figure 5B). Incubation of GlpG with compound **4** did not produce additional mass peaks, suggesting either non‐covalent binding or an unstable covalent adduct. In contrast, exposure to compounds **2**, S**22**, and S**24** produced stable adducts with mass increases of 194 ± 1 Da (compound **2**) or 149 ± 1 Da (compounds S**22** and S**24**), consistent with attachment of the dinitrobenzoyl (compound **2**) or nitrobenzoyl (compounds S**22**, S**24**) fragments (substructures shown in red in Figure 5B). Interestingly, two adducts were detected in each case. Co‐incubation of the active‐site targeting ketoamide STS1526 (compound 5 [46]) with compound S**22** led to a higher fraction of the unmodified GlpG than in the absence of STS1526, indicating competition of the compounds for the active site. In addition, we analysed the S201T catalytic mutant of GlpG, which retains a hydroxyl at the position of the catalytic serine but lacks polarisation by H254, and is structurally intact but proteolytically inactive [9]. Incubation of GlpG S201T with S**22** produced no adducts, demonstrating that covalent bond formation requires an activated S201, thus targeting the catalytic dyad, and that the second adduct is dependent on the initial acylation step in the active site. The molecular identity and location of the second modification site could not be resolved at this stage and therefore remain an open mechanistic question. While the data indicate active‐site‐directed covalent engagement, the occurrence of a second adduct highlights a potential optimisation liability that will require further investigation. These observations collectively indicate that compounds **2**, S**22**, and S**24** act as reversible covalent inhibitors of GlpG.

Kinetic analysis of the inhibition mechanism of compound **S22** (performed as described previously [45]) showed that it behaves as a mixed‐type inhibitor (Figure 5C), meaning that it interacts both with empty enzyme as well as with the enzyme‐substrate complex. This mechanism is likely shared by its close structural analogues, compounds **2** and **S24**. In summary, compounds **2,** S**22,** and S**24** act as covalent, reversible, mechanism‐based mixed‐type inhibitors of GlpG.

The activity of compounds **4, 2,** S**22**, and S**24** on endogenous GlpG was evaluated in the *E. coli* K‐12 MC4100 derivative NR698 strain, which has a genetically permeabilized outer membrane [69]. The glpG::tet derivative strain [39] served as a negative control, and the LacYTM2 reporter substrate was used as described previously [45]. In this cellular assay, compounds **4** and **2** showed no detectable activity (Figure S9), whereas compounds S**22** and S**24** exhibited EC_50_ values of approximately 3 µM (Figure 5D), close to the previously determined IC_50_ values. However, neither compound S**22** nor S**24** inhibited GlpG in wild‐type *E. coli* MC4100 with an intact outer membrane (data not shown), indicating that these compounds exhibit poor penetration through the native *E. coli* outer membrane. This limitation is consistent with earlier observations for β‐lactam‐based rhomboid inhibitors [39] and for the early ketoamide inhibitors [45], both of which required further structural optimization to improve permeability [30, 46].

### 2.5 Selectivity Analysis Reveals Specificity for GlpG and Compounds Targeting PARL

To confirm that compounds **2**, S**22**, S**24**, and **4** selectively target rhomboid proteases, we assessed their binding to a panel of soluble serine proteases (chymotrypsin, trypsin, subtilisin, proteinase K, and elastase) using the TAMRA–FP activity‐based profiling assay with in‐gel fluorescence, as described previously [70]. Compounds **2,** S**22**, and S**24** showed robust inhibition of GlpG but did not inhibit any of the additional serine proteases tested (Figure S10). Compound **4** did not appear to inhibit GlpG in this assay, most likely because the fluorophosphonate probe outcompetes compound **4** due to its low IC_50_ and the reversible, non‐covalent nature of its interaction with GlpG. While these results indicate selective engagement of rhomboid proteases in vitro, confirmation of high selectivity will require broader proteome‐wide evaluation.

To evaluate selectivity of the compounds against other rhomboid proteases, we assayed whether some of the identified compounds active on GlpG also inhibit human rhomboid protease PARL, which is also a relevant pharmacological target. To this end, we included PARL in the IC_50_ screening phase, testing the selection of 326 compounds identified after the GlpG confirmatory screen. We produced enzymatically active PARL in *Pichia pastoris* (*P. pastoris*) following an established protocol [71, 72] and performed an activity assay similar to the one used for GlpG but instead using PINK1 as a model substrate [71, 73, 74] (Figure 1). From the 326 tested compounds, we identified 14 that showed inhibitory activity against PARL (IC_50_ = 3.5–15.8 µM). These compounds exhibited favorable IC_50_ values, clear dose–response relationships, and no off‐target activity against chymotrypsin (Table S3).

We additionally evaluated the 45 GlpG‐prioritized compounds for PARL activity. Of these, 7 also inhibited PARL, and their corresponding IC_50_ values are provided in Table S1 (notably, some compounds that were effective against PARL did not show strong inhibition of GlpG and are therefore not present in Table S1). From our refined selection of 6 compounds with inhibitory effects on GlpG, compound **4** was also active on PARL (IC_50_ = 6.26 µM). To identify additional compounds with high specificity for PARL and minimal off‐target activity on other rhomboid proteases, a broader screen of the entire compound library will be required and further work will be needed to explore proteome‐wide selectivity and to confirm activity in cellular contexts.

### 2.6 Docking and Modeling Analyses Unveils Potential Binding Modes of Compounds

**FIGURE 6:**
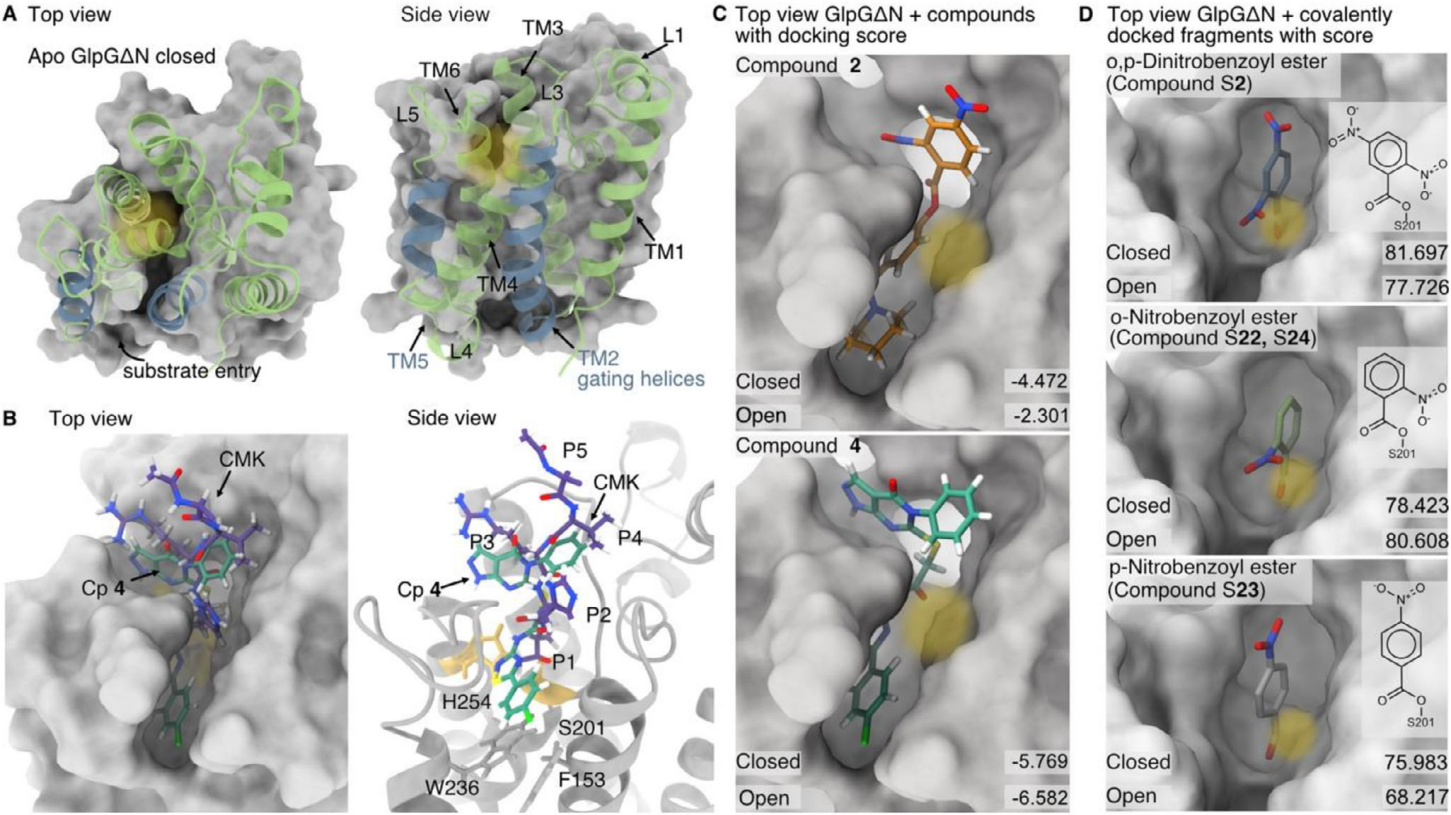
Docking analysis of compounds **2,** S**22,** S**23,** S**24, 4**, and **CMK** to GlpGΔN. (A) Top and side view of GlpGΔN (PDB ID: 2NRF, prepared for docking) in the closed conformation as grey surface representation, and the secondary cartoon structure in green mapped onto it. Transmembrane helices (TM) are highlighted, with gating helices in blue and active site location marked with a yellow shade, substrate entry site marked with an arrow. (B) Comparison of compound **4** (green, Cp **4**) to the X‐ray structure of an optimized peptide‐based inhibitor (purple, peptidyl chloromethylketone, CMK, PDB ID: 5MT8) [45] bound to GlpGΔN (surface representation, top view). Right: GlpG is depicted in the cartoon side view with the active site (S201 and H254) highlighted in yellow, along with gate residues (W236 and F153) in grey sticks. The P1‐P5 substrate‐binding sites are also highlighted. (C) Docking of compounds **2** and **4** to GlpG in the closed conformation, displayed as a top view of the surface with colored compounds in stick representation, including the docking scores (Glide) for compounds **2** and **4** docked to the open and closed conformations. The approximate location of the active site serine is marked with a yellow shade. (D) Covalent docking of compound fragments in ortho, para, and ortho/para dinitro/nitro group configurations (representing compounds **2**, S**22**, S**23**, S**24**) to GlpGΔN in the closed conformation, displayed as a top view of the surface with colored compounds in stick representation, including the docking scores (GOLD PLP fitness) for compound fragments docked to the open and closed conformation. The approximate location of the active site serine is marked with a yellow shade.

To explore the mechanism of inhibition in molecular terms, we conducted molecular docking studies for compounds **2** and **4** and analogues (Figure 6A–C) taking into account the biochemical data, as previously described [59]. For this, the three‐dimensional structures of GlpGΔN in both the open and closed states were extracted from the crystallographic structure (Figure 6A, PDB ID: 2NRF) [6]. Subsequent molecular docking was carried out using a receptor grid of 20 Å centered around the active site S201.

We first analyzed docking poses of compound **4** and the intact form of compound **2** to explore whether the latter could adopt a productive binding mode prior to ester cleavage and covalent modification as seen in biochemical experiments. The analysis of the docking poses indicates that compounds **2** and **4** occupy a narrow pocket on the extracellular side adjacent to the catalytic site, similar to known rhomboid protease inhibitors such as isocoumarins [37], benzoxazinones [41, 42], and peptide‐based covalent inhibitors [11, 27, 30, 44, 45, 46] (Figure 6B,C). The Glide docking scores, ranging from −2.3 to −6.6 (Figure 6C), suggest favourable but relatively weak binding, consistent with the limited number of hydrogen bonds and salt bridges formed between the protein and the ligands in this pocket. Notably, the docking scores obtained for both the open and closed states were similar for all compounds; therefore, no definitive conclusion could be drawn whether the compounds preferentially bind the open or closed state.

**FIGURE 7:**
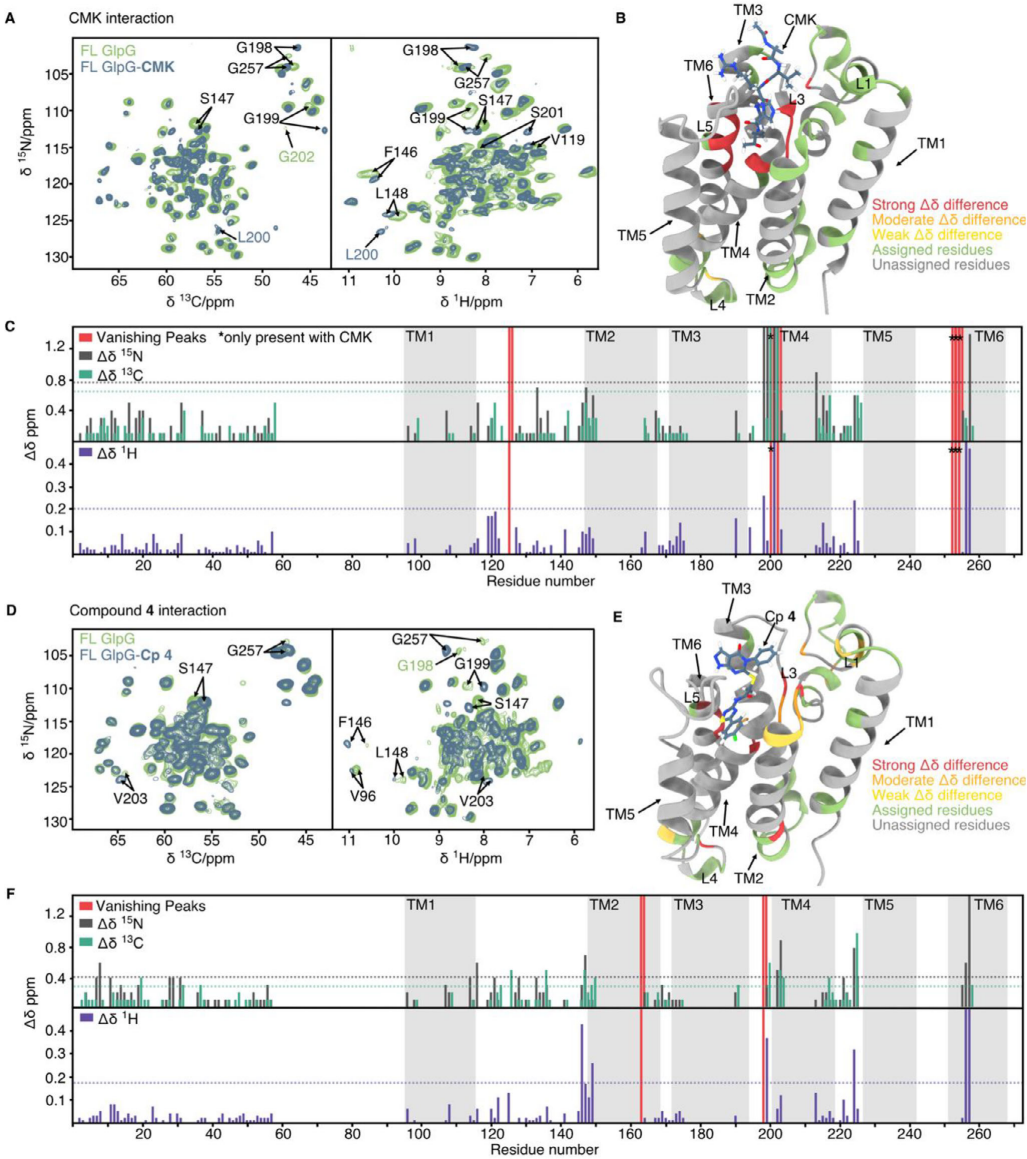
Ligand binding analysis of GlpG with CMK and compound **4** using ssNMR. (A, D) hCANH CN and HN projections for FL GlpG bound to CMK (A) and compound **4** (D), respectively, where the spectra of the ligand‐bound state are denoted with blue and the unbound state green. Amino acids with significant chemical shift changes are indicated by arrows and labeled in black if present in both spectra, and in green/blue if detected only in the unbound/bound state, respectively. (B, E) Residues exhibiting chemical shift differences are mapped onto the crystal structure of GlpGΔN bound to CMK (B, PDB ID: 5MT8) and the docking model bound to compound **4** (E, PDB ID: 2NRF). The magnitude of chemical shift differences is categorised as described in the methods section and color‐coded as follows: strong in red, moderate in orange, and weak in yellow. Green labels indicate assigned residues that do not meet these thresholds. (C, F) Bar plots of absolute chemical shift difference for the ^15^N, ^13^Cα, and ^1^H dimensions of bound and unbound state for CMK and compound **4**, respectively. All values were derived from Tables S4–S9. Peaks only present in the CMK sample are marked with a star. Dashed lines indicate the statistical threshold used to classify significant chemical shift changes (mean + 1 SD).

To assess the structural arrangement of compound **4**, we compared its predicted docking pose with the crystal structure of the reference pentapeptidyl chloromethylketone inhibitor CMK (PDB ID: 5MT8) (Figure 6B) [11, 45]. It consists of an amino acid sequence preferred by GlpG terminated by a chloromethylketone warhead that binds covalently to the active site residues S201 and H254 forming an irreversibly bound complex. Structural analysis showed that CMK forms stabilizing main‐chain hydrogen bonds with the L3 and L5 loops of GlpG, as well as hydrogen bonds involving the side chains of the strongly preferred arginine and histidine at the P3 and P2 positions, respectively [45]. Note that the binding of this compound induces a rather closed conformation, which is why we selected the closed conformation for all figures (Figures 6 and 7). Notably, the docking poses of compound **4** exhibited considerable similarity to that of the reference compound (Figure 6B,C). The chlorophenyl ring is positioned close to the lateral gate residues of the enzyme (e.g. F153 and W236, Figure 6A,B), while the phenyl of the pyrazol‐pyrimidin‐4‐one ring likely interacts with loop L3 and the thiadiazole ring with oxyanion residues H150 and N154 (Figures 6B, S11, and S12 for a more detailed view). Consistent with biochemical data demonstrating that compound **4** acts as a non‐covalent and reversible inhibitor, we propose that it occupies this pocket predominantly through hydrophobic interactions, thereby sterically blocking substrate access to the active site. In agreement with this model, compound **4** can effectively compete with the substrate but does not displace an activity‐based probe such as TAMRA‐FP (see above).

Although docking of intact compound **2** was initially performed to probe a potential pre‐reaction binding mode, biochemical experiments demonstrate that compound **2** undergoes ester cleavage, with the resulting nitrobenzoyl fragment forming the covalent adduct with GlpG. We therefore re‐evaluated the docking of compound **2** and its analogues (compounds S**22**, S**23**, S**24**) at this stage. Specifically, we docked the dinitrobenzoyl and nitrobenzoyl fragments in ortho, para, and ortho/para nitro group configurations (see Figure 6D). All three variants could be successfully docked into the GlpG active site using a covalent restraint to Ser201, in both the open (PDB ID: 2NRF, chain A) and closed (PDB ID: 2NRF, chain B) conformations. The resulting poses were highly similar across all three nitro configurations in both conformational states of GlpG (Figure 6D). In the closed conformation, the ortho nitro group consistently formed a hydrogen bond with M249 of GlpG. Docking of the para nitro variants (corresponding to compound S**23**) yielded slightly lower GOLD PLP fitness scores (75.98), whereas the ortho nitro (78.42; compounds S**22** and S**24**) and ortho, para dinitro (81.70; compound **2**) variants scored higher for the closed variant. These trends are consistent with the results from the IC_50_ assays. It is important to note that any differences in reactivity between the para nitro and ortho nitro/ortho, para dinitro compounds, particularly those arising from transition‐state effects during covalent bond formation, cannot be captured by the covalent docking protocol used here.

### 2.7 Docking of Compound **4** to PARL Suggests Binding near the Active Site

To explore the mechanism underlying the activity of compound **4** against PARL, we conducted molecular docking using the AlphaFold3 inference pipeline to directly model the enzyme‐ligand complex [75]. The observed pose is consistent with prior predictions for optimized PARL inhibitors such as 4‐oxo‐β‐lactams [76].

In the optimal docking pose of compound **4**, the thiadiazole ring and the carbonyl oxygen of the amide linker are oriented toward the catalytic dyad (S277 and H335). Whilst compound **4** occupies a similar position relative to the active site in both PARL and GlpG, its orientation differs (Figures 6B and S13). Based on these predictions, its chemical structure, the relatively weak IC_50_, and previous biochemical results on GlpG, covalent interaction seems unlikely to us. Instead, a hydrophobic blockage of the cleft, hindering substrate binding, seems more plausible. It is important to note that the 3D structure of PARL was predicted in silico, and the interpretation of these docking results should therefore be regarded with caution. Further experimental studies, such as X‐ray crystallography, are necessary to confirm the proposed binding mode and inhibitory mechanism.

### 2.8 Solid‐State NMR Analysis of GlpG with Compounds **2**, **4**, and CMK Reveals Structural Changes

To gain further atomistic insights into the inhibition mechanism of the compounds and to validate the docking results, we conducted a proton‐detected solid‐state NMR analysis of FL GlpG with compounds **2** and **4**. Additionally, CMK was included as a reference inhibitor, as compound **4** was predicted by the docking to bind in a similar manner and at a similar location as observed in the crystal structure of the GlpGΔN–CMK complex.

For the FL GlpG‐CMK complex, we recorded a series of hCANH, hCONH, hCAcoNH, and hCOcaNH 3D spectra of ^2^H,^13^C,^15^N—labelled FL GlpG to perform assignments via a backbone‐walk (Table S7). For an accurate comparison between bound and unbound state, a reference hCANH spectrum was recorded under the same conditions as for the bound state. The chemical shifts of the reference spectrum were identified based on previously published assignments [64] (Table S6). Comparing the chemical shifts of FL GlpG and FL GlpG‐CMK revealed significant chemical shift differences, which can be directly observed in the hCANH CN and HN projections (Figure 7A). These correspond to a change in chemical environment of the shifted residues, indicating binding/interaction of CMK at or near this location. For FL GlpG‐compound **4**, we recorded hCANH and hCONH 3D spectra. Direct comparisons with both its respective reference spectrum (Table S4) and FL GlpG‐CMK spectra enabled the assignment of all residues listed in Table S5. The hCANH CN and HN projections shown in Figure 7D exhibit similar chemical shift changes in the bound state compared to the reference, though with lower absolute magnitude.

To characterise the interactions in more detail, we calculated the absolute chemical shift differences (Δ*δ*) for each dimension (^15^N, ^13^Cα, and ^1^H) for CMK and compound **4** (Figure 7C,F) compared to the unbound state. For CMK, the respective signals of W125 and G202 are absent in the bound state spectra. In contrast, peaks of residues L200, G252, A253, and H254 (residues around the active site) are absent in unbound state spectra but appeared upon CMK binding. Additionally, we observed large chemical shift changes for residues G198, G199, S201, A256, and G257, which also belong to residues around the active site. Note that residue A256 was assigned based on prior 4D NMR experiments, and this assignment remains tentative in the current analysis. The spectral changes observed in our study may be attributed to hydrogen bonding involving G198 and G199, consistent with earlier findings from the GlpG–CMK complex [45]. Based on the previous structure, loop L5 residues (S248, M249, and A250) also participate in hydrogen bonding. We cannot unambiguously assign those residues in our spectra, possibly because of conformational heterogeneity or dynamics. However, the pronounced chemical shift changes observed for neighboring residues suggest an interaction between the ligand and loop L5. We further detected changes in loop L1, specifically for residues F146, S147, and L148. F146 has been reported to interact with the substrate through van der Waals contacts, implicating loop L1 as an S4 subsite essential for GlpG function and substrate specificity [11]. These differences are highlighted in the 2D projections of the hCANH 3D experiment (Figure 7A). Overall, our findings are consistent with previous observations [45].

To systematically assess the magnitude of chemical shift differences (Δ*δ*), we categorised residues based on their deviation from the average shift (Table S9). We mapped these changes, if seen for any of the ^15^N, ^13^Cα, and ^1^H dimensions, onto the crystal structure of GlpG bound to CMK (PDB ID: 5MT8) (Figure 7B). This revealed that the strongest differences occur near the active site. Note that, due to the large overall variation in chemical shifts, no residues from TM2 or loop L1 met the predefined significance thresholds.

For compound **4**, we similarly calculated and mapped chemical shift differences onto the GlpG structure (PDB ID: 2NRF). Chemical shift changes appear for residues within TM2, loop L3, L4, and TM6 (with the peaks corresponding to residues G163 and G198 disappearing) (Figure 7F). Note that G163 has a very low intensity in both reference and bound state spectra and therefore should be considered with caution. Overall, these differences are not as pronounced as those observed for CMK potentially indicating overall weaker binding of compound **4** than CMK. Additionally, significant chemical shift changes occur at the end of loop L1 and the beginning of TM2. Some of these residues (F146, S147, L148) can be directly observed in the hCANH projections (Figure 7D), as previously reported for CMK. Here, the smaller standard deviation values and the larger chemical shift differences across different regions of the protein indicate that not only residues near the active site play an important role, but also those in loop L1 and TM2 (Figure 7E).

CMK is known to form a covalent bond with GlpG, whereas compound **4** is expected to interact non‐covalently, as suggested by our biochemical analysis. This difference in binding mode likely contributes to the varying magnitude and distribution of chemical shift differences observed. Comparing CMK and compound **4**, the strongest chemical shift differences can be seen in the loop regions L1 (W125), L3/TM4 (G198, G199) and L5/TM6 (G252, A253, H254, G257). CMK binding induces stronger changes in these regions. In contrast, compound **4** causes more prominent shifts in L1/TM2 residues F146, S147, and L148, as well as loop L3 residue G198. In contrast to CMK, compound **4** stretches out into TM2/TM5, which might explain these differences. Notably, both compounds induce changes in loop L3, a region where CMK has been shown to form a parallel β‐sheet interaction [11, 45]. It is possible that compound **4** interacts with this region in a similar manner. Importantly, the chemical shift perturbations observed for compound **4** overlap with residues lining the lateral gate and oxyanion region, consistent with the docking model. The differences between CMK and compound **4** support distinct yet spatially related binding modes, while the shared L3 perturbations suggest a common structural response in this functionally critical region. Interestingly, while both compounds induce chemical shift changes in similar regions, the direction of these shifts differs in some cases. See for example, the G199 NH crosspeaks in Figure 7A (right panel, CMK) and Figure 7D (compound **4**). These differences highlight the sensitivity of NMR chemical shifts to the electronic environment and suggest subtle differences in binding modes.

Next, we performed the same ssNMR analysis for compound **2** (Tables S4, S8; Figure S14). The chemical shift differences observed were less pronounced compared to compound **4**. Strong chemical shift changes were detected only for G199 and G257 (Figure S14), however a few other residues—previously reported for CMK and compound **4—**showed only minor changes. Overall, the pattern of differences was similar to that of compound **4** and CMK, but weaker in magnitude. Biochemical analysis indicates that only a fragment of compound **2** forms a covalent bond with S201 of GlpG. This partial engagement likely explains the relatively small chemical shift perturbations observed in ssNMR. The weaker shifts compared to compound **4** and CMK further suggest that compound **2** induces a less extensive structural rearrangement in GlpG, consistent with the less bulky structure of its binding fragment.

## 3 Summary and Conclusion

Rhomboid proteases are attractive therapeutic targets due to their involvements in diverse biological pathways and their increasingly recognised roles in disease. However, the development of selective inhibitors remains a major challenge, largely because of their highly conserved active site topology. This difficulty is further complicated by the limited structural information available and the fact that rhomboid proteases function within the hydrophobic membrane environment, which complicates both structural and biochemical studies.

Here we present a screening strategy and findings from an HTS designed to identify non‐peptidic rhomboid protease‐selective inhibitors using a native‐like membrane environment and substrate. This physiologically relevant assay design led to the discovery of several chemically diverse compound classes with selectivity for the evolutionarily distinct rhomboid protease GlpG over soluble serine proteases. Among them, compound **4** demonstrated notable potency against both GlpG and PARL, making it a promising scaffold for further chemical optimisation.

In addition, the analogues of compound **2** (i.e., compounds S**22** and S**24**) represent a distinct class of reversible, covalently binding inhibitors that show inhibitory activity in live *E. coli* cells with permeabilized outer membranes (but not **2** itself). These compounds highlight the feasibility of targeting intramembrane proteases with covalent yet reversible chemotypes and underscore the importance of membrane permeability as a key parameter for future optimization.

By integrating molecular docking, solid‐state NMR spectroscopy, and structure–activity relationship analysis of closely related analogues, we provide mechanistic insight into the binding modes of these inhibitors and their interaction with functionally important regions of the rhomboid active site. This multimodal approach demonstrates how membrane‐context screening can be coupled with structural methods to inform inhibitor design for intramembrane enzymes.

Although this study primarily focused on the bacterial rhomboid GlpG, it also establishes a framework for future work on the human mitochondrial protease PARL. Despite their low overall sequence similarity (∼20%), GlpG and PARL share a highly conserved catalytic center, including the serine–histidine dyad and the oxyanion hole (Figure S13). For this reason, GlpG served as a robust model enzyme to establish the assay and a proof of principle for identifying cross‐reactive chemotypes. The PARL experiments presented here were therefore exploratory rather than comprehensive. A dedicated high‐throughput screen against PARL—using variants of its native substrates PINK1 and PGAM5—will be essential for defining PARL‐selective inhibitors and elucidating their therapeutic potential.

Together, these findings lay the foundation for the development of more potent and selective rhomboid inhibitors, with potential applications as both therapeutic agents and mechanistic probes.

## Author Contributions

The article was written through contributions of all authors.

## Funding

This work was supported by the Leibniz‐Forschungsinstitut für Molekulare Pharmakologie (FMP) and the Deutsche Forschungsgemeinschaft (DFG, German Research Foundation) under Germany´s Excellence Strategy—EXC 2008/1 (UniSysCat) – 390540038 (to T.U., Ha.Su., and A.L.). C.Ö. acknowledges funding from the Human Frontier Science Program LT000303‐2019‐L. K.S. acknowledges funding from the Ministry of Education, Youth and Sports of the Czech Republic (InterCOST grant no. LUC23180).

## Conflicts of Interest

The authors declare no conflict of interest.

## Supporting information




**Supporting File 1**: The authors have cited additional references within the Supporting Information.

## Data Availability

The data that support the findings of this study are available in the Supporting Information of this article.
