# Supplementary material for: Targeting the Membrane‐Embedded Rhomboid Protease GlpG: A Multimodal Strategy for Inhibitor Discovery and Mechanistic Insight
Source: Angew Chem Int Ed Engl. 2026 Jan 28;65(10):e14067. doi: 10.1002/anie.202514067 (PMC12955534; doi:10.1002/anie.202514067)
Supplement: Supplementary file 1 — Supporting File 1: The authors have cited additional references within the Supporting Information. [file ANIE-65-e14067-s001.docx]

**Supplementary information** **for the manuscript:**

**Targeting the Membrane-Embedded Rhomboid Protease GlpG: A Multimodal Strategy for Inhibitor Discovery and Mechanistic Insight**

**Authors**: Claudia Bohga, Yurii Dubanych^b,c^, Spyridon Kosteletos^a^, Taoran Xiao^a^, Martin Neuenschwander^d^, Tillmann Utesch^e^, Michael Lisurek^e^, Carl Öster^a^, Andreas Oder^d^, Carola Seyffarth^d^, Kathrin Bach^b^, Denise-Liù Gracias Leone^b^, František Filandr^b^, Marc Wegertf, Sascha Lange^a^, Henry Sawczyc^a^, Jens Peter von Kries^d^, Christian P. R. Hackenberger^f,g^, Edgar Speckerh, Han Sun^e,i^, Kvido Stříšovský^b*^, and Adam Lange^a,j*^

**[a]** Claudia Bohg, Spyridon Kosteletos, Carl Öster, Taoran Xiao, Henry Sawczyc, Sascha Lange, Adam Lange*
Research Unit Molecular Biophysics
Leibniz Forschungsinstitut für Molekulare Pharmakologie (FMP)
Robert-Rössle-Straße 10, 13125 Berlin, Germany
*E-mail: [alange@fmp-berlin.de](mailto:alange@fmp-berlin.de)

**[b]** Yurii Dubanych, Kathrin Bach, Denise-Liù Gracias Leone, František Filandr, Kvido Stříšovský*
Institute of Organic Chemistry and Biochemistry
Czech Academy of Sciences
Flemingovo nám. 2, 160 00 Prague, Czech Republic

*E-mail: [kvido.strisovsky@uochb.cas.cz](mailto:kvido.strisovsky@uochb.cas.cz)

**[c]** Yurii Dubanych

Faculty of Food and Biochemical Technology

University of Chemistry and Technology

Technická 5, 166 28 Prague, Czech Republic

**[d]** Martin Neuenschwander, Andreas Oder, Carola Seyffarth, Jens Peter von Kries
Core Facility Screening Unit
Leibniz Forschungsinstitut für Molekulare Pharmakologie (FMP)
Robert-Rössle-Straße 10, 13125 Berlin, Germany

**[e]** Tillmann Utesch, Michael Lisurek, Han Sun
Research Unit Structural Chemistry & Computational Biophysics
Leibniz Forschungsinstitut für Molekulare Pharmakologie (FMP)
Robert-Rössle-Straße 10, 13125 Berlin, Germany

**[f]** Marc Wegert**,** Christian P. R. Hackenberger
Research Unit Biomolecule Modification and Delivery
Leibniz Forschungsinstitut für Molekulare Pharmakologie (FMP)
Robert-Rössle-Straße 10, 13125 Berlin, Germany

**[g]** Christian P. R. Hackenberger
Institute of Chemistry
Humboldt-Universität zu Berlin
Brook-Taylor-Straße 2, 12489 Berlin, Germany

**[h]** Edgar Specker
Core Facility Compound Management
Leibniz Forschungsinstitut für Molekulare Pharmakologie (FMP)
Robert-Rössle-Straße 10, 13125 Berlin, Germany

**[i]** Han Sun
Institute of Chemistry
Technische Universität Berlin
Straße des 17. Juni 135, 10623 Berlin, Germany

**[j]** Adam Lange
Institute of Biology
Humboldt-Universität zu Berlin
Invalidenstraße 42, 10115 Berlin, Germany

# Materials and Methods

## Expression & Purification of GlpG Samples

GlpG samples were prepared as described before ^[1]^. Briefly, the DNA coding region for FL GlpG and the GlpG core domain (i.e. GlpGΔN, residues 87-276) were amplified from the *E. coli* genome and cloned into a pET15b vector (Novagen). The recombinant GlpG core domain was overexpressed in *E. coli* Tuner (DE3) pLysS cells. FL GlpG was overexpressed in C41 (DE3). After induction with 500 µM Isopropyl β-D-1 thiogalactopyranoside (IPTG) for 15 hours at 25 °C, cells were harvested and resuspended in lysis buffer (50 mM Tris-HCl, 300 mM NaCl, 20% glycerol (v/v), 1.4 mM β-Mercaptoethanol, 10 mM imidazole, 0.2% (w/v) DM, pH 8.0) and lysed with 5-10 passages through a LM10 microfluidizer (Microfluidics, US) with 15,000 psi working pressure until the lysate was clarified. After lysis, the insoluble parts were removed by centrifugation at 20,000 x g for 20 min and 2% (w/v) n-decyl-β-maltoside (DM, GLYCON Biochemicals, Germany) was added to the supernatant. Solubilized GlpG was purified on a 5 ml HiTrap TALON crude Co^2+^ affinity column (GE Healthcare Life Sciences, US) using 8 column volumes (CV) of washing buffer (50 mM Tris-HCl, 300 mM NaCl, 20% glycerol (v/v), 1.4 mM β-Mercaptoethanol, 20 mM imidazole, 0.2% (w/v) DM, pH 7.5) and 5 CV of elution buffer (50 mM Tris-HCl, 300 mM NaCl, 20% glycerol (v/v), 1.4 mM β-Mercaptoethanol, 500 mM imidazole, 0.2% (w/v) DM, pH 7.5). Subsequently, the protein was desalted using a HiPrep™ 26/10 Desalting column (GE Healthcare, Germany) into 50 mM Tris and 150 mM NaCl, 5% Glycerol, 0.2% DM (w/v), pH 7.4 and flash-frozen in liquid nitrogen if not reconstituted immediately. Purified GlpG concentration was determined by a Bradford assay in the presence of 0.5% (w/v) α-cyclodextrin, and sample purity was confirmed by SDS-PAGE.

To produce the [^2^H, ^13^C, ^15^N]-labeled GlpG samples, ^15^NH_4_Cl and ^13^C_6_-D_7_-glucose (Cambridge Isotope Laboratories, USA) were used as the sole nitrogen and carbon sources in a perdeuterated M9 medium. Cells were adapted to D_2_O during the pre-induction growth phase using a stepwise adaptation protocol ^[2]^. During purification the samples were 100% H_2_O back-exchanged at labile, solvent-exposed sites ^[1,3]^.

## Reconstitution

For solid-state NMR measurements, the labeled rhomboid protease was reconstituted into *E. coli* total lipid extract liposomes. For this, deuterated *E. coli* total lipid extract (homemade) was dissolved in dialysis buffer (50 mM Tris, 150 mM NaCl, pH 7.4) containing 3% (w/v) DM and added to the purified GlpG sample at a lipid:protein ratio of ~30:1 (mol/mol). Detergent was removed by dialysis (Spectra/Por^®^ 1 dialysis tubing, MWCO 6000-8000 Dalton) against 100-fold dialysis buffer over the course of 10 days and with buffer exchanges every 2 days until the sample was completely turbid. To facilitate detergent removal, 0.5 g/L Bio-Beads SM-2 resin (Bio-rad) were added to the dialysis buffer.

## Solid phase peptide synthesis (SPPS)

Peptides were synthesized via an automated microwave-assisted synthesizer (CEM, US) by solid-phase peptide synthesis (SPPS) using a standard Fmoc/tert-butyl (t-Bu) protocol on TentaGel S Ram resin (RappPolymere, Germany).

Couplings were performed with 0.2 M Fmoc-amino acids (Fmoc-AA-OH), 0.25 M diisopropylcarbodiimide (DIC), and 0.25 M ethyl cyanohydroxyiminoacetate (Oxyma) in dimethylformamide (DMF). Fmoc deprotection was achieved with 20% piperidine in DMF. Acetylation was carried out overnight at room temperature using DMF, acetic anhydride, and diisopropylethylamine (DIPEA). Peptides were cleaved and deprotected from the resin by treatment with a cocktail of trifluoroacetic acid (TFA), phenol, water, methylphenylsulfide, and 1,2-ethanedithiol for 3 h at room temperature. The cleavage solution was collected, and crude peptides were precipitated from ice-cold tert-butyl methyl ether, followed by five washes with dry diethyl ether. Final purification and analysis were performed by reverse-phase HPLC on a Vydac C18 column (Hesperia, US) using a linear gradient from 5 to 60% acetonitrile containing 0.05% TFA. Peptide identities were confirmed by MALDI-TOF (microflex LT, Bruker Daltonics, Germany).

All solvents were purchased from Thermo Fisher Scientific (USA), and amino acids and coupling reagents from Iris Biotech (Germany).

## Fluorescent Activity Assessment of GlpG

The twin arginine transporter A (TatA), the natural substrate of AarA in *Providencia stuartii,* a homologous rhomboid protease to GlpG ^[4]^, was used as the substrate for the GlpG activity assay as described before ^[5]^. Briefly, a fluorescein isothiocyanate (FITC) - labeled peptide with the first 33 amino acids of TatA and a ß-alanine linker was produced by solid-phase peptide synthesis (FITC-_β_A-MESTIATAAFGSPWQLIIIALLIILIFGTKKLR). The peptide was dissolved in 50 mM Tris, 150 mM NaCl, 0.2% (w/v) DM, and 0.2% (w/v) N-Lauryl sarcosine to a final concentration of 400 μM.

*E. coli* total lipid extract (Avanti Polar Lipids, USA) was resuspended in 50 mM Tris and 150 mM NaCl to a final concentration of 10 mg/ml. Using 400 nm Nuclepore™ Track-Etched Polycarbonate filters (Whatman, USA) the lipids were extruded 31 times with an Avanti mini extruder (Avanti Polar Lipids, USA) to generate liposomes with a defined size.

4 pmol of DM solubilized GlpGΔN or FL were mixed with 20-fold molar excess of FITC-labeled TatA peptide in a solution of 1 mg/ml *E. coli* total liposomes in 50 mM NaOAc, 150 mM NaCl, 0.2% (w/v) DM, pH 4. The mixture was incubated for at least 20 min at room temperature. It was then diluted 20-fold with 12.5 mM NaOAc, and 37.5 mM NaCl, pH 4 to reduce the detergent below its critical micelle concentration. After incubation for 10 min, the proteoliposomes were separated from the detergent by ultracentrifugation at 186 000 x g for 1 h at room temperature. The samples were resuspended in neutral pH buffer (50 mM Tris, 150 mM NaCl, pH 7.4), immediately flash-frozen in liquid nitrogen and stored at -80°C until required.

For manual analyses and initial testing, fluorescence was measured every 5 min for 120 min at 25 °C (excitation 485 nm, emission 535 nm) using a FLUOstar Omega microplate reader (BMG LABTECH, Germany). Each experiment was conducted in three biological replicates, with three technical replicates each. RFU readings are shown per biological replicate, due to differing RFU scales.

## Activity assessment of GlpG with full-length substrate

FL TatA was used as the substrate for the GlpG activity assay as described before ^[1,3]^. Briefly a synthesized hexahistidine-SUMO-TatA-FLAG DNA sequence was cloned into a pET15b vector (Novagen). The recombinant substrate protein was overexpressed in a GlpG knock-out *E. coli* BL21(DE3) strain and purified and reconstituted as described for GlpG. 25 μL of substrate-containing proteoliposomes (∼5 mg/mL protein concentration, 30:1 (mol/mol) lipid/protein ratio) was mixed with 50 μL of protease-containing proteoliposomes (∼0.5 mg/mL protein concentration, 30:1 (mol/mol) lipid/protein ratio) in the presence of detergent DM (0.2% w/v) at 37 °C overnight. This corresponds to a molar ratio of 1:5:180 (GlpG:substrate:lipid). To verify the inhibitory effects of our compounds, 20x molar excess (50 µM) of compounds 1-23 of Table S1 were added. The reaction was stopped by the addition of SDS-PAGE loading buffer and observed via SDS-PAGE. Due to the highly negative charge of the protein, the hexahistidine-SUMO-TatA-FLAG tandem protein and its cleavage products are less mobile in the SDS-PAGE and run higher than the expected 24 kDa, 11 kDa, or 13 kDa.

## Activity Assessment of Chymotrypsin

α-Chymotrypsin was diluted to a concentration of 1 mg/ml in 10 mM Tris-HCl, pH 7.8. The EnzChek™ Protease Assay Kit (E6638, Invitrogen, US) was used for activity assessment according to the manufacturer's protocol. Briefly, 20x digest buffer was diluted to 1x concentration and lyophilized BODIPY-casein was diluted in phosphate-buffered saline (PBS). The chymotrypsin stock was dissolved in digest buffer to a final concentration of 10 µg/ml. BODIPY-casein was dissolved in digest buffer (BODIPY-casein:buffer 1:100). Chymotrypsin and BODIPY-casein were added in equal parts to a final volume of 10 µL to start the reaction, the fluorescence is read at an excitation of 485 nm and an emission of 535 nm for 120 min at 25 °C as described below (section 1.11).

## PARL Expression and Purification

PARL expression was performed according to the step-by-step protocol described in a previous study ^[6,7]^. In brief, the DNA coding region for truncated PARLΔ77 (i.e. residues 78-379) was cloned into a pPICZA vector, incorporating a C-terminal TEV cleavage site, GFP, and a hexahistidine-tag. This construct was transformed into competent G115S *P. pastoris* cells (Invitrogen, US). A high-expressing (with high GFP fluorescence) clone was selected and cultured overnight at 28 °C in 50 ml of buffered glycerol - complex medium (BMGY). Subsequently, 2 L of BMGY medium was subinoculated to initiate growth with a starting OD600 of 0.05, and the culture was grown approximately for 20 hours at 28 °C.

The cells were harvested by centrifugation, and the resulting cell pellets were resuspended in 4 L of buffered methanol - complex medium (BMMY) to reach an OD600 of 2 and to start the expression. Cultures were induced for 48 hours at 24 °C, with the addition of fresh methanol (final concentration of 1% (v/v)) every 24 hours. Following expression, the cells were harvested, resuspended in lysis buffer (50 mM Tris, 200 mM NaCl, 5% (v/v) glycerol, pH 8), supplemented with cOmplete™, EDTA-free Protease Inhibitor Cocktail (Roche, Switzerland), and DNase I from bovine pancreas (Sigma–Aldrich, US), and lysed by 10-12 passages through an LM10 microfluidizer (Microfluidics, US) at a working pressure of 20,000 psi.

Membranes were isolated by ultracentrifugation. The isolated membranes were homogenized with a Potter homogenizer in solubilization buffer (50 mM Tris-HCl, 200 mM NaCl, 20% (v/v) glycerol, 10 mM imidazole, and 1% (w/v) DDM, pH 8) followed by solubilization overnight at 4 °C. Insoluble material was pelleted by ultracentrifugation, and the supernatant was bound to TALON Superflow cobalt resin (GE Healthcare, US) for 1 hour at 4 °C. The protein-bound resin was washed with 4 CV of wash buffer (50 mM Tris-HCl, 300 mM NaCl, 20% (v/v) glycerol, 0.1% (w/v) DDM, 20 mM imidazole, pH 7.4) and subsequently eluted with 5 CV of elution buffer (50 mM Tris-HCl, 300 mM NaCl, 20% (v/v) glycerol, 0.1% (w/v) DDM, 300 mM imidazole, pH 7.4).

Dialysis (1:100 ratio, Spectra/Por^®^ 2 dialysis tubing, MWCO 12,000-14,000 Dalton) was performed for 2 hours to remove imidazole (50 mM Tris-HCl, pH 7.4, 200 mM NaCl, and 20% (v/v) glycerol). The purified PARL-GFP fusion protein was digested by incubation with an (in-house prepared) TEV protease and 1 mM TCEP overnight at 4 °C. PARL was subsequently purified from GFP and TEV using His60 Ni Superflow Resin™ (Takara Bio Group, Japan) and dialyzed again, both as described above. The flow-through was collected and concentrated using a 10,000 Da MWCO concentrator (Sartorius, Germany). The purified protein, with a concentration of 1 mg/ml, as measured by using a Bradford assay, was flash-frozen and stored at -80 °C until further use.

## Activity Assessment of PARL

To test PARL activity, the procedure described for GlpG (see Section 1.4) was followed with PINK1-FITC replacing TatA-FITC. The PINK1-FITC peptide, FITC-βA-AVFLAFGLGLG ^[8]^, was prepared via solid-phase peptide synthesis and includes a segment of the PINK1 transmembrane domain.

## Synthesis of a pentapeptidyl chloromethyl ketone (CMK) inhibitor

As a positive control for our experiments, we utilized a pentapeptidyl chloromethyl ketone (CMK) inhibitor with the sequence Acetyl-RVRHA-cmk, synthesized according to a previously published protocol ^[9,10]^.

## Compound Library

A library of 68,288 compounds was used at the Screening Unit core facility of the FMP. The library consists of a 30,976 member diversity set that was designed based on the maximum common substructure principle^[11]^, 704 compounds derived from the ChEMBL database, 4576 fragments, a set of 1280 compounds with known pharmacological activity (Library of Pharmacologically Active Compounds, LOPAC, Sigma–Aldrich, US), 1536 FDA-approved drugs or drug candidates (Selleckchem, Germany), 20,064 synthetic compounds based on natural product classes (AnalytiCon Discovery, Germany) and a further set of 9152 compounds donated from academic groups.

The screening libraries are arranged in 384-well plate format, in which compounds are plated into columns 1 to 22. The compounds are dissolved in DMSO at a concentration of 10 mM. DMSO alone is plated into columns 23 and 24 to serve as controls. In that way, 352 compounds were screened per plate with 32 controls. GlpGΔN was used for the full library, FL GlpG, PARL and chymotrypsin were only used during the IC_50_ screening.

## High Throughput Screening (Primary Screen) for Small Molecule Activators and Inhibitors

All assays were performed at 22 °C in a white 384 well plate (784075, small volume, hibase, medium binding, Greiner, Austria) in a final reaction volume of 10 µl, using a total of 198 assay plates. Columns 1-22 were used for the compound screening, while column 23 was left for positive control reactions and column 24 for negative control reactions.

For the main screen of GlpGΔN, 5 µl of reaction buffer (50 mM Tris, 150 mM NaCl, pH 7.4) was pipetted in each well using a MicroFlo dispenser (Biotek, US). Every inhibitor was transferred to each well of columns 1-22 using a Beckman FX workstation (Beckman Coulter, Germany) equipped with an FP1 pin tool (V&P Scientific, US). The average transferred compound volume was ~25 nL according to color tests, yielding a final compound concentration of 25 µM and a DMSO concentration of 0.25% (v/v). The flash-frozen enzyme-substrate-liposome mixture was thawed and 5 µl of each reaction was immediately pipetted to columns 1-23, the negative control, containing buffer instead of GlpGΔN, was pipetted in column 24. The fluorescence was read using a SafireII microplate reader with Magellan software (Tecan, Switzerland) at an excitation wavelength of 485 nm and an emission wavelength of 535 nm. This first readout provided the starting fluorescence value, which was used to remove strongly autofluorescent compounds. The plates were then incubated for 90 min in a humidity chamber and the fluorescence was read a second time using the same conditions. This second readout provided the end fluorescence value, which was used to detect enzyme activity.

## Confirmatory Screen and IC_50_ Validation for GlpGΔN

Through initial testing, a total of 678 activators and 1850 inhibitors were found. For the selection of activators, samples that showed a Z-score greater than 4 and a percent activity increase of at least 10% were selected, providing 631 hits. For the selection of inhibitors, samples that showed a Z-score less than -4 and a percent activity decrease of at least 10% were selected, providing 1116 hits. 1056 samples with the lowest and highest Z-scores were selected for further investigations. After the confirmatory screening, 326 compounds provided a 20% change in fluorescent intensity compared to the positive control. Those were chosen for the IC_50_ screening.

To obtain IC_50_ data for GlpGΔN, a Tecan Evo workstation equipped with a 384-channel fixed tip pipetting head (Tecan, Switzerland) was used. 1:2 serial dilutions in DMSO were generated across plates, to get 9 serial dilutions ranging from 10 to 0.04 mM. The 10 mM compound stock plate was transferred 2x using the FP1 Pin tool to create the highest assay concentration of 50 µM, then all predilutions were transferred 1x using the FP1 Pin tool to create the 1:2 dilution series in assay plates starting with 50 µM compound in 10 µl assay volume.

## Dilution Experiments and Inhibition kinetics

Reversibility of inhibition by compounds **2**, S**22**, S**24**, **4**, and S**42** was evaluated as described before^[9]^ with some modifications. Briefly, FL GlpG (0.4 µM) was pre-incubated with each compound at 50 µM (~10× IC_50_) and then diluted 100-fold into reaction buffer containing either 0.5 µM (~0.1× IC_50_) or 50 µM inhibitor, together with a 10 µM fluorogenic substrate^[12]^.

The mode of inhibition by compound S**22** was determined as described before^[9]^ with some modifications. Initial reaction rates of a fluorogenic peptide substrate^[12]^ cleavage by FL GlpG (50 nM) were measured across substrate concentrations up to 6-fold over K_M_ (~100 µM) and at several inhibitor concentrations. The data were globally fitted to the models of competitive, non-competitive, uncompetitive, and mixed inhibition in GraphPad Prism 10.6.1 software. The fits were statistically analysed using F test and AICc test.

## Mechanistic Characterization by Mass spectrometry

The formation of covalent adducts of compounds **4**, **2**, S**22**, and S**24** with GlpG was analysed using denaturing electrospray ionization (ESI) mass spectrometry. The FL GlpG (6 µM) or its S201T mutant was pre-incubated with 50 µM inhibitor at 25 °C for 1 h and then directly injected into a LC-MS system consisting of an ACQUITY UPLC I-Class LC system with MassPrep Micro Desalting Column (Waters, USA) and Synapt G2 mass spectrometer set to ToF-MS sensitivity mode without ion mobility. The sample was first trapped and desalted for 30 seconds at 400 µl/min flowrate, then eluted using a 13-minute gradient 5% to 80% B at 100 µl/min flowrate (mobile phase A: 0.1% FA in H_2_O, mobile phase B: 0.1% FA in acetonitrile). ESI source was tuned for low ion activation to prevent fragmentation of protein and any covalent modifications, with the following source parameters: ESI Capillary Voltage: 3000 V, Source Temperature: 120 °C, Desolvation Temp.: 550 °C, Desolvation Gas: 800 L/hour, Sampling Cone: 20 V, Extraction Cone: 4.5 V, Trap Collision Energy: 4 V. Data was processed in UniDec software version 7.0.2, with main processing parameters as follows: Gaussian smoothing: 3.0, Charge range: 10 to 40, mass range: 5000 to 50000, and FWHM of 0.35. For a competitive experiment, FL GlpG was first incubated at 25 °C for 45 min with a 50 µM ketoamide inhibitor STS1526 (compound **5** ^[12]^), then 50 µM S**22** was added, and the mixture was incubated for another 15 min and analysed.

## Inhibition of GlpG in vivo

The inhibition assay of compounds **4**, **2**, S**22**, and S**24** on endogenous GlpG was evaluated using E. coli NR698 (genetically permeabilised outer membrane) or glpG::tet derivative strain (wild-type) as described before^[9]^ with minor modifications. Briefly, single colonies were inoculated into LB-Kan and grown to OD_600_ = 0.6 at 37 °C. Cells were incubated with a 2-fold dilution series (124 nM to 126,582 nM) of inhibitor or DMSO control for 15 min at room temperature, followed by induction of LacY TM2 substrate expression with 1 mM L-rhamnose. After 4 h of growth at 37 °C with shaking, cells were lysed, and substrate cleavage was analyzed by Western blot using an anti-FLAG antibody.

## IC_50_ Validation for FL GlpG, Chymotrypsin, and PARL

An IC_50_ validation screen was done for FL GlpG, Chymotrypsin, and PARL. Using a Biomek i7 workstation equipped with a disposable tip 384-channel head (Beckman Coulter, Germany), the residuals of cherry-picked compounds were transferred into a LP-0200 low-dead volume ECHO plate (5 mM and 0.156 mM predilution). From these 2 mother plates, a serial dilution series in assay-ready plates was generated by transferring the following volumes using an ECHO 650 acoustic dispenser (Beckman Coulter, Germany): 100, 50, 25, 12.5, 6.25 nL of 5 mM and 100, 50, 25, 12.5 nL of 0.156 mM. This dilution procedure created a 1:2-fold dilution series, starting with 50 µM compound in 10 µl assay volume.

10 µl of Bodipy/Chymotrypsin mixture were dispensed into columns 1-23 of a Greiner low-volume assay-ready plate (#78075) using a MicroFlo dispenser. 10 µL of BODIPY-casein in digest buffer was added using a manual multipipette to column 24.

Plates were placed in an EnVision plate reader (PerkinElmer) with plate stackers and measured every 15 min (Ex 485 nm/Em 535 nm) for 120 min. The slopes of the fluorescence increase were used as the statistically relevant readout. Measuring slopes provided more informative data compared to only capturing the start and end fluorescence, as done in the primary screening and initial IC_50_ validation.

## Analysis of Screening Results

Data were normalized for each plate using statistically robust estimators (Equation 1) ^[13]^.

$$\left( 1 \right) Z score=\frac{x_{i}-Median}{MAD\times1.48258}$$

The $Z score$ indicates how many standard deviations an observation is above or below the mean. $x_{i}$ represents the signal of a single sample, $Median$ is the median signal on a plate without the controls, and $MAD$ is the median absolute deviation on a plate without the controls.

$$\left( 2 \right) Percent activity= 100\%*\frac{x_{i}-Neg}{\left| Pos-Neg \right|}$$

$Percent activity$ (Equation 2) is the response relative to an unperturbed state. $Neg$ is the median of the negative control samples (no enzyme activity), and $Pos$ is the median of the positive control samples (=100% enzyme activity) per plate.

$$\left( 3 \right) Z^{'}=1- \frac{3 \times\left( \delta_{P}+\delta_{N} \right)}{\left| \mu_{P}-\mu_{N} \right|}$$

$Z^{'}$ is a common statistical tool to measure the effective dynamic signal range of HTS assays and serves as a quality control metric ^[14]^. $\delta_{P}$ and $\delta_{N}$ are the standard deviations of the positive and negative controls, respectively, and $\boldsymbol{\mu}_{\boldsymbol{P}}$and $\boldsymbol{\mu}_{\boldsymbol{N}}$ are the mean values of the positive and negative controls of a plate (Equation 3).

$$\left( 4 \right) f\left( x \right)=c+ \frac{d-c}{1+exp\left[ b\left( logx-loge \right) \right]}$$

IC_50_ determination was carried out by using the four-parameter log-logistic function (Equation 4), and the “drc” R-package for determining dose/response curves ^[15]^ ($b$: Hill coefficient (steepness of the IC_50_ curve at the inflection point). $e$: IC_50_ value. $c$ and $d$: left and right activity asymptotes). The activity data were determined in a three-step process: beginning with a curve fit and using a robust estimation of fitting errors to find an initial IC_50_ model, followed by automated removal of outliers, and final refitting with the use of standard estimation of fitting errors ^[16]^.

The data were preprocessed, including initial graphical quality control and data normalization, using in-house software. Reports containing chemical structures were generated using KNIME ^[17]^ and graphics were prepared with the R-statistics framework ^[18]^. **Figure 2** was generated using a custom Python script with Matplotlib and Pandas libraries. Tables S1 and S3 were prepared using LaTeX for formatting and RDKit for generating and processing chemical structures.

## IC_50_ Reevaluation of Final Compound Selection

The 6 best compounds were chosen: (**1** / 100940, **2** / 205640, **3** / 207927, **4** / 208274, **5** / 300541, and **6** / 710980). FL GlpG was produced as previously described^[19]^. The IC_50_ test was conducted with GlpG digesting the substrate KSp63. The structure of KSp63 is as follows:


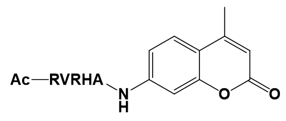


Briefly, inhibition assays were performed in 50 mM potassium phosphate, 150 mM NaCl, 20% (v/v) glycerol, 0.05% (w/v) PEG8000, 0.05% (w/v) DDM, and 16% (v/v) DMSO, pH 7.4 for compounds **2** (ChemDiv Inc., US), **5** (ChemDiv, Inc, US), and **6** (Analyticon, Germany), and 10% (v/v) DMSO for compounds **1** (ChemDiv Inc., US), **3** (ChemBridge Corporation, US), and **4** (Life Chemicals Inc., Canada). GlpG was used at a final concentration of 400 nM, and the soluble substrate was at a final concentration of 100 µM. GlpG was incubated with different concentrations of the compounds at 37 °C for 15 min before adding the substrate. Measurements for compounds **1**, **3**, and **4** were conducted in triplicate, for **5** and **6** in duplicate, and for **2** once.

For compounds **1** and **4**, concentrations ranged from 100 µM-48.8 nM, 400 µM-48.8 nM and 400 µM-195 nM. For compound **3**, concentrations from 100 µM-48.8 nM, 400 µM-48.8 nM and 200 µM-97.5 nM were used. For compound **2**, concentrations from 250 µM-256 pM were used. For compounds **5** and **6** concentrations from 8 mM-2.56 µM and 4.8 mM-2.18 µM were used. Different concentration ranges were used due to varying compound solubilities.

## Docking of Final Compound Selection to GlpG and PARL

Compound docking was conducted following the procedure outlined previously ^[3]^. Briefly, the structures of GlpGΔN in both open and closed states were extracted from the crystallographic PDB structure 2NRF and preprocessed with PRIME ^[20]^. In this structure, chain A represents GlpG in an open state, while chain B represents the closed state of the protease. Both chains underwent preparation using the "Protein Preparation" tool in the Schrödinger suite 2022.3.

The compounds were prepared with LigPrep. Subsequent molecular docking was carried out with Glide ^[21]^ in the XP precession mode using a receptor grid of 20 Å centered around the active site S201.

To investigate the binding mode of compound **4** to PARL, we used AlphaFold3 to simultaneously predict the protein–ligand complex structure. The docking was performed using a locally installed Alphafold3 inference pipeline. As input, we provided the SMILES code of compound **4** and the amino acid sequence of human PARL (UniProt ID: Q9H300), which yielded two plausible poses ^[22,23]^. We selected the pose that was positioned closer to the catalytic site and more embedded within the protein, away from the expected membrane-facing surface.

Molecular graphics and analyses were performed with UCSF ChimeraX, developed by the Resource for Biocomputing, Visualization, and Informatics at the University of California, San Francisco, with support from National Institutes of Health R01-GM129325 and the Office of Cyber Infrastructure and Computational Biology, National Institute of Allergy and Infectious Diseases ^[24–26]^. Marvin was used for drawing, displaying and characterizing chemical structures, substructures and reactions, Marvin 23.17.0, Chemaxon (<https://www.chemaxon.com>).

## Docking of Structural Analogues

The covalent binding modes of the o-nitro, p-nitro, and o,p-nitro fragment ligands in FL GlpG were modeled for both the open (chain A) and closed (chain B) conformations (PDB ID: 2NRF)^[27]^ using the docking program GOLD (version 2021.2.0; Cambridge Crystallographic Data Centre)^[28]^ , as implemented in Discovery Studio (BIOVIA). Docking was performed using the ChemPLP scoring function^[29]^ . The binding site was defined based on receptor cavities identified in the X-ray structure of GlpG. Covalent docking was carried out by applying a restraint on the ester bond formed between the ligand and the catalytic residue Ser201 of GlpG.

## Treatment of solid-state NMR samples with Inhibitors

Compound **2** (ChemDiv Inc., US) and **4** (Life Chemicals Inc., Canada) were dissolved in dimethyl sulfoxide (DMSO) to a 100 mM stock solution. They were added to FL GlpG after dialysis (when the protein was in a lipid environment) at an inhibitor:protein molar ratio of 10:1. The final DMSO concentration was less than 0.2 % (v/v). The GlpG/compound mixture was incubated for 1 h. Samples were centrifuged for 2 h at 300,000 x g and packed into 1.9 mm rotors (Bruker, USA) with a few crystals of sodium trimethylsilylpropanesulfonate (DSS).

## Solid-state NMR analysis of Compounds 2, 4, and CMK bound to FL GlpG

Proton-detected solid-state NMR (ssNMR) experiments for the FL GlpG/CMK sample were conducted using a Bruker 900 MHz standard-bore spectrometer, equipped with a four-channel (^1^H, ^2^H, ^13^C, ^15^N) 1.9 mm probe, operating at 40 kHz MAS. To facilitate chemical shift assignments, 3D experiments (hCANH, hCONH, hCAcoNH, and hCOcaNH) were acquired following a previously published protocol ^[2]^. All spectra were recorded with 35% non-uniform sampling (NUS) with sampling schedules generated from http://gwagner.med.harvard.edu/intranet/hmsIST/ ^[30,31]^, reconstructed with the IRLS algorithm in the qMDD software ^[32–34]^, and processed with NMRPipe ^[35]^. For the reference apo FL GlpG sample (unbound state), a single hCANH spectrum was recorded on a Bruker 900 MHz spectrometer, equipped with a four-channel (^1^H, ^2^H, ^13^C, ^15^N) 1.9 mm probe, operating at 40 kHz MAS. Reconstruction and processing were done as described above.

Experiments for the FL GlpG/Compound **4** and **2** samples and their reference were conducted on a Bruker 800 MHz wide-bore spectrometer, equipped with a three-channel (^1^H, ^13^C, ^15^N) 1.9 mm probe, operating at 40 kHz MAS. The ligand-bound enzymes were analyzed using sensitivity-enhanced 3D experiments (hCANH and hCONH) with Simplified Preservation of Equivalent Pathways Spectroscopy (SPEPS) for C-N magnetization transfers ^[36]^, while for the ligand-free enzyme, a single 3D hCANH experiment was performed.

To systematically assess the magnitude of chemical shift changes, we categorized residues based on the deviation of their chemical shift changes relative to the mean shift difference between the reference sample and the inhibitor-bound samples. Each chemical shift difference was compared with the mean chemical shift difference, and significance thresholds were set at the mean + n × standard deviation, where n = 1, 2, or 3 represents weak, moderate, and strong differences, respectively (Table S9).

During all measurements, the sample temperature was maintained at approximately 20 °C, determined from the chemical shift of water relative to the sodium trimethylsilylpropanesulfonate (DSS) peak. D_2_O was used for experiments on the 900 MHz spectrometer. TopSpin 4.4.0 (Bruker) was used for ssNMR data processing. Data analysis were carried out using CCPNMR AnalysisAssign Version 3 ^[37]^ and custom Python scripts for the calculation and visualization of absolute chemical shift differences, average chemical shift differences, and standard deviations. The Python analysis utilized the following libraries: Pandas, NumPy, and Matplotlib.

## Biosimilarity search for Compounds 2 and 4

The similarity searches for compounds **2** and **4** were performed against an in-house downloaded PubChem Database (January 2024) in the Pipeline Pilot Software (BIOVIA Pipeline Pilot, Release 2018, San Diego, Dassault Systèmes) using FCFP_4 fingerprints ^[38]^.

We identified a total of 166 (compound **2**) and 13 (compound **4**) commercially available compounds with a similarity score ≥ 0.7. From these, the 10 most similar compounds to compound **2** (S**21**–S**30**) and the 9 most similar compounds to compound **4** (S**41**–S**49**) were purchased.

The selected compounds were procured from ChemBridge Corporation (S**22**, S**41**), ChemDiv, Inc. (S**23**, S**29**, S**42**), Vitas-M Chemical Limited (S**21**, S**24**, S**25**, S**26**, S**27**, S**28**, S**30**, S**43**) and Life Chemicals Inc. (S**44**–S**49**). Detailed information on PubChem IDs, similarity scores, and structures is provided in **Table S2**.

Each compound was tested at 10 µM and 50 µM for its effect on FL GlpG, as described in section 1.4. Subsequently, an IC_50_ determination was performed to evaluate the potency of the compounds, with particular focus on compounds S**22**, S**24**, S**40**, and S**41**. 7-amino-4-chloro-3-methoxy-1*H*-2-benzopyran (JLK6, Tocris Bioscience, UK) ^[39]^ was used as a reference compound. The compounds were diluted in an 11-point, 2-fold serial dilution ranging from 100 µM to 97.6 nM. Initial measurements were performed twice using a 2-fold dilution series ranging from 50 µM to 390 nM. Analysis was conducted as outlined in section 1.17 using a custom Python script with the following libraries: Pandas, NumPy, Matplotlib, and Scipy.

## TAMRA-FP assay

For the labelling of the active site of GlpG, ActivX™ TAMRA-FP Serine Hydrolase Probe (Thermo Fisher Scientific, Germany) was used as described before ^[40,41]^. Briefly, 0.5 µg of purified GlpG was mixed with compounds **2**, S**22**, S**24**, **4**, and DMSO to a final concentration of 50 µM and incubated for 20 min. Then the reactive probe was added to a final concentration of 0.5 μM. Subsequently, it was incubated for 1 h at 37°C protected from light. The reaction was stopped with 4x Laemmli buffer and subjected to SDS-PAGE. The gel was visualized with UV light and afterwards stained with Coomassie Brilliant Blue dye.

The same procedure was carried out for soluble serine proteases: trypsin from bovine pancreas (Sigma-Aldrich, US), α-chymotrypsin from bovine pancreas (Sigma-Aldrich, US), Proteinase K from *Tritirachium album* (Carl Roth, Germany), Elastase from porcine pancreas (Promega, US), subtilisin from *Bacillus licheniformis* (Sigma-Aldrich, US).

**Supplementary Figures:**


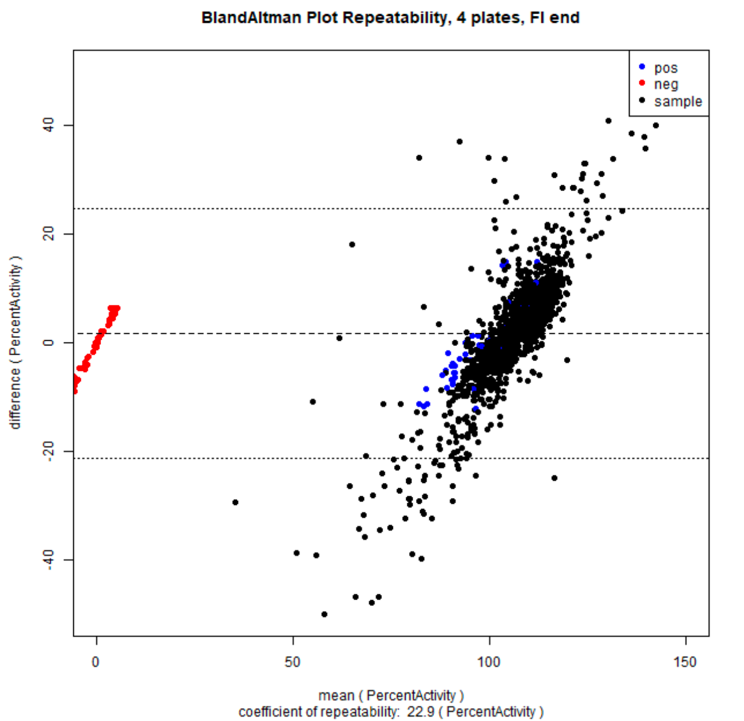


**Figure S1.** Bland-Altman Diagram of the initial screen test. The black dots illustrate the percentage activities resulting from two technical replicate measurements on GlpGΔN with 1408 compounds. Blue dots indicate the positive controls (no compound), while red dots represent the negative controls (no enzyme). The dotted lines, representing the limits of agreement, indicate the range within which approximately 95% of the differences between the two experiments are expected to fall (95% of measured values within ±22.9% activity).


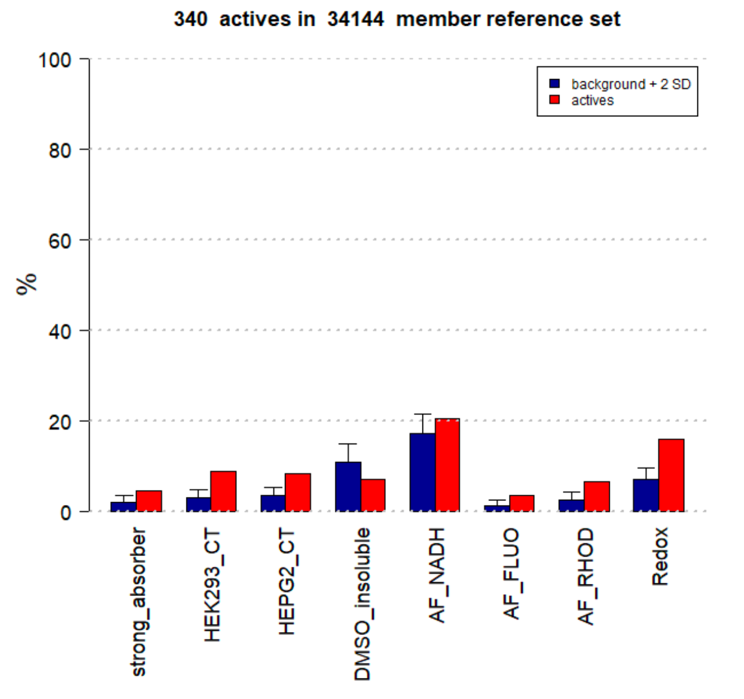


**Figure S2.** Analysis of potentially undesirable properties among inhibitory compounds identified in the screen. Red bars indicate the percentage of primary actives (inhibitory compounds before confirmatory screening) exhibiting a given property, while blue bars represent the occurrence of the same property in 50 randomly selected control samples, each matched in size to the set of primary actives. Properties assessed include strong absorbance, cytotoxicity (CT) in HEK293 or HEPG2 cells, DMSO insolubility, autofluorescence in NADH (AF_NADH), fluorescein (AF_FLUO), and rhodamine (AF_RHOD) channels, and redox activity.


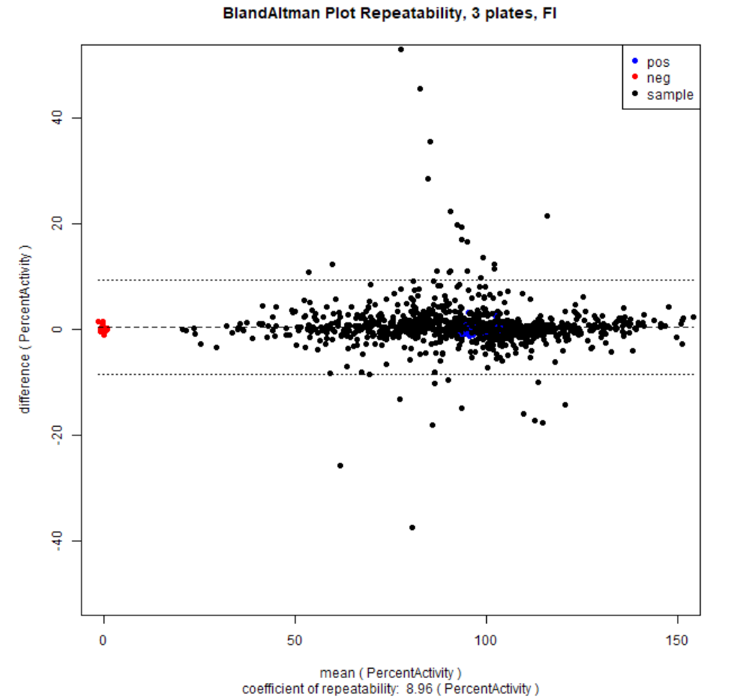


**Figure S3.** Bland-Altman Diagram for the confirmatory screen. The black dots represent the percentage activities resulting from two technical replicate measurements on GlpGΔN with 1056 compounds. The positive controls are indicated in blue, while the negative controls are shown in red. The dotted lines, representing the limits of agreement, indicate the range within which approximately 95% of the differences between the two experiments are expected to fall (95% of measured values within ±8.96% activity).


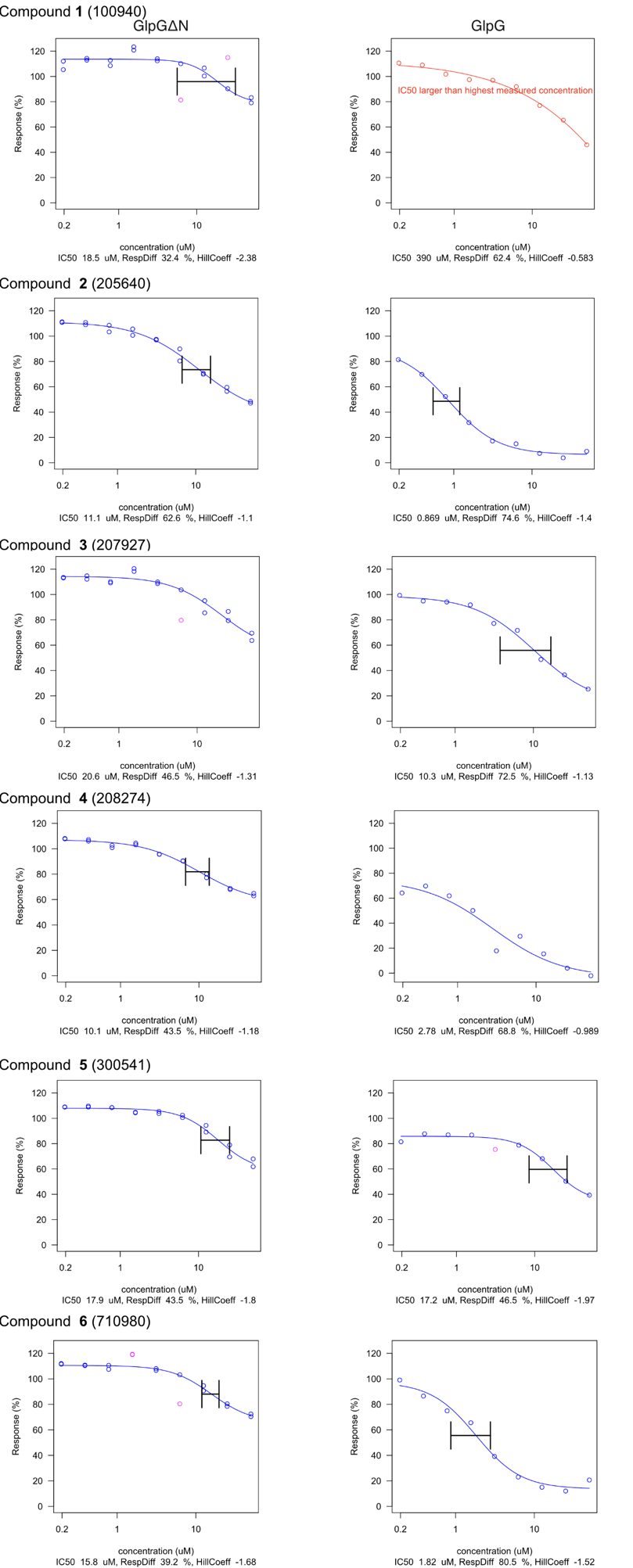


**Figure S4.** IC_50_ graphs depicting the inhibitory activity of the six finally selected compounds on GlpGΔN (left) and GlpG FL (right). IC_50_ = half-maximal inhibitory concentration, RespDiff = response difference, HillCoeff = Hill coefficient.


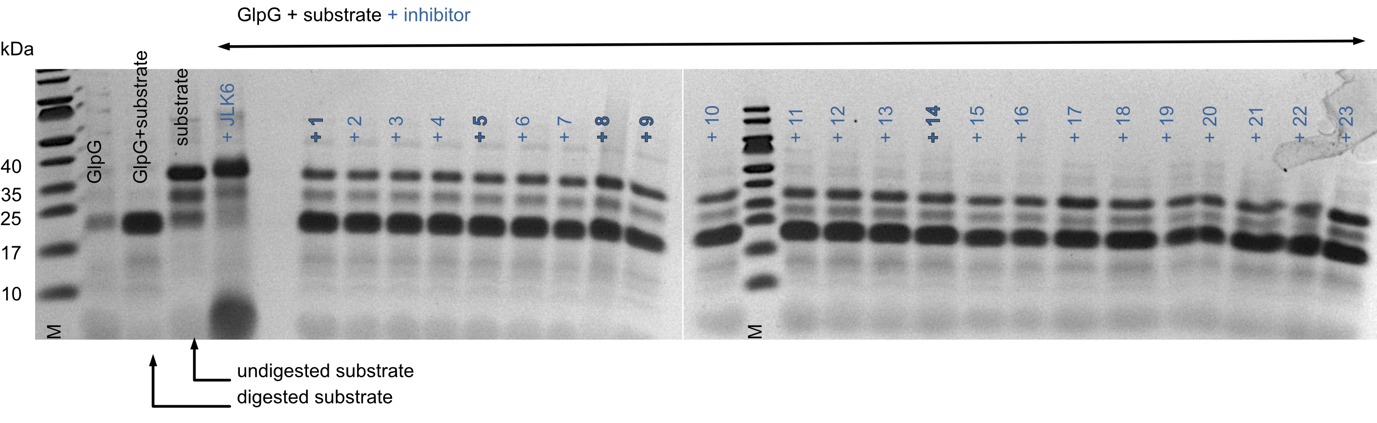


**Figure S5.** SDS-PAGE of 24 selected compounds active on FL GlpG. GlpG is digesting TatA-SUMO, as described before. Digested and undigested substrates exhibit different molecular weights. In the first lane, only GlpG is present; in the second lane, GlpG + TatA-SUMO; in the third lane, only TatA-SUMO; and in the fourth lane, GlpGΔN + TatA-SUMO with JLK6 inhibitor (50 µM final concentration). All subsequent lanes show GlpG + TatA-SUMO treated with 23 final active compounds (50 µM final concentration, **Table S1**). The six chosen inhibitors from the final selection are highlighted in bold. Note that the numbering is different from the main text. E.g. final selection inhibitor **4** corresponds to in-house library number 208274 and is the 9^th^ out of 23 selected compounds analyzed in this plot:

**1**: 100940, 2: 202960, 3: 203204, 4: 204534, **5**: 205640, 6: 206528, 7: 207735, **8**: 207927, **9**: 208274, 10: 209877, 11: 213917, 12: 216407, 13: 216420, **14**: 300541, 15: 405412, 16: 405877, 17: 406929, 18: 505617, 19: 506257, 20: 507994, 21: 708587, 22: 709732, 23: 710132

1=compound **1**, 5= compound **2**, 8=compound **3**, 9=compound **4**, 14=compound **5**


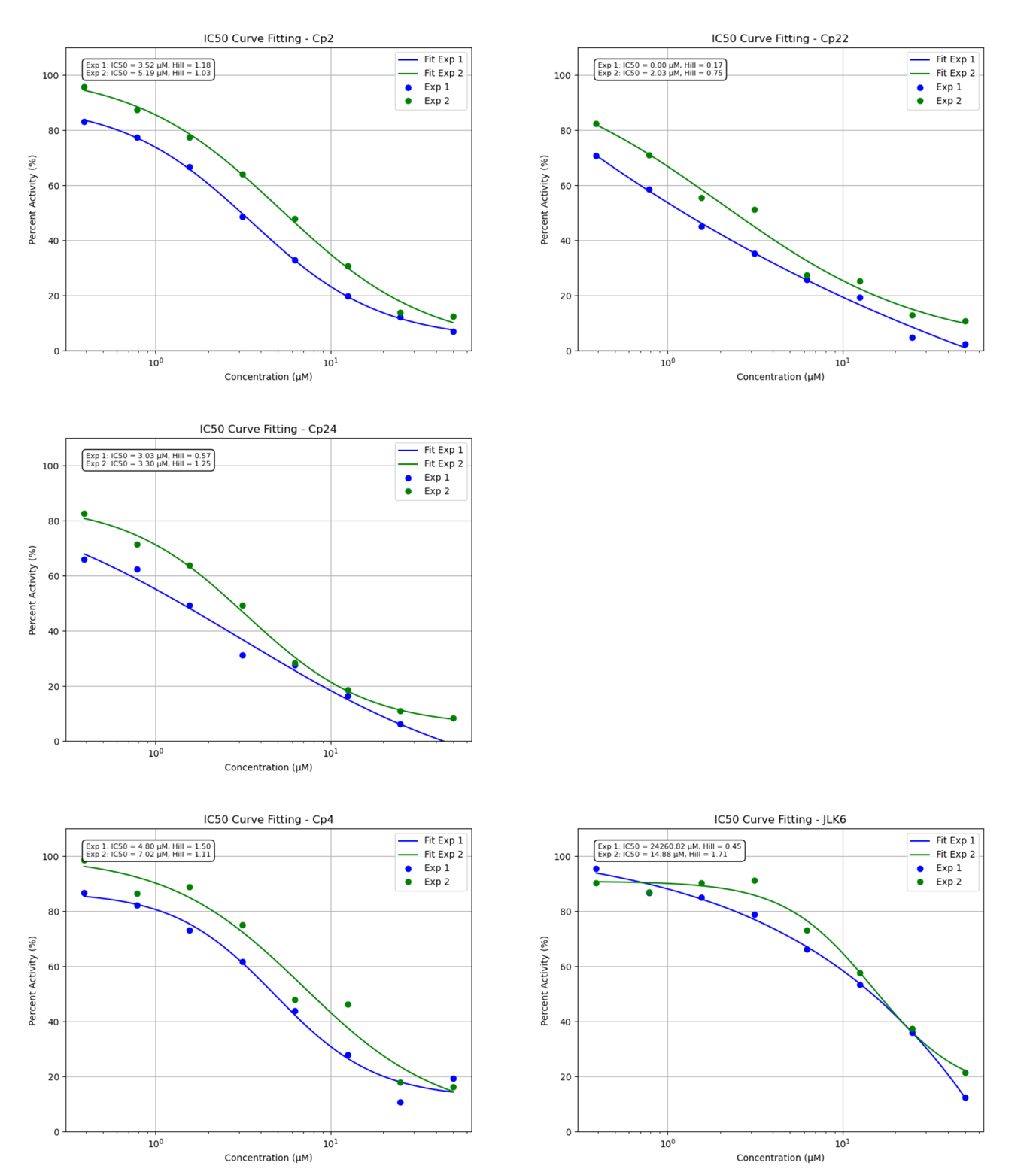


**Figure S6.** Dose–response curves (percent protease activity vs. inhibitor concentration) for full-length GlpG incubated with compounds **2**, **4**, S**22**, S**24**, and JLK6. For each compound, the calculated IC_50_ and Hill slope are shown. These data represent two biological replicates (blue, green) corresponding to the experiments in Figure 4 and Figure S7. Note, that in Figures 4 and S7, a broader concentration range was tested, which resulted in improved curve fitting and more robust parameter estimation. Note also, that in experiment 1 for S**22** and experiment 1 for JLK6, a reliable IC₅₀ value could not be determined, as the dose–response curve did not reach a well-defined midpoint within the tested concentration range.


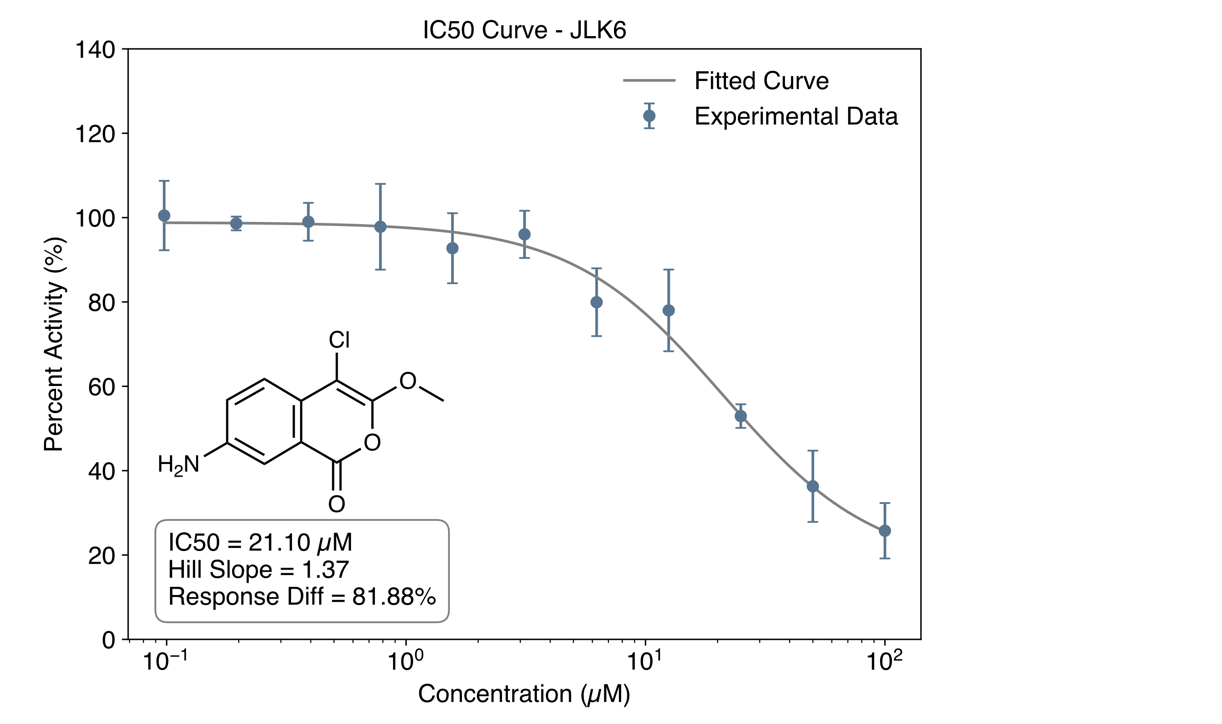


**Figure S7.** Dose-response curves (percent protease activity vs. compound concentration) for FL GlpG incubated with JLK6, showing IC_50_, Hill slope, and response difference (Diff.) as well as the chemical structure of JLK6. The experiments had 4 technical replicates, error bars indicate median absolute deviation (MAD).


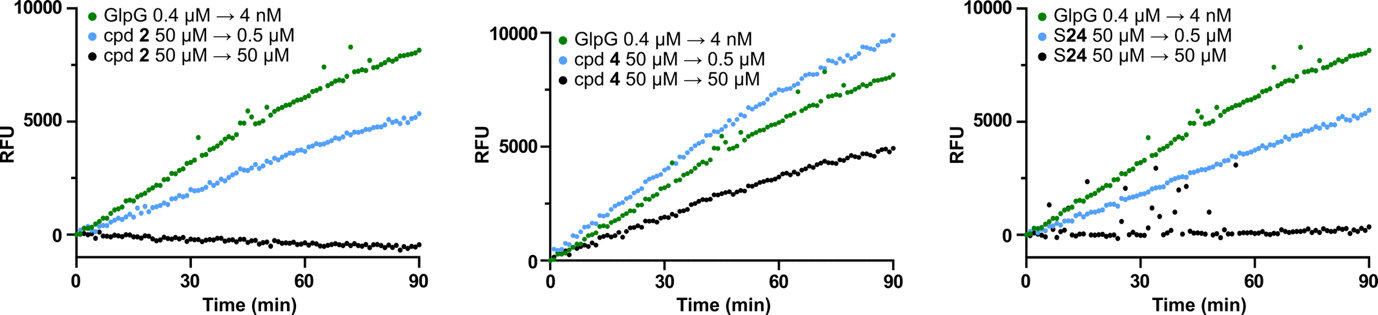


**Figure S8.** Reversibility analysis of compounds **2**, **4**, and S**24** in a dilution experiment. FL GlpG (0.4 µM) was pre-incubated with each compound at 50 µM (~10× IC50) and then diluted 100-fold into reaction buffer containing either 0.5 µM (~0.1× IC50) or 50 µM inhibitor, together with a 10 µM fluorogenic substrate^[12]^. The linear time courses in the presence of inhibitor indicate that compounds are reversible with fast-binding kinetics.


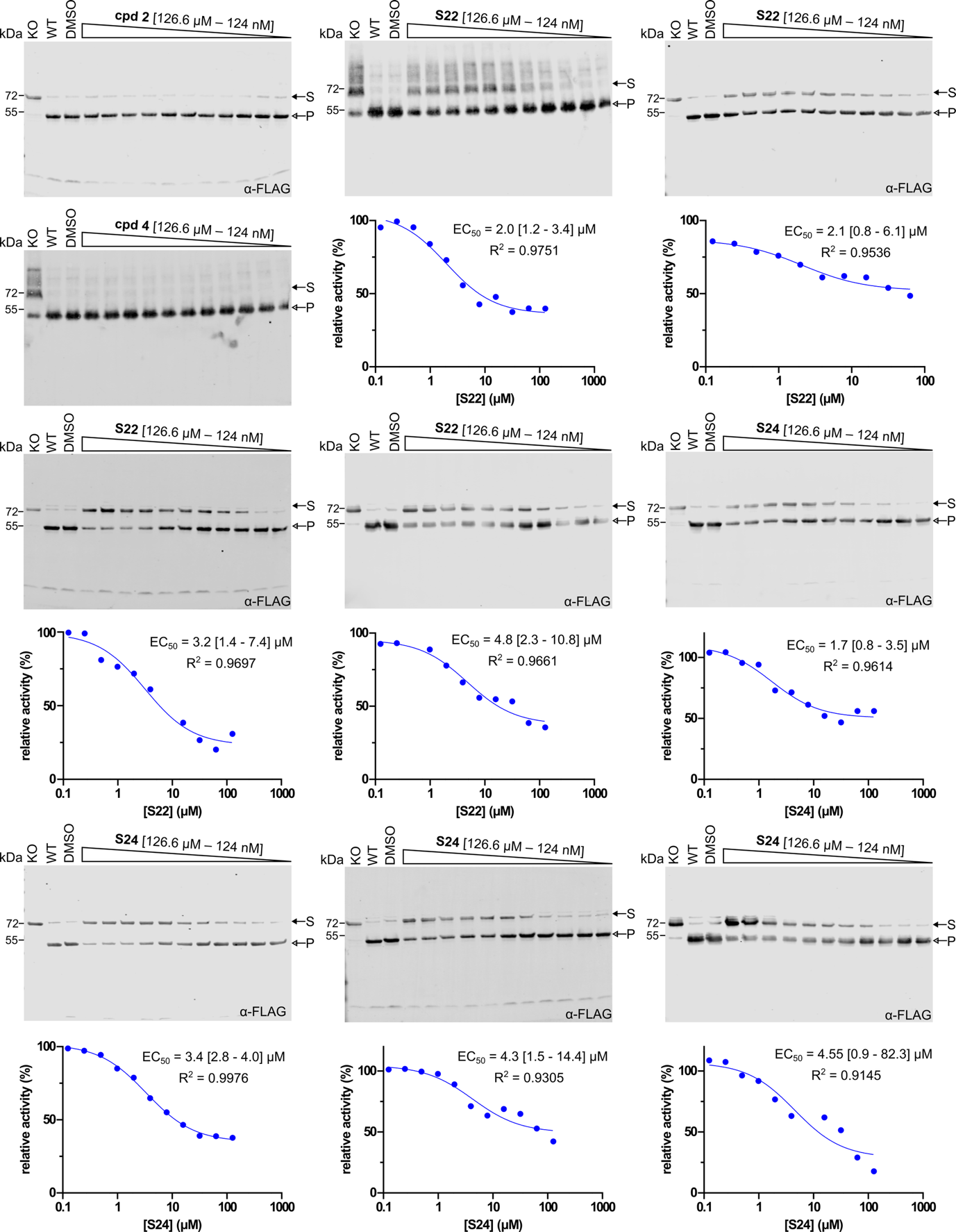


**Figure S9.** Inhibition assay *in vivo* on endogenous GlpG*.* Cleavage of model substrate MBP-LacYTM2-Trx by endogenous FL GlpG was followed by immunoblotting at a range of inhibitor concentrations. The EC_50_ curves were derived by densitometry based on quantitative, near-infrared fluorescence detection of the substrate (S) and product (P) bands as described previously^[9]^. Compounds **2** and **4** showed no detectable activity. The EC_50_ values for S**22** and S**24** were calculated as averages ± standard deviation from four independent experiments.


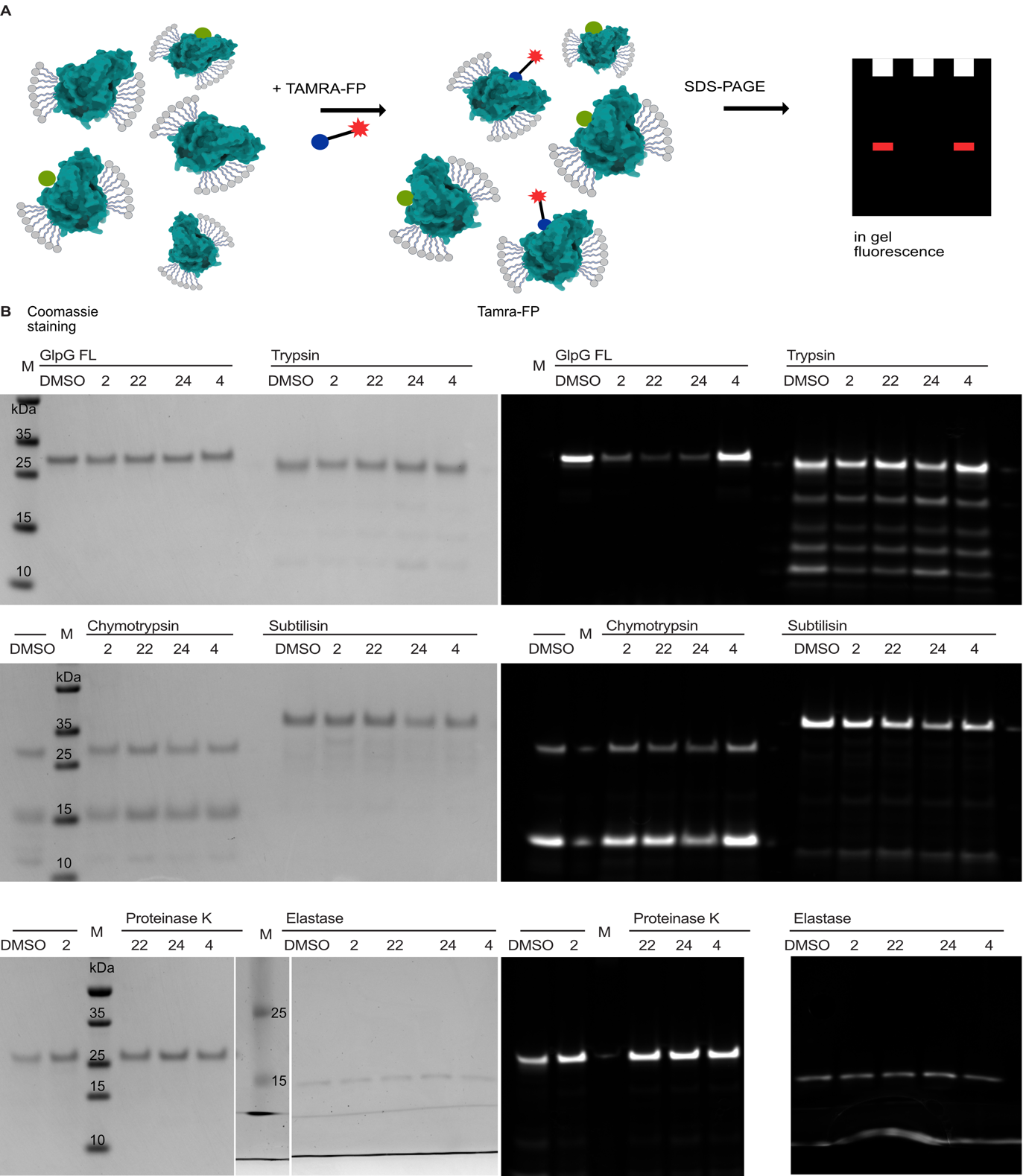


**Figure S10.** TAMRA-FP assay of serine proteases. **(A)** Schematic of the assay principle. In the absence of inhibitor (green), GlpG (turquoise) in detergent binds the fluorophosphonate probe TAMRA-FP (red and blue), which results in fluorescent labeling detectable in-gel. **(B)** TAMRA-FP labeling of GlpG and soluble serine proteases in the presence of inhibitors. GlpG, trypsin, chymotrypsin, subtilisin, proteinase K, and elastase were incubated with 50 µM of compounds **2**, S**22**, S**24**, **4**, or DMSO as control. Following labeling with TAMRA-FP, samples were analyzed by SDS–PAGE. Coomassie staining (left) shows total protein; in-gel fluorescence (right) reports on TAMRA-FP labeling.


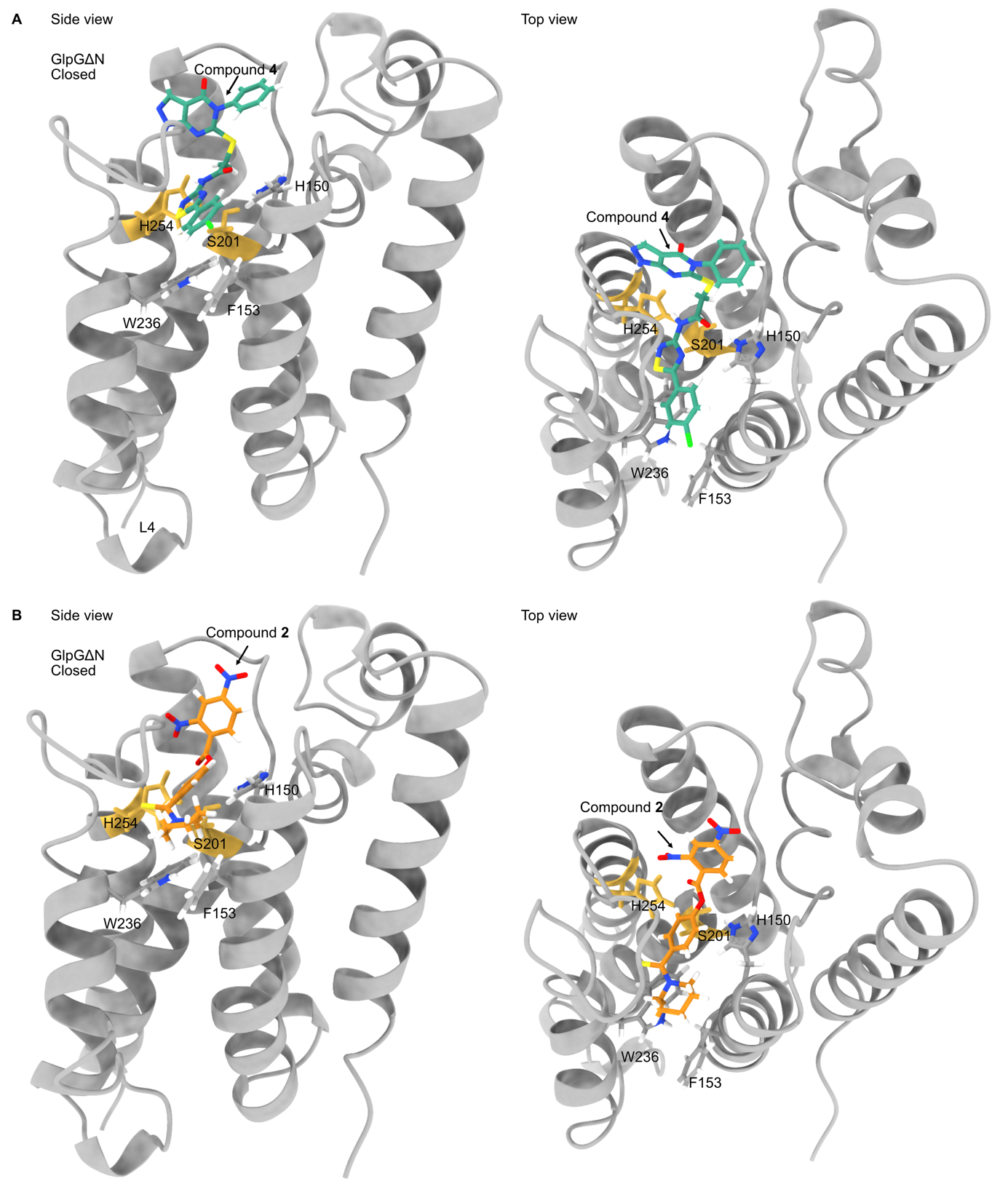


**Figure S11.** Docking of compounds **2** and **4** to GlpGΔN in the closed state (PDB ID: 2NRF). **(A)** Left: side view of GlpG (grey) in cartoon representation with compound **4** (green) in stick representation. The active site (S201 on TM4 and H254 on TM6, respectively) is shown in yellow. Right: Top view of GlpG with compound **4**. Important residues are shown in stick representation (H150, F153, W236). **(B)** Left: side view of GlpG (grey) with compound **2** (orange) in cartoon representation. The active site (S201 on TM4 and H254 on TM6, respectively) is shown in yellow. Right: Top view of GlpG with compound **2**. Important residues are shown in stick representation (H150, F153, W236)


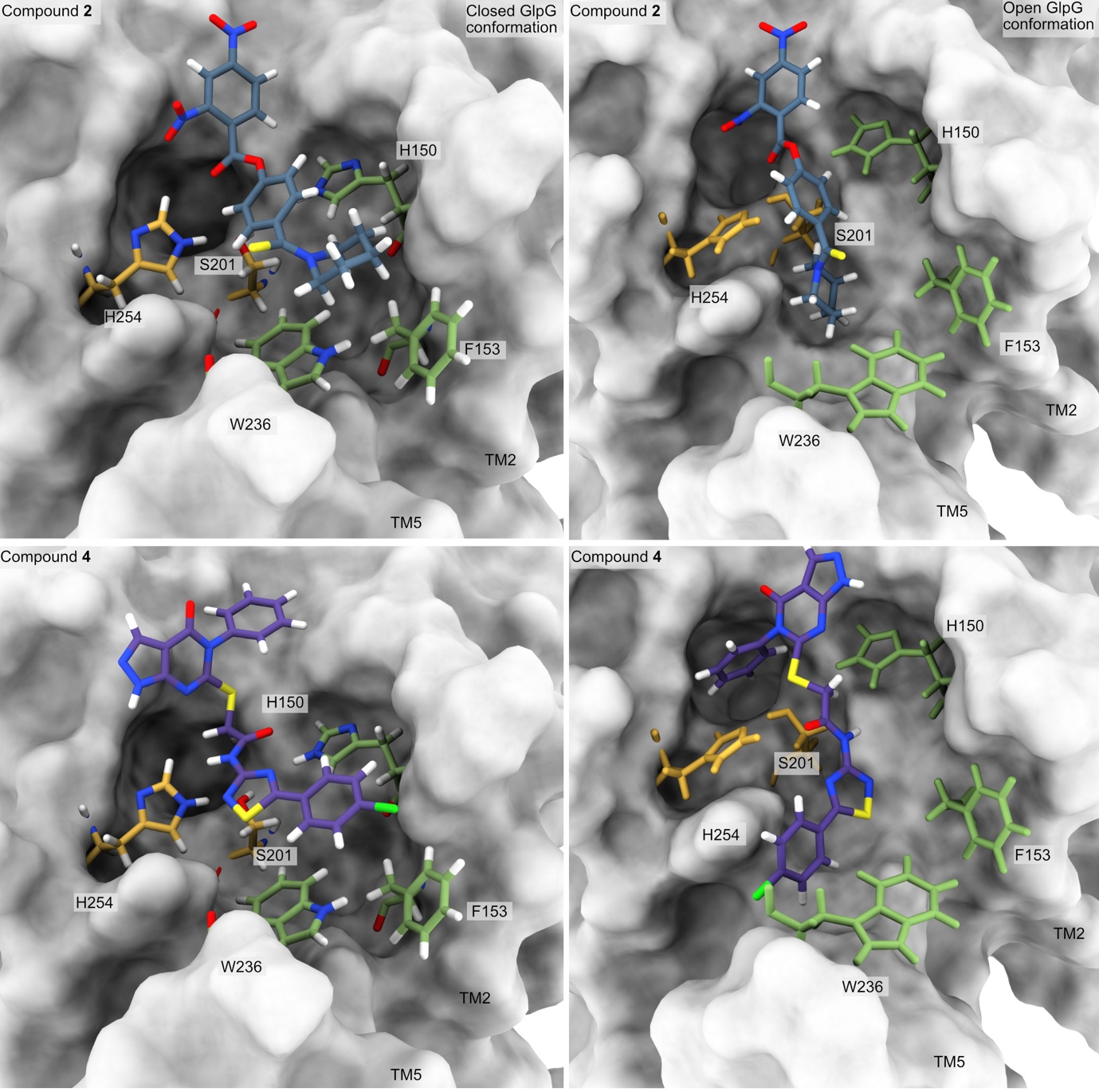


**Figure S12.** Docking poses of compounds **2** and **4** in the closed structure of GlpG (left) and in the open structure (right) with loop L5 removed for better visibility (grey surface; PDB ID: 2NRF). Compounds are shown in colored stick representation, while the active site residues are highlighted in yellow, and key gating (TM2,TM5) residues (F153 and W236) and H150 are shown in green sticks.


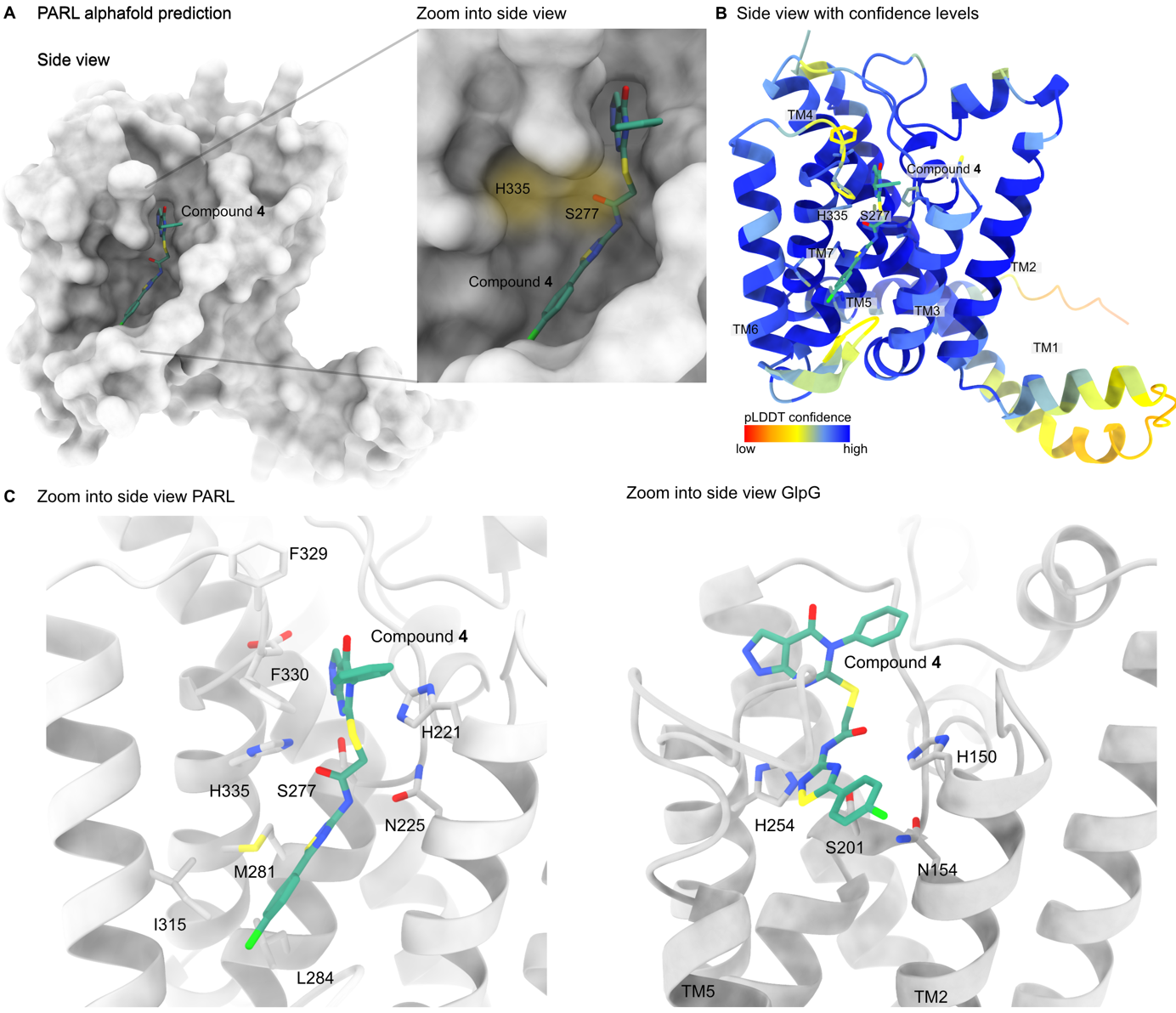


**Figure S13.** Docking pose of compound **4** in the AlphaFold v3.0 predicted structure of PARL. **(A)** Surface representation of PARL (AA 78-379) in side view is shown in grey, zoomed-in view of the active site is shown on the right. Compound **4** is shown in green, with predicted locations of the active site serine and histidine (S277, H335) marked in yellow. **(B)** Cartoon representation of PARL in side view with confidence levels and important residues in stick representation. AlphaFold provides a per-residue model confidence score (pLDDT) ranging from 0 to 100 shown through colouration (blue = high, to yellow = low), with very high confidence defined as pLDDT > 90 and high confidence as 70 < pLDDT < 90. **(C)** A zoomed-in view of the active site of PARL is shown on the right and of GlpG on the left for comparison. Besides the compound (green) other important residues (blue) are highlighted.

Based on the AlphaFold prediction, we assume the following secondary structure elements: TM1 extends from I99 to L140, TM2 is formed by D164 to L182, TM3 consists of L219 to L240, TM4 extends from Q242 to T267, while TM5 spans S277 to K290, TM6 is defined from A308 to I324, and TM7 from H332 to W354.


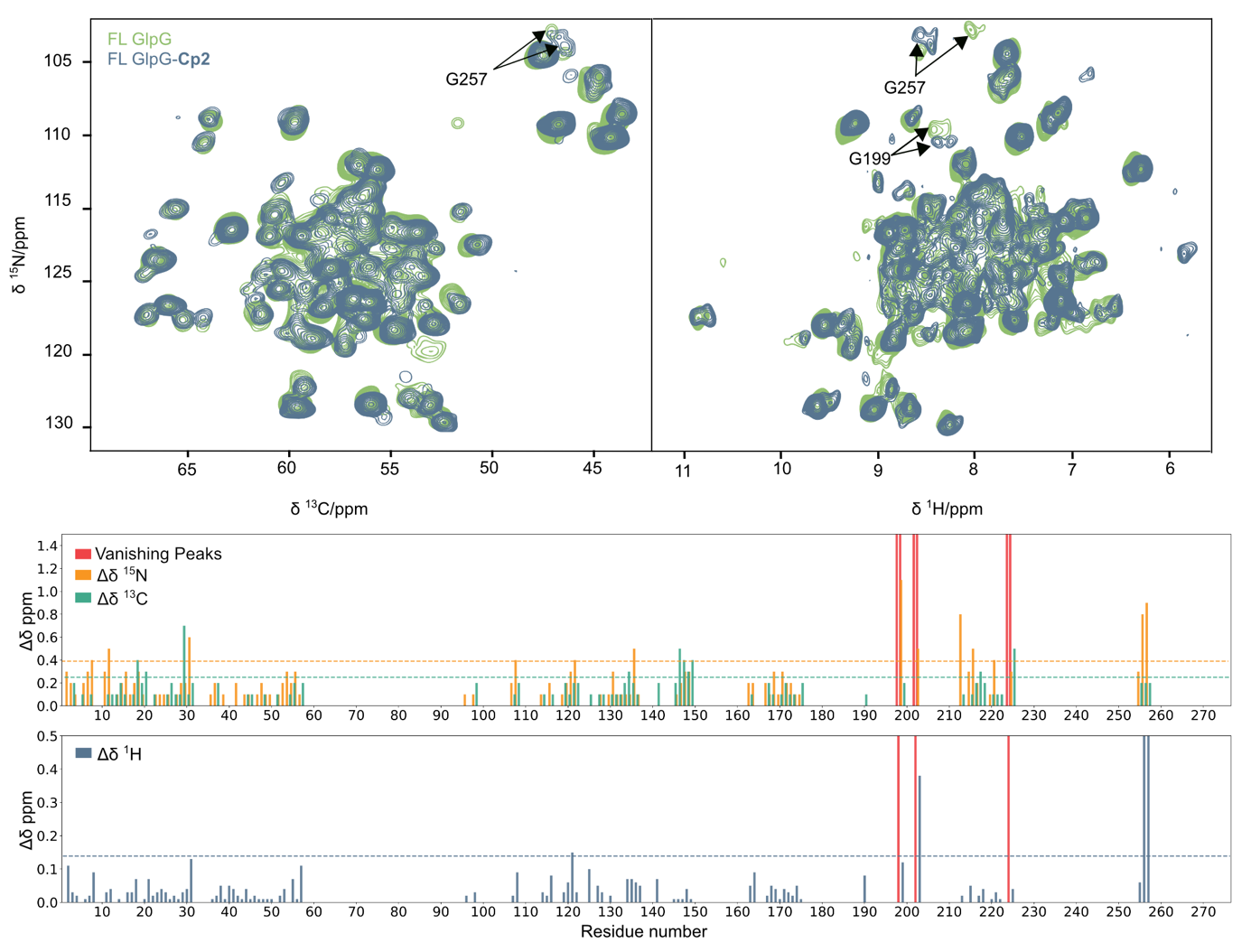


**Figure S14.** hCANH and HN projections for FL GlpG bound to compound **2 (top)**. Absolute chemical shift difference bar plots for the ^15^N, ^13^Cα, and ^1^H dimensions **(bottom)**. Values are derived from Table S4 & Table S8. Dashed lines indicate the statistical threshold used to classify significant chemical shift changes (mean + 1 SD).

**Supplementary Tables**

**Table S1.** *In vitro* fluorescent assay values for different compounds active on GlpG (FL and ΔN) and PARL, and inactive on α-chymotrypsin, are presented alongside the 2D structures of the compounds. Compounds with IDs starting with "5" are undisclosed, while those starting with "7" are natural products (AnalytiCon Discovery). IC50 = half-maximal inhibitory concentration, RD = response difference, and HC = Hill coefficient. (marked with star, compound **1** / 100940, **2** / 205640, **3** / 207927, **4** / 208274, **5** / 300541, and **6** / 710980).

**Table S2.** Similar compounds to compounds **2** and **4**, with internal ID, PubChem ID, 2D structure and similarity score.

| Internal ID | PubChem ID | Structure | Similarity Score |
| --- | --- | --- | --- |
| Compound **2** | 3112714 | 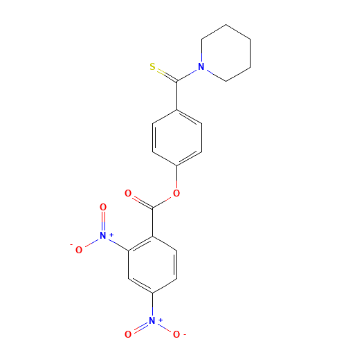 | 1 |
| S21 | 3112748 | 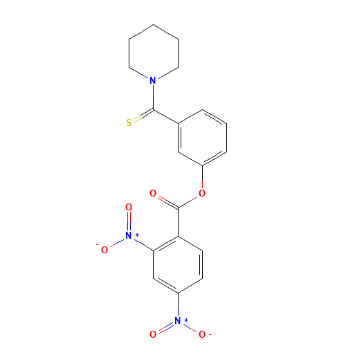 | 0.9211 |
| S22 | 1334764 | 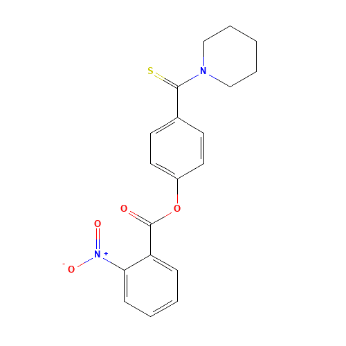 | 0.8919 |
| S23 | 1127187 | 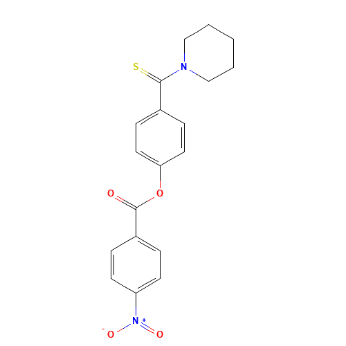 | 0.8889 |
| S24 | 3949320 | 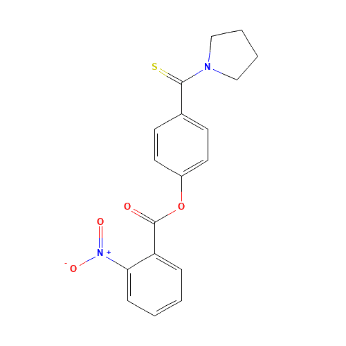 | 0.8649 |
| S25 | 3112716 | 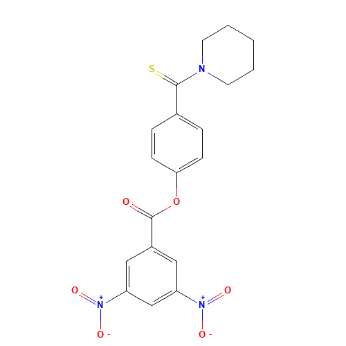 | 0.8649 |
| S26 | 3112731 | 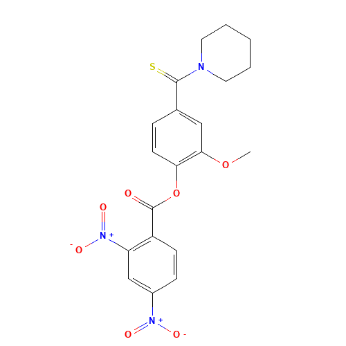 | 0.8500 |
| S27 | 3112754 | 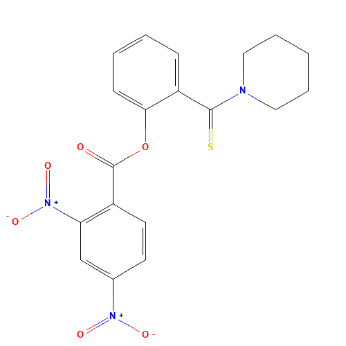 | 0.8462 |
| S28 | 1127238 | 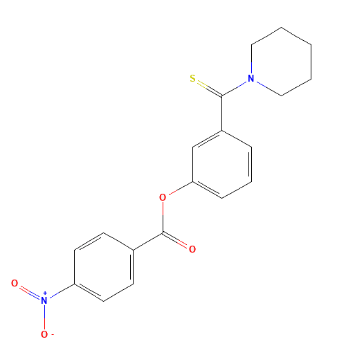 | 0.8421 |
| S29 | 1127186 | 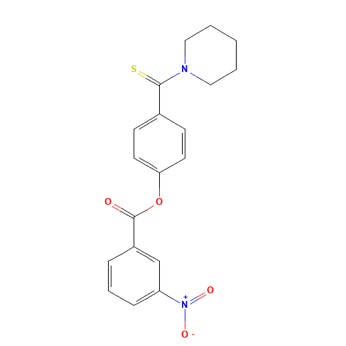 | 0.8421 |
| S30 | 3112736 | 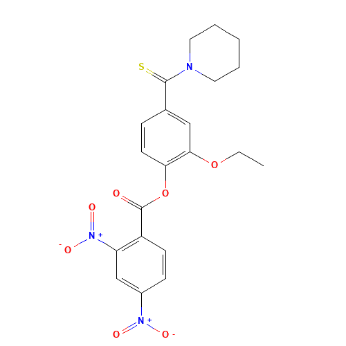 | 0.8293 |

| Internal ID | PubChem ID | Structure | Similarity Score |
| --- | --- | --- | --- |
| Compound **4** | 2243105 | 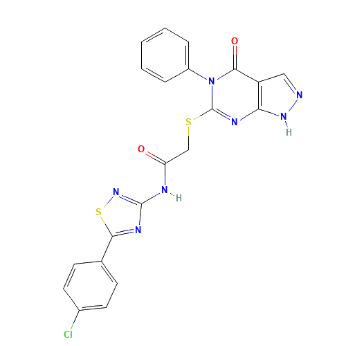 | 1 |
| S41 | 2200352 | 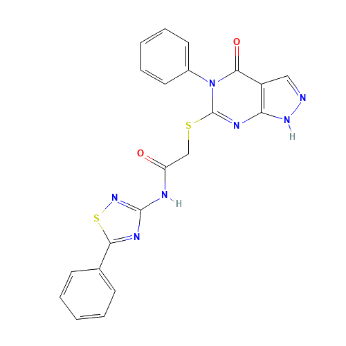 | 0.9 |
| S42 | 2200622 | 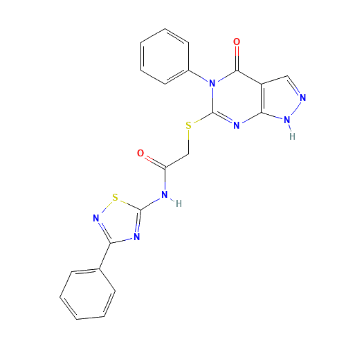 | 0.9 |
| S43 | 15998846 | 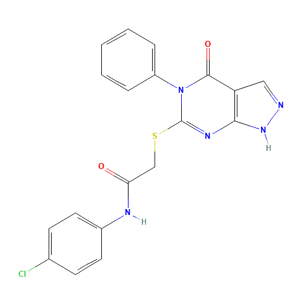 | 0.7037 |
| S44 | 3484961 | 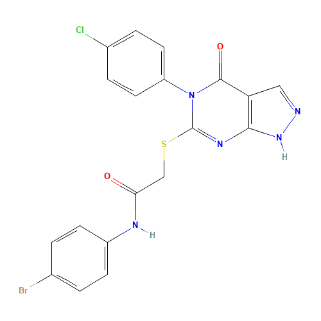 | 0.7037 |
| S45 | 1284152 | 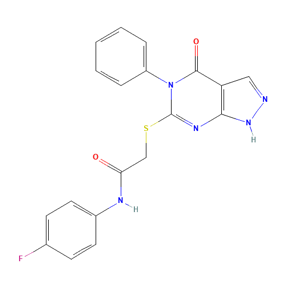 | 0.7037 |
| S46 | 2241291 | 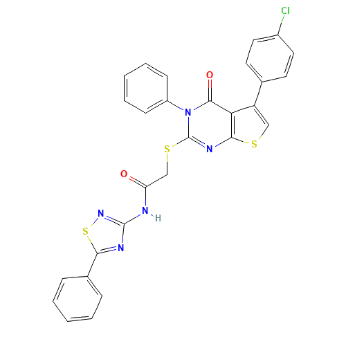 | 0.7018 |
| S47 | 2243062 | 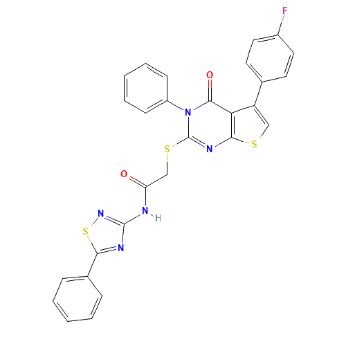 | 0.7018 |
| S48 | 2243059 | 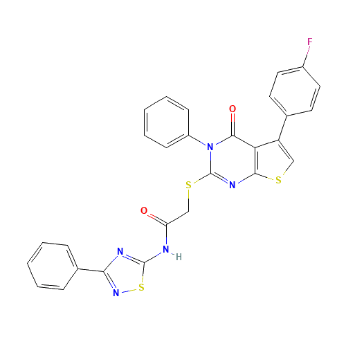 | 0.7018 |
| S49 | 4354207 | 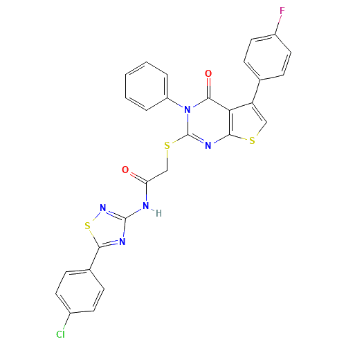 | 0.7018 |

**Table S3.** *In vitro* fluorescent assay values for different compounds active on PARL, and inactive on chymotrypsin, are presented alongside the 2D structures of the compounds. Compounds with IDs starting with "5" are undisclosed, while those starting with "7" are natural products (AnalytiCon Discovery). IC50 = half-maximal inhibitory concentration, RD = response difference, and HC = Hill coefficient.

**Table S4.** Chemical shifts of identified peaks based on hCANH spectrum of Apo FL GlpG, Reference to compounds **2** & **4**.

| **Residue** | **H** | **N** | **CA** | **Residue** | **H** | **N** | **CA** |
| --- | --- | --- | --- | --- | --- | --- | --- |
| 2 LEU | 8.85 | 121.9 | 53.6 | 114 LEU | 8.83 | 116.1 | 55.1 |
| 3 MET | 8.72 | 128.9 | 55.7 | 115 GLY | 7.2 | 108.8 | 44.2 |
| 4 ILE | 8.89 | 127 | 59.5 | 116 ASP | 8.05 | 119.9 | 57.8 |
| 5 THR | 7.27 | 109.2 | 59.8 | 119 VAL | 6.92 | 115 | 65.7 |
| 6 SER | 7.72 | 115.8 | 56.5 | 120 MET | 8.73 | 120.4 | 59.4 |
| 7 PHE | 8.95 | 121.4 | 56.3 | 121 LEU | 7.95 | 115.4 | 57.8 |
| 8 ALA | 8.93 | 124.8 | 53.2 | 122 TRP | 7.34 | 117.2 | 58.3 |
| 11 ARG | 8.12 | 118.1 | 58.1 | 125 TRP | 8.05 | 119.2 | 55.2 |
| 12 VAL | 7.31 | 122.8 | 65.2 | 127 PHE | 5.78 | 117.9 | 53.9 |
| 13 ALA | 6.83 | 118.9 | 54.2 | 128 ASP | 7.09 | 121.3 | 52 |
| 14 GLN | 7.85 | 114.6 | 57.1 | 130 THR | 8.66 | 108.9 | 63.8 |
| 15 ALA | 7.65 | 121 | 54.7 | 131 LEU | 7.76 | 119.8 | 53.7 |
| 16 PHE | 7.5 | 118.9 | 61.1 | 132 LYS | 6.91 | 115.7 | 58.1 |
| 17 VAL | 8.07 | 119.3 | 66.9 | 133 PHE | 8.52 | 115.2 | 56.4 |
| 18 ASP | 9.36 | 123 | 57.2 | 134 GLU | 7.23 | 119.9 | 54.8 |
| 19 TYR | 8.32 | 120.6 | 62.2 | 135 PHE | 7.57 | 116.8 | 59.5 |
| 20 MET | 8.58 | 119.5 | 56 | 136 TRP | 6.89 | 115.8 | 60.2 |
| 21 ALA | 8.12 | 123.2 | 55.1 | 137 ARG | 7.03 | 122 | 57.1 |
| 22 THR | 7.53 | 110.6 | 64 | 141 HIS | 7.1 | 121.1 | 56.8 |
| 23 GLN | 7.17 | 117.8 | 53 | 145 HIS | 6.34 | 112.2 | 56 |
| 24 GLY | 7.67 | 106.2 | 45.3 | 146 PHE | 10.62 | 118.6 | 59.7 |
| 25 VAL | 7.75 | 120.5 | 60.6 | 147 SER | 8.14 | 112.1 | 57 |
| 26 ILE | 9.08 | 128.5 | 59.8 | 148 LEU | 9.85 | 123.8 | 57 |
| 27 LEU | 8.28 | 129.4 | 52.7 | 149 MET | 8.85 | 116.7 | 57.8 |
| 28 THR | 9.01 | 113.1 | 60.4 | 163 GLY | 6.8 | 106.4 | 46.8 |
| 29 ILE | 8.83 | 123.2 | 59.7 | 164 ALA | 6.65 | 121.8 | 54.7 |
| 30 GLN | 9.57 | 128 | 54.3 | 167 LYS | 8.33 | 117.1 | 57.7 |
| 31 GLN | 8.99 | 127 | 54.7 | 168 ARG | 7.9 | 113.8 | 56.2 |
| 36 ASP | 8.93 | 127.5 | 53.5 | 169 LEU | 8.87 | 116.4 | 53.8 |
| 37 VAL | 8.68 | 122.3 | 61.5 | 170 GLY | 7.56 | 110.2 | 44.7 |
| 38 TRP | 9.62 | 128.3 | 56.2 | 171 SER | 8.8 | 116.3 | 62.9 |
| 39 LEU | 9.57 | 122.7 | 53.1 | 172 GLY | 9.3 | 109.5 | 47.3 |
| 40 ALA | 8.71 | 128.5 | 53.4 | 173 LYS | 8.11 | 121.5 | 56.3 |
| 41 ASP | 7.36 | 115.2 | 51.8 | 174 LEU | 7.08 | 116 | 57.2 |
| 42 GLU | 8.89 | 124 | 58.9 | 175 ILE | 8.67 | 121.5 | 66 |
| 43 SER | 8.46 | 116.9 | 61 | 190 GLN | 7.68 | 116.4 | 56.4 |
| 44 GLN | 7.85 | 118.7 | 54.2 | 198 GLY | 8.56 | 104.3 | 46.7 |
| 45 ALA | 7.63 | 122.5 | 56.5 | 199 GLY | 8.34 | 109.5 | 45 |
| 46 GLU | 8.75 | 116.9 | 59.5 | 202 GLY | 8.84 | 112.8 | 47.6 |
| 47 ARG | 8.15 | 121.8 | 58.4 | 203 VAL | 8.01 | 123.1 | 64.2 |
| 48 VAL | 8.48 | 118.5 | 66.6 | 213 LEU | 8.29 | 117.2 | 57.2 |
| 49 ARG | 8.77 | 119.2 | 60.4 | 214 ARG | 8.73 | 119.1 | 58.1 |
| 50 ALA | 8.2 | 123.3 | 54.9 | 215 GLY | 7.67 | 104.5 | 47.9 |
| 51 GLU | 8.48 | 118.2 | 57.9 | 216 GLU | 7.3 | 117.9 | 57.4 |
| 52 LEU | 9.31 | 124.1 | 57.4 | 217 ARG | 8.08 | 114.2 | 56.5 |
| 53 ALA | 7.6 | 119.3 | 54.9 | 218 ASP | 8.38 | 117.5 | 51.2 |
| 54 ARG | 7.3 | 116.8 | 58.6 | 220 GLN | 8.84 | 117.8 | 57.3 |
| 55 PHE | 8.55 | 123.6 | 60 | 221 SER | 8.13 | 115.3 | 60.7 |
| 56 LEU | 8.51 | 115 | 56.2 | 222 GLY | 7.79 | 106.7 | 45.4 |
| 57 GLU | 7.35 | 116.9 | 58.4 | 224 TYR | 7.59 | 118.9 | 56.7 |
| 96 VAL | 10.88 | 122.3 | 66.9 | 225 LEU | 9 | 124.7 | 53.1 |
| 98 TRP | 8.38 | 119.5 | 61.2 | 255 ILE | 7.83 | 116.9 | 63.8 |
| 107 VAL | 8.19 | 118.4 | 66.3 | 256 ALA | 7.97 | 121.4 | 55 |
| 108 PHE | 8.77 | 119.4 | 56.4 | 257 GLY | 8.01 | 102.7 | 47.3 |

**Table S5.** Chemical shifts (ppm) of identified peaks based on hCANH and HCONH spectra of FL GlpG with compound **4**.

| **Residue** | **H** | **N** | **CA** | **C** | **Residue** | **H** | **N** | **CA** | **C** |
| --- | --- | --- | --- | --- | --- | --- | --- | --- | --- |
| 2 LEU | 8.83 | 121.9 | 53.5 | 175.1 | 119 VAL | 6.92 | 115.1 | 65.6 | - |
| 3 MET | 8.73 | 128.9 | 55.5 | 175.8 | 120 MET | 8.79 | 120.6 | 59.4 | 177.8 |
| 4 ILE | 8.89 | 126.8 | 59.6 | 175.3 | 121 LEU | 7.97 | 115.8 | 58 | 179.6 |
| 5 THR | 7.25 | 109.2 | 59.9 | 170.3 | 122 TRP | 7.45 | 117.3 | 58.6 | - |
| 6 SER | 7.75 | 115.8 | 56.6 | 173.3 | 124 ALA | - | - | - | 176.8 |
| 7 PHE | 8.92 | 121.8 | 56.3 | 175 | 125 TRP | 8.18 | 119.2 | 54.7 | - |
| 8 ALA | 8.98 | 125.4 | 53.3 | - | 126 PRO | - | - | - | 177.7 |
| 10 PRO | - | - | - | 179.4 | 127 PHE | 5.78 | 118.1 | 54 | 173.3 |
| 11 ARG | 8.2 | 118.5 | 58.3 | 179.6 | 128 ASP | 7.11 | 121.7 | 51.9 | - |
| 12 VAL | 7.23 | 122.8 | 65.1 | 176.8 | 129 PRO | - | - | - | 178.5 |
| 13 ALA | 6.78 | 118.7 | 54.3 | 178.6 | 130 THR | 8.68 | 108.8 | 63.9 | 176.4 |
| 14 GLN | 7.86 | 114.3 | 57.2 | 176.8 | 131 LEU | 7.77 | 119.8 | 53.7 | 177.8 |
| 15 ALA | 7.63 | 121.2 | 54.6 | - | 132 LYS | 6.92 | 115.7 | 58.2 | 176.5 |
| 16 PHE | 7.54 | 118.8 | 61.2 | 177.1 | 133 PHE | 8.54 | 115.6 | 56.3 | 175.1 |
| 17 VAL | 8.09 | 119.5 | 67 | 179.2 | 134 GLU | 7.2 | 120.1 | 54.8 | 177.2 |
| 18 ASP | 9.33 | 123 | 57.2 | 179.4 | 135 PHE | 7.57 | 116.6 | 60 | - |
| 19 TYR | 8.32 | 120.3 | 61.8 | 179.6 | 136 TRP | 6.82 | 116.1 | 60.3 | 176.6 |
| 20 MET | 8.58 | 119.5 | 56.1 | 179.2 | 137 ARG | 7.02 | 122 | 57.1 | - |
| 21 ALA | 8.13 | 123.2 | 55 | - | 140 THR | - | - | - | 175.1 |
| 22 THR | 7.53 | 110.6 | 64 | 175.2 | 141 HIS | 7.14 | 121.2 | 56.9 | - |
| 23 GLN | 7.24 | 117.8 | 53 | 175.8 | 144 MET | - | - | - | 171.3 |
| 24 GLY | 7.69 | 106.2 | 45.3 | 173.7 | 145 HIS | 6.29 | 112.2 | 55.9 | 176.1 |
| 25 VAL | 7.75 | 120.5 | 60.5 | 174.3 | 146 PHE | 11.05 | 118.3 | 60.2 | 175.5 |
| 26 ILE | 9.09 | 128.6 | 59.8 | 175.2 | 147 SER | 8.31 | 112.8 | 56.8 | 174 |
| 27 LEU | 8.28 | 129.5 | 52.6 | 174.9 | 148 LEU | 9.96 | 123.7 | 57.3 | 178.2 |
| 28 THR | 8.97 | 112.7 | 60.4 | 173.4 | 149 MET | 9.11 | 116.6 | 58.2 | - |
| 29 ILE | 8.83 | 122.8 | 59.7 | 176.8 | 164 ALA | 6.67 | 121.6 | 54.9 | - |
| 30 GLN | 9.53 | 128 | 54.4 | - | 166 GLU | - | - | - | 179.2 |
| 31 GLN | 9.01 | 126.6 | 55 | - | 167 LYS | 8.31 | 117.2 | 57.9 | 178.3 |
| 35 SER | - | - | - | 172.3 | 168 ARG | 7.88 | 113.7 | 56.2 | 177.5 |
| 36 ASP | 8.92 | 127.3 | 53.8 | 175.1 | 169 LEU | 8.92 | 116.7 | 53.9 | 177.7 |
| 37 VAL | 8.7 | 122.3 | 61.4 | 174.1 | 170 GLY | 7.58 | 110.1 | 44.8 | 173.9 |
| 38 TRP | 9.64 | 128.2 | 56.2 | 174.3 | 171 SER | 8.79 | 116.5 | 62.9 | 176 |
| 39 LEU | 9.57 | 122.6 | 53.1 | 175.2 | 172 GLY | 9.31 | 109.6 | 47.3 | 174.9 |
| 40 ALA | 8.71 | 128.5 | 53.4 | 177.2 | 173 LYS | 8.15 | 121.4 | 56.3 | 177.9 |
| 41 ASP | 7.36 | 115.1 | 51.8 | 176.1 | 174 LEU | 7.13 | 115.9 | 57.3 | 180 |
| 42 GLU | 8.87 | 124.1 | 58.9 | 178.2 | 175 ILE | 8.65 | 121.5 | 66 | - |
| 43 SER | 8.5 | 116.9 | 61 | 176.3 | 189 GLN | - | - | - | 177.8 |
| 44 GLN | 7.85 | 118.8 | 54.3 | 176 | 190 GLN | 7.71 | 116.6 | 56.7 | - |
| 45 ALA | 7.65 | 122.6 | 56.5 | 178.6 | 198 GLY | - | - | - | 173.2 |
| 46 GLU | 8.74 | 116.9 | 59.4 | 179.5 | 199 GLY | 7.97 | 109.8 | 44.4 | - |
| 47 ARG | 8.17 | 121.8 | 58.6 | 179.2 | 201 SER | - | - | - | 177.2 |
| 48 VAL | 8.51 | 118.5 | 66.6 | 177.9 | 202 GLY | 8.78 | 112.3 | 47.1 | 175.3 |
| 49 ARG | 8.81 | 119.2 | 60.5 | 179.3 | 203 VAL | 7.89 | 124 | 64.6 | - |
| 50 ALA | 8.19 | 123.3 | 54.8 | - | 212 TRP | - | - | - | 178.2 |
| 51 GLU | 8.47 | 118.3 | 57.9 | 180.5 | 213 LEU | 8.16 | 117.3 | 57.2 | 178.5 |
| 52 LEU | 9.32 | 124.3 | 57.5 | 177.1 | 214 ARG | 8.72 | 119.1 | 58.1 | 177.9 |
| 53 ALA | 7.63 | 119.4 | 54.9 | 180.3 | 215 GLY | 7.7 | 104.7 | 48 | 176.5 |
| 54 ARG | 7.28 | 116.7 | 58.6 | 178.3 | 216 GLU | 7.28 | 117.7 | 57.8 | 178.9 |
| 55 PHE | 8.53 | 123.5 | 59.9 | 176.4 | 217 ARG | 8.03 | 114.3 | 56.7 | 176.3 |
| 56 LEU | 8.53 | 115.3 | 56.3 | 179.9 | 218 ASP | 8.4 | 117.4 | 51.1 | - |
| 57 GLU | 7.39 | 117 | 58.4 | - | 219 PRO | - | - | - | 179.8 |
| 96 VAL | 10.94 | 122.5 | 66.9 | - | 220 GLN | 8.83 | 117.9 | 57.2 | 177.1 |
| 97 THR | - | - | - | 179.6 | 221 SER | 8.23 | 115.7 | 60.9 | 176 |
| 98 TRP | 8.39 | 119.4 | 61.1 | - | 222 GLY | 7.76 | 106.5 | 45.5 | - |
| 107 VAL | 8.18 | 118.1 | 66.3 | 177.9 | 223 ILE | - | - | - | 173.6 |
| 108 PHE | 8.69 | 119.2 | 56.2 | - | 224 TYR | 7.91 | 119.7 | 57.7 | 173.6 |
| 113 ILE | - | - | - | 177.2 | 225 LEU | 8.94 | 125.1 | 53.1 | - |
| 114 LEU | 8.86 | 116.5 | 55.2 | 178.1 | 255 ILE | 7.85 | 117.2 | 63.8 | 176.7 |
| 115 GLY | 7.21 | 108.9 | 44.2 | 174.1 | 256 ALA | 8.6 | 120.8 | 55 | 179 |
| 116 ASP | 8.11 | 120.5 | 57.8 | - | 257 GLY | 8.81 | 104.2 | 47 | - |
| 118 GLU | - | - | - | 177.8 |  |  |  |  |  |

**Table S6.** Chemical shifts (ppm) of identified peaks based on hCANH spectrum of Apo FL GlpG, Reference to CMK.

| **Residue** | **H** | **N** | **CA** | **Residue** | **H** | **N** | **CA** |
| --- | --- | --- | --- | --- | --- | --- | --- |
| 2 LEU | 8.82 | 121.7 | 53.6 | 115 GLY | 7.16 | 108.7 | 44.2 |
| 3 MET | 8.72 | 129 | 55.6 | 116 ASP | 8.04 | 120.1 | 57.7 |
| 4 ILE | 8.89 | 127.2 | 59.4 | 119 VAL | 6.92 | 114.9 | 65.7 |
| 5 THR | 7.24 | 109.3 | 59.9 | 120 MET | 8.72 | 120.5 | 59.2 |
| 6 SER | 7.73 | 115.7 | 56.5 | 121 LEU | 7.93 | 115.7 | 57.9 |
| 7 PHE | 8.96 | 121.7 | 56.4 | 122 TRP | 7.36 | 117.2 | 58.2 |
| 8 ALA | 8.95 | 124.8 | 53 | 125 TRP | 8.12 | 119.3 | 55.2 |
| 9 ASN | 8.23 | 117.2 | 49.3 | 127 PHE | 5.76 | 118.1 | 53.9 |
| 11 ARG | 8.16 | 118.3 | 58.3 | 128 ASP | 7.07 | 121.4 | 52 |
| 12 VAL | 7.22 | 122.8 | 65 | 130 THR | 8.66 | 108.9 | 64 |
| 13 ALA | 6.77 | 118.7 | 54.2 | 131 LEU | 7.76 | 119.8 | 53.6 |
| 14 GLN | 7.91 | 114.4 | 57 | 132 LYS | 6.88 | 115.7 | 58.1 |
| 15 ALA | 7.64 | 121.4 | 54.9 | 133 PHE | 8.54 | 115.4 | 56.4 |
| 16 PHE | 7.51 | 119.2 | 61.2 | 134 GLU | 7.21 | 120 | 54.8 |
| 17 VAL | 8.07 | 119.4 | 67 | 135 PHE | 7.54 | 116.8 | 59.7 |
| 18 ASP | 9.39 | 122.8 | 56.8 | 136 TRP | 6.83 | 115.9 | 60.3 |
| 19 TYR | 8.32 | 120.7 | 62.1 | 137 ARG | 7.02 | 122 | 57.1 |
| 20 MET | 8.59 | 119.3 | 56.1 | 141 HIS | 7.1 | 121.2 | 56.8 |
| 21 ALA | 8.12 | 123.1 | 55.1 | 145 HIS | 6.33 | 112.3 | 56 |
| 22 THR | 7.52 | 110.6 | 64.1 | 146 PHE | 10.52 | 118.4 | 59.5 |
| 23 GLN | 7.11 | 117.8 | 53 | 147 SER | 8.11 | 112 | 57 |
| 24 GLY | 7.61 | 106.2 | 45.2 | 148 LEU | 9.91 | 124 | 56.9 |
| 25 VAL | 7.72 | 120.5 | 60.6 | 149 MET | 8.95 | 116.2 | 58.4 |
| 26 ILE | 9.04 | 128.6 | 59.8 | 163 GLY | 6.95 | 106.4 | 47.1 |
| 27 LEU | 8.27 | 129.4 | 52.7 | 164 ALA | 6.59 | 121.7 | 54.7 |
| 28 THR | 9.01 | 112.9 | 60.4 | 167 LYS | 8.31 | 116.9 | 57.7 |
| 29 ILE | 8.79 | 123.1 | 59.8 | 168 ARG | 7.88 | 113.9 | 56.3 |
| 30 GLN | 9.55 | 127.9 | 54.3 | 169 LEU | 8.86 | 116.5 | 53.9 |
| 31 GLN | 9.03 | 126.7 | 54.8 | 170 GLY | 7.54 | 110.1 | 44.7 |
| 36 ASP | 8.92 | 127.5 | 53.7 | 171 SER | 8.77 | 116.5 | 62.9 |
| 37 VAL | 8.66 | 122.5 | 61.6 | 172 GLY | 9.35 | 109.6 | 47.2 |
| 38 TRP | 9.62 | 128.2 | 56.2 | 173 LYS | 8.11 | 121.5 | 56.3 |
| 39 LEU | 9.56 | 122.8 | 53.1 | 174 LEU | 7.03 | 115.8 | 57.4 |
| 40 ALA | 8.7 | 128.4 | 53.3 | 175 ILE | 8.67 | 121.6 | 65.9 |
| 41 ASP | 7.36 | 115.2 | 51.8 | 190 GLN | 7.66 | 116.4 | 56.5 |
| 42 GLU | 8.85 | 124.2 | 58.9 | 194 GLY | 7.95 | 111.9 | 44.9 |
| 43 SER | 8.46 | 116.9 | 61 | 198 GLY | 8.54 | 104.2 | 46.7 |
| 44 GLN | 7.81 | 118.7 | 54.2 | 199 GLY | 8.32 | 109.5 | 45.1 |
| 45 ALA | 7.61 | 122.5 | 56.5 | 201 SER | 8.22 | 115.6 | 64 |
| 46 GLU | 8.74 | 116.9 | 59.5 | 202 GLY | 8.88 | 112.5 | 47.6 |
| 47 ARG | 8.12 | 121.8 | 58.5 | 203 VAL | 7.8 | 122.9 | 64.3 |
| 48 VAL | 8.47 | 118.5 | 66.6 | 213 LEU | 8.32 | 117.2 | 57.4 |
| 49 ARG | 8.8 | 119.2 | 60.5 | 214 ARG | 8.72 | 119.2 | 58.2 |
| 50 ALA | 8.21 | 123.4 | 54.9 | 215 GLY | 7.63 | 104.5 | 47.9 |
| 51 GLU | 8.44 | 118.2 | 57.9 | 216 GLU | 7.26 | 117.8 | 57.4 |
| 52 LEU | 9.3 | 124.2 | 57.4 | 217 ARG | 8.01 | 114.5 | 56.6 |
| 53 ALA | 7.58 | 119.4 | 54.9 | 218 ASP | 8.37 | 117.5 | 51.1 |
| 54 ARG | 7.26 | 116.7 | 58.6 | 220 GLN | 8.81 | 117.8 | 57.3 |
| 55 PHE | 8.49 | 123.6 | 60.1 | 221 SER | 8.12 | 115.5 | 60.8 |
| 56 LEU | 8.49 | 114.9 | 56.2 | 222 GLY | 7.77 | 106.5 | 45.4 |
| 57 GLU | 7.35 | 116.9 | 58.4 | 224 TYR | 7.64 | 118.7 | 56.8 |
| 96 VAL | 10.85 | 122.2 | 67 | 225 LEU | 8.97 | 124.7 | 52.9 |
| 98 TRP | 8.31 | 119.4 | 61.3 | 255 ILE | 7.82 | 117.1 | 63.8 |
| 107 VAL | 8.18 | 118.3 | 66.4 | 256 ALA | 7.9 | 121.1 | 54.9 |
| 108 PHE | 8.81 | 119.2 | 56.4 | 257 GLY | 7.98 | 102.7 | 47.2 |
| 114 LEU | 8.82 | 116.3 | 55.1 |  |  |  |  |

**Table S7.** Chemical shifts (ppm) of identified peaks based on hCANH, hCONH, hCAcoNH, and hCOcaNH spectra of FL GlpG with CMK.

| **Residue** | **H** | **N** | **CA** | **C** | **Residue** | **H** | **N** | **CA** | **C** |
| --- | --- | --- | --- | --- | --- | --- | --- | --- | --- |
| 2 LEU | 8.87 | 121.6 | 53.6 | 174.8 | 120 MET | 8.89 | 120.8 | 59.7 | 177.7 |
| 3 MET | 8.74 | 129 | 55.5 | 175.7 | 121 LEU | 8.12 | 115.7 | 57.9 | - |
| 4 ILE | 8.86 | 127 | 59.5 | 175.2 | 122 TRP | 7.43 | 117.2 | 58 | - |
| 5 THR | 7.26 | 109 | 59.8 | 170.3 | 126 PRO | - | - | - | 177.6 |
| 6 SER | 7.75 | 115.7 | 56.6 | 173 | 127 PHE | 5.88 | 118 | 54 | - |
| 7 PHE | 8.96 | 121.8 | 56.6 | 175 | 128 ASP | 7.12 | 121.6 | 51.9 | - |
| 8 ALA | 8.94 | 125.1 | 53.3 | - | 129 PRO | - | - | - | 178.4 |
| 9 ASN | 8.2 | 117.1 | 49.5 | - | 130 THR | 8.67 | 108.8 | 64 | 176.3 |
| 10 PRO | - | - | - | 179.4 | 131 LEU | 7.75 | 119.7 | 53.7 | 177.9 |
| 11 ARG | 8.12 | 118.7 | 58.2 | 179.5 | 132 LYS | 6.92 | 115.6 | 58.1 | 176.5 |
| 12 VAL | 7.26 | 122.5 | 65.2 | 176.8 | 133 PHE | 8.6 | 116.1 | 56.5 | 175.1 |
| 13 ALA | 6.79 | 118.7 | 54.2 | 178.7 | 134 GLU | 7.2 | 119.7 | 54.7 | 177.1 |
| 14 GLN | 7.82 | 114.4 | 57.2 | 176.6 | 135 PHE | 7.57 | 116.7 | 59.8 | 175.4 |
| 15 ALA | 7.62 | 121.3 | 54.8 | 179.9 | 136 TRP | 6.83 | 115.5 | 60.4 | - |
| 16 PHE | 7.53 | 118.7 | 61.3 | 177.2 | 137 ARG | 6.98 | 121.9 | 57.2 | - |
| 17 VAL | 8.06 | 119.4 | 67 | - | 140 THR | - | - | 65.9 | 175.3 |
| 18 ASP | 9.42 | 123.1 | 56.9 | - | 141 HIS | 7.21 | 121.4 | 57.2 | - |
| 19 TYR | 8.34 | 120.3 | 61.8 | 179.7 | 144 MET | - | - | 55.8 | 171.6 |
| 20 MET | 8.57 | 119.7 | 56.1 | 179.3 | 145 HIS | 6.3 | 112.5 | 55.8 | 175.5 |
| 21 ALA | 8.07 | 123.1 | 55.2 | 182.1 | 146 PHE | 10.42 | 118.9 | 59 | 175 |
| 22 THR | 7.53 | 110.3 | 64.2 | 175.1 | 147 SER | 8.2 | 112.7 | 56.7 | 174.1 |
| 23 GLN | 7.2 | 117.7 | 53 | 175.8 | 148 LEU | 10.03 | 123.8 | 57.2 | - |
| 24 GLY | 7.66 | 106.2 | 45.3 | 173.6 | 149 MET | 9.02 | 116.8 | 58.1 | - |
| 25 VAL | 7.74 | 120.4 | 60.7 | 174.3 | 163 GLY | 6.92 | 106.4 | 46.8 | - |
| 26 ILE | 9.04 | 128.5 | 59.8 | 175.1 | 164 ALA | 6.69 | 121.9 | 54.8 | - |
| 27 LEU | 8.29 | 129.4 | 52.7 | 175.1 | 166 GLU | - | - | 59 | 179.2 |
| 28 THR | 9.02 | 113.1 | 60.4 | 173.5 | 167 LYS | 8.31 | 116.5 | 58 | 178.3 |
| 29 ILE | 8.81 | 123.1 | 59.7 | 176.7 | 168 ARG | 7.92 | 114 | 56.3 | 177.5 |
| 30 GLN | 9.5 | 127.8 | 54.4 | - | 169 LEU | 8.9 | 116.7 | 54 | 177.6 |
| 31 GLN | 9.12 | 126.2 | 54.4 | - | 170 GLY | 7.56 | 110.1 | 44.8 | 174 |
| 35 SER | - | - | - | 172.6 | 171 SER | 8.83 | 116.7 | 63 | 176 |
| 36 ASP | 8.9 | 127.5 | 53.5 | 175.1 | 172 GLY | 9.39 | 109.7 | 47.3 | 175 |
| 37 VAL | 8.66 | 122.1 | 61.5 | 174.1 | 173 LYS | 8.19 | 121.6 | 56.4 | 177.8 |
| 38 TRP | 9.65 | 128.2 | 56.3 | 174.4 | 174 LEU | 7.17 | 116 | 57.3 | 180.1 |
| 39 LEU | 9.58 | 122.8 | 53.2 | 175.2 | 175 ILE | 8.73 | 121.6 | 66 | - |
| 40 ALA | 8.71 | 128.4 | 53.3 | 177.1 | 189 GLN | - | - | 58.8 | - |
| 41 ASP | 7.37 | 115.1 | 51.8 | 176.1 | 190 GLN | 7.82 | 116.8 | 56.4 | - |
| 42 GLU | 8.86 | 124 | 58.9 | 178.1 | 194 GLY | 8.07 | 111.8 | 44.7 | - |
| 43 SER | 8.45 | 116.9 | 61 | 176.2 | 197 PHE | - | - | 55.4 | 173.1 |
| 44 GLN | 7.84 | 118.7 | 54.4 | 176.1 | 198 GLY | 8.28 | 101.5 | 46.4 | 172.6 |
| 45 ALA | 7.63 | 122.5 | 56.5 | 178.6 | 199 GLY | 8.38 | 112.7 | 43.5 | 176 |
| 46 GLU | 8.74 | 117 | 59.5 | 179.5 | 200 LEU | 10.29 | 126.1 | 54.5 | 177.7 |
| 47 ARG | 8.16 | 121.8 | 58.6 | 179.1 | 201 SER | 7.11 | 112.8 | 59.4 | - |
| 48 VAL | 8.48 | 118.4 | 66.5 | 177.9 | 202 GLY | - | - | 47.5 | - |
| 49 ARG | 8.77 | 119.2 | 60.6 | 179.2 | 203 VAL | 7.91 | 122.5 | 64.2 | - |
| 50 ALA | 8.19 | 123.2 | 54.9 | 181.4 | 212 TRP | - | - | 60.8 | 178.3 |
| 51 GLU | 8.43 | 118.2 | 58 | 180.6 | 213 LEU | 8.28 | 118.1 | 57.1 | 178.7 |
| 52 LEU | 9.31 | 124 | 57.4 | 177.2 | 214 ARG | 8.75 | 119.1 | 58.4 | 178 |
| 53 ALA | 7.64 | 119.5 | 54.9 | 180.4 | 215 GLY | 7.77 | 105.1 | 48 | 176.5 |
| 54 ARG | 7.22 | 116.6 | 58.8 | 178.5 | 216 GLU | 7.31 | 117.4 | 58 | 178.9 |
| 55 PHE | 8.5 | 123.4 | 60 | - | 217 ARG | 7.98 | 114.5 | 56.7 | 176.1 |
| 56 LEU | 8.49 | 114.6 | 56.1 | 179.7 | 218 ASP | 8.45 | 117.3 | 51.1 | - |
| 57 GLU | 7.45 | 117 | 57.9 | - | 219 PRO | - | - | 64.5 | 179.6 |
| 95 PRO | - | - | 65.5 | 179 | 220 GLN | 8.77 | 117.7 | 57.2 | 176.9 |
| 96 VAL | 10.9 | 122 | 67 | - | 221 SER | 8.17 | 115.7 | 61 | 176.1 |
| 97 THR | - | - | 67.3 | - | 222 GLY | 7.76 | 106.7 | 45.6 | - |
| 98 TRP | 8.38 | 119.5 | 61 | - | 223 ILE | - | - | 58.6 | 173.7 |
| 107 VAL | 8.16 | 117.9 | 66.3 | - | 224 TYR | 7.88 | 119.3 | 57.4 | - |
| 108 PHE | 8.77 | 119.2 | 56.3 | - | 225 LEU | 8.91 | 125 | 53.4 | - |
| 113 ILE | - | - | 63.4 | 177.3 | 252 GLY | 8.28 | 107.5 | 47 | 174 |
| 114 LEU | 8.85 | 116.5 | 55.2 | 178.1 | 253 ALA | 8.14 | 123.3 | 53.7 | 180.2 |
| 115 GLY | 7.21 | 108.6 | 44.2 | 174.1 | 254 HIS | 7.55 | 116 | 57.9 | 177.5 |
| 116 ASP | 8.11 | 120.6 | 57.7 | - | 255 ILE | 7.81 | 116.7 | 63.5 | 177 |
| 118 GLU | - | - | 59 | 178.1 | 256 ALA | 8.58 | 120.9 | 54.9 | - |
| 119 VAL | 7.09 | 114.7 | 66 | 178.7 | 257 GLY | 8.45 | 104.1 | 47.4 | - |

**Table S8.** Chemical shifts (ppm) of identified peaks based on hCANH and hCONH spectra of FL GlpG with compound **2**.

| **Residue** | **H** | **N** | **CA** | **C** | **Residue** | **H** | **N** | **CA** | **C** |
| --- | --- | --- | --- | --- | --- | --- | --- | --- | --- |
| 2 LEU | 8.74 | 121.6 | 53.6 | 175 | 116 ASP | 8.13 | 120.1 | 57.7 | - |
| 3 MET | 8.75 | 129.1 | 55.5 | 175.7 | 118 GLU | - | - | - | 177.7 |
| 4 ILE | 8.91 | 126.9 | 59.5 | 175.2 | 119 VAL | 6.95 | 115.1 | 65.5 | 178.7 |
| 5 THR | 7.27 | 109.2 | 59.9 | - | 120 MET | 8.79 | 120.6 | 59.3 | 177.8 |
| 6 SER | 7.71 | 116 | 56.5 | 172.8 | 121 LEU | 8.1 | 115.7 | 58 | 179.4 |
| 7 PHE | 8.97 | 121.7 | 56.2 | 175.2 | 122 TRP | 7.31 | 116.8 | 58.5 | - |
| 8 ALA | 9.02 | 125.2 | 53.2 | - | 125 TRP | 8.15 | 119.2 | 55.1 | - |
| 10 PRO | - | - | - | 179.4 | 126 PRO | - | - | - | 177.7 |
| 11 ARG | 8.15 | 118.4 | 58.2 | 179.6 | 127 PHE | 5.83 | 117.9 | 54 | 173.3 |
| 12 VAL | 7.27 | 122.3 | 65.1 | 176.9 | 128 ASP | 7.06 | 121.4 | 51.9 | - |
| 13 ALA | 6.83 | 118.9 | 54.1 | 178.6 | 129 PRO | - | - | - | 178.5 |
| 14 GLN | 7.86 | 114.5 | 56.9 | 176.8 | 130 THR | 8.68 | 109 | 63.9 | 176.2 |
| 15 ALA | 7.65 | 121.2 | 54.6 | - | 131 LEU | 7.76 | 119.5 | 53.6 | 177.7 |
| 16 PHE | 7.53 | 118.6 | 61.1 | 177.2 | 132 LYS | 6.91 | 115.6 | 58 | 176.7 |
| 17 VAL | 8.04 | 119.4 | 67 | 179.3 | 133 PHE | 8.52 | 115.3 | 56.6 | 174.9 |
| 18 ASP | 9.43 | 122.8 | 56.8 | 179.4 | 134 GLU | 7.16 | 119.8 | 54.5 | 177 |
| 19 TYR | 8.32 | 120.3 | 62 | 179.5 | 135 PHE | 7.5 | 116.9 | 59.3 | 175.4 |
| 20 MET | 8.59 | 119.6 | 55.7 | 179.4 | 136 TRP | 6.83 | 115.3 | 60.3 | 176.7 |
| 21 ALA | 8.05 | 123.2 | 55.1 | - | 137 ARG | 7.08 | 121.9 | 57.1 | - |
| 22 THR | 7.51 | 110.6 | 64.1 | 175.2 | 140 THR | - | - | - | 175.3 |
| 23 GLN | 7.2 | 117.7 | 53 | 176 | 141 HIS | 7.17 | 121.1 | 57 | - |
| 24 GLY | 7.71 | 106.3 | 45.3 | 173.6 | 144 MET | - | - | - | 171.7 |
| 25 VAL | 7.78 | 120.4 | 60.5 | 174.3 | 145 HIS | 6.33 | 112.2 | 55.8 | 175.6 |
| 26 ILE | 9.07 | 128.6 | 59.6 | 175.3 | 146 PHE | 10.63 | 118.5 | 59.2 | 175.2 |
| 27 LEU | 8.3 | 129.4 | 52.6 | 175.1 | 147 SER | 8.15 | 111.9 | 56.6 | 173.9 |
| 28 THR | 9.02 | 113 | 60.3 | 173.5 | 148 LEU | 9.81 | 123.5 | 56.7 | - |
| 29 ILE | 8.86 | 123.4 | 59 | 176.8 | 149 MET | 8.86 | 116.4 | 58.2 | - |
| 30 GLN | 9.53 | 127.8 | 54.2 | - | 163 GLY | 6.85 | 106.2 | 46.7 | - |
| 31 GLN | 9.12 | 126.4 | 54.5 | - | 164 ALA | 6.56 | 121.6 | 54.7 | - |
| 35 SER | - | - | - | 172.9 | 166 GLU | - | - | - | 179.1 |
| 36 ASP | 8.92 | 127.4 | 53.5 | 174.9 | 167 LYS | 8.31 | 116.9 | 57.5 | 178.6 |
| 37 VAL | 8.66 | 122.1 | 61.3 | 174.2 | 168 ARG | 7.95 | 113.9 | 56.3 | 177.6 |
| 38 TRP | 9.67 | 128.3 | 56.2 | 174.5 | 169 LEU | 8.91 | 116.7 | 53.8 | 177.7 |
| 39 LEU | 9.58 | 122.8 | 53.1 | 175.3 | 170 GLY | 7.57 | 110.1 | 44.6 | 174 |
| 40 ALA | 8.76 | 128.5 | 53.4 | 177.1 | 171 SER | 8.76 | 116.6 | 62.7 | 176 |
| 41 ASP | 7.4 | 115.2 | 51.8 | 176.1 | 172 GLY | 9.27 | 109.3 | 47.2 | 174.9 |
| 42 GLU | 8.87 | 123.8 | 58.9 | 178.1 | 173 LYS | 8.13 | 121.3 | 56.2 | 177.8 |
| 43 SER | 8.45 | 116.9 | 61 | 176.3 | 174 LEU | 7.13 | 115.9 | 57.2 | 180 |
| 44 GLN | 7.89 | 118.8 | 54.3 | 176.1 | 175 ILE | 8.68 | 121.6 | 65.8 | - |
| 45 ALA | 7.62 | 122.4 | 56.4 | 178.6 | 189 GLN | - | - | - | 178.1 |
| 46 GLU | 8.73 | 116.9 | 59.5 | 179.5 | 190 GLN | 7.76 | 116.4 | 56.5 | - |
| 47 ARG | 8.14 | 121.7 | 58.4 | 179.1 | 198 GLY | - | - | - | 173.7 |
| 48 VAL | 8.47 | 118.3 | 66.5 | 177.8 | 199 GLY | 8.46 | 110.6 | 44.8 | - |
| 49 ARG | 8.78 | 119.3 | 60.4 | 179.2 | 203 VAL | 7.63 | 122.6 | 64.2 | - |
| 50 ALA | 8.19 | 123.2 | 54.9 | 181.3 | 212 TRP | - | - | - | 177.9 |
| 51 GLU | 8.48 | 118.2 | 58 | 180.4 | 213 LEU | 8.27 | 118 | 57.3 | 178.8 |
| 52 LEU | 9.33 | 124.2 | 57.4 | 177.2 | 214 ARG | 8.73 | 119.1 | 58.1 | 178 |
| 53 ALA | 7.64 | 119.5 | 54.9 | 180.4 | 215 GLY | 7.72 | 104.8 | 47.8 | 176.3 |
| 54 ARG | 7.3 | 116.5 | 58.5 | - | 216 GLU | 7.3 | 117.4 | 57.6 | 178.7 |
| 55 PHE | 8.48 | 123.4 | 60.2 | 176.5 | 217 ARG | 8.1 | 114.4 | 56.8 | 176.3 |
| 56 LEU | 8.52 | 115.3 | 56.2 | 179.4 | 218 ASP | 8.42 | 117.5 | 51 | - |
| 57 GLU | 7.46 | 116.8 | 58.6 | - | 219 PRO | - | - | - | 179.5 |
| 95 PRO | - | - | - | 178.9 | 220 GLN | 8.83 | 117.7 | 57.2 | 177.4 |
| 96 VAL | 10.86 | 122.2 | 66.9 | - | 221 SER | 8.16 | 115.7 | 60.8 | 176 |
| 97 THR | - | - | - | 179.5 | 222 GLY | 7.8 | 106.7 | 45.3 | - |
| 98 TRP | 8.35 | 119.4 | 61 | - | 224 TYR | - | - | - | 173.3 |
| 107 VAL | 8.17 | 118.6 | 66.2 | 178 | 225 LEU | 8.96 | 125 | 53.6 | - |
| 108 PHE | 8.86 | 119 | 56.2 | - | 255 ILE | 7.77 | 117.2 | 63.6 | 176.7 |
| 113 ILE | - | - | - | 177.3 | 256 ALA | 8.55 | 120.6 | 54.8 | 179.1 |
| 114 LEU | 8.86 | 116.2 | 55.2 | 178.2 | 257 GLY | 8.63 | 103.6 | 47.1 | - |
| 115 GLY | 7.18 | 108.8 | 44.2 | 174.2 |  |  |  |  |  |

**Table S9.** Summary of chemical shift differences (Δδ) for compounds **2**, **4**, and CMK, categorized as strong (Δδ > mean + 3× standard deviation), moderate (Δδ > mean + 2× standard deviation), and weak (Δδ > mean + standard deviation) for each dimension ^15^N, ^13^Cα, and ^1^H (N_diff, CA_diff and H_diff). Cp = Compound, Stdev = standard deviation. (These classifications help to identify regions of significant perturbation in the molecular environment upon binding or structural modifications).

| Cp **4** | **Mean** | **Stdev** |  |  | CMK | **Mean** | **Stdev** |  |  | Cp **2** | **Mean** | **Stdev** |  |  |
| --- | --- | --- | --- | --- | --- | --- | --- | --- | --- | --- | --- | --- | --- | --- |
| H_diff | 0.0565 | 0.1154 | Mean + **3× Stdev** | 0.4026 | H_diff | 0.0695 | 0.1333 | Mean + **3× Stdev** | 0.4693 | H_diff | 0.0489 | 0.0898 | Mean + **3× Stdev** | 0.3183 |
|  |  |  | Mean + **2× Stdev** | 0.2873 |  |  |  | Mean + **2× Stdev** | 0.3360 |  |  |  | Mean + **2× Stdev** | 0.2285 |
|  |  |  | Mean + **1× Stdev** | 0.1719 |  |  |  | Mean + **1× Stdev** | 0.2027 |  |  |  | Mean + **1× Stdev** | 0.1387 |
| N_diff | 0.1896 | 0.2251 | Mean + **3× Stdev** | 0.8648 | N_diff | 0.2780 | 0.4950 | Mean + **3× Stdev** | 1.7631 | N_diff | 0.1933 | 0.1962 | Mean + **3× Stdev** | 0.7821 |
|  |  |  | Mean + **2× Stdev** | 0.6397 |  |  |  | Mean + **2× Stdev** | 1.2681 |  |  |  | Mean + **2× Stdev** | 0.5858 |
|  |  |  | Mean + **1× Stdev** | 0.4147 |  |  |  | Mean + **1× Stdev** | 0.7730 |  |  |  | Mean + **1× Stdev** | 0.3896 |
| CA_diff | 0.1245 | 0.1608 | Mean + **3× Stdev** | 0.607 | CA_diff | 0.1954 | 0.4685 | Mean + **3× Stdev** | 1.6010 | CA_diff | 0.1257 | 0.1241 | Mean + **3× Stdev** | 0.4979 |
|  |  |  | Mean + **2× Stdev** | 0.4462 |  |  |  | Mean + **2× Stdev** | 1.1325 |  |  |  | Mean + **2× Stdev** | 0.3738 |
|  |  |  | Mean + **1× Stdev** | 0.2854 |  |  |  | Mean + **1× Stdev** | 0.6639 |  |  |  | Mean + **1× Stdev** | 0.2498 |

References:

[1] C. Shi, C. Öster, C. Bohg, L. Li, S. Lange, V. Chevelkov, A. Lange, “Structure and Dynamics of the Rhomboid Protease GlpG in Liposomes Studied by Solid-State NMR” *J. Am. Chem. Soc.* **2019**, *141*, 17314–17321.

[2] P. Fricke, V. Chevelkov, M. Zinke, K. Giller, S. Becker, A. Lange, “Backbone assignment of perdeuterated proteins by solid-state NMR using proton detection and ultrafast magic-angle spinning” *Nat Protoc* **2017**, *12*, 764–782.

[3] C. Bohg, C. Öster, T. Utesch, S. Bischoff, S. Lange, C. Shi, H. Sun, A. Lange, “A combination of solid-state NMR and MD simulations reveals the binding mode of a rhomboid protease inhibitor” *Chem. Sci.* **2021**, *12*, 12754–12762.

[4] L. G. Stevenson, K. Strisovsky, K. M. Clemmer, S. Bhatt, M. Freeman, P. N. Rather, “Rhomboid protease AarA mediates quorum-sensing in Providencia stuartii by activating TatA of the twin-arginine translocase” *PNAS* **2007**, *104*, 1003–1008.

[5] R. P. Baker, S. Urban in *Methods in Enzymology*, Elsevier, **2017**, pp. 229–253.

[6] E. Arutyunova, L. Lysyk, M. Morrison, C. Brooks, M. Joanne Lemieux, “Expression and Purification of Human Mitochondrial Intramembrane Protease PARL” *Methods Mol Biol* **2021**, *2302*, 1–20.

[7] L. Lysyk, R. Brassard, N. Touret, M. J. Lemieux, “PARL Protease: A Glimpse at Intramembrane Proteolysis in the Inner Mitochondrial Membrane” *Journal of Molecular Biology* **2020**, *432*, 5052–5062.

[8] L. Lysyk, R. Brassard, E. Arutyunova, V. Siebert, Z. Jiang, E. Takyi, M. Morrison, H. S. Young, M. K. Lemberg, A. J. O’Donoghue, M. J. Lemieux, “Insights into the catalytic properties of the mitochondrial rhomboid protease PARL” *Journal of Biological Chemistry* **2021**, *296*, 100383.

[9] A. Tichá, S. Stanchev, K. R. Vinothkumar, D. C. Mikles, P. Pachl, J. Began, J. Škerle, K. Švehlová, M. T. N. Nguyen, S. H. L. Verhelst, D. C. Johnson, D. A. Bachovchin, M. Lepšík, P. Majer, K. Strisovsky, “General and Modular Strategy for Designing Potent, Selective, and Pharmacologically Compliant Inhibitors of Rhomboid Proteases” *Cell Chemical Biology* **2017**, *24*, 1523-1536.e4.

[10] S. Zoll, S. Stanchev, J. Began, J. Škerle, M. Lepšík, L. Peclinovská, P. Majer, K. Strisovsky, “Substrate binding and specificity of rhomboid intramembrane protease revealed by substrate–peptide complex structures” *EMBO J* **2014**, *33*, 2408–2421.

[11] M. Lisurek, B. Rupp, J. Wichard, M. Neuenschwander, J. P. von Kries, R. Frank, J. Rademann, R. Kühne, “Design of chemical libraries with potentially bioactive molecules applying a maximum common substructure concept” *Mol Divers* **2010**, *14*, 401–408.

[12] K. Bach, J. Dohnálek, J. Škerlová, J. Kuzmík, E. Poláchová, S. Stanchev, P. Majer, J. Fanfrlík, A. Pecina, J. Řezáč, M. Lepšík, V. Borshchevskiy, V. Polovinkin, K. Strisovsky, “Extensive targeting of chemical space at the prime side of ketoamide inhibitors of rhomboid proteases by branched substituents empowers their selectivity and potency” *European Journal of Medicinal Chemistry* **2024**, *275*, 116606.

[13] C. Brideau, B. Gunter, B. Pikounis, A. Liaw, “Improved statistical methods for hit selection in high-throughput screening” *J Biomol Screen* **2003**, *8*, 634–647.

[14] J. H. Zhang, T. D. Chung, K. R. Oldenburg, “A Simple Statistical Parameter for Use in Evaluation and Validation of High Throughput Screening Assays” *J Biomol Screen* **1999**, *4*, 67–73.

[15] C. Ritz, F. Baty, J. C. Streibig, D. Gerhard, “Dose-Response Analysis Using R” *PLOS ONE* **2015**, *10*, e0146021.

[16] H. J. Motulsky, R. E. Brown, “Detecting outliers when fitting data with nonlinear regression – a new method based on robust nonlinear regression and the false discovery rate” *BMC Bioinformatics* **2006**, *7*, 123.

[17] M. R. Berthold, N. Cebron, F. Dill, T. R. Gabriel, T. Kötter, T. Meinl, P. Ohl, K. Thiel, B. Wiswedel, “KNIME - the Konstanz information miner: version 2.0 and beyond” *SIGKDD Explor. Newsl.* **2009**, *11*, 26–31.

[18] R Core, “R: A language and environment for statistical computing. R Foundation for Statistical Computing, Vienna, Austria” *Online: https://www. r-project. org* **2022**.

[19] E. Poláchová, K. Bach, E. Heuten, S. Stanchev, A. Tichá, P. Lampe, P. Majer, T. Langer, M. K. Lemberg, K. Stříšovský, “Chemical Blockage of the Mitochondrial Rhomboid Protease PARL by Novel Ketoamide Inhibitors Reveals Its Role in PINK1/Parkin-Dependent Mitophagy” *J Med Chem* **2022**, *66*, 251–265.

[20] M. P. Jacobson, D. L. Pincus, C. S. Rapp, T. J. F. Day, B. Honig, D. E. Shaw, R. A. Friesner, “A hierarchical approach to all-atom protein loop prediction” *Proteins: Structure, Function, and Bioinformatics* **2004**, *55*, 351–367.

[21] R. A. Friesner, R. B. Murphy, M. P. Repasky, L. L. Frye, J. R. Greenwood, T. A. Halgren, P. C. Sanschagrin, D. T. Mainz, “Extra Precision Glide: Docking and Scoring Incorporating a Model of Hydrophobic Enclosure for Protein−Ligand Complexes” *Journal of Medicinal Chemistry* **2006**, *49*, 6177–6196.

[22] M. Varadi, D. Bertoni, P. Magana, U. Paramval, I. Pidruchna, M. Radhakrishnan, M. Tsenkov, S. Nair, M. Mirdita, J. Yeo, O. Kovalevskiy, K. Tunyasuvunakool, A. Laydon, A. Žídek, H. Tomlinson, D. Hariharan, J. Abrahamson, T. Green, J. Jumper, E. Birney, M. Steinegger, D. Hassabis, S. Velankar, “AlphaFold Protein Structure Database in 2024: providing structure coverage for over 214 million protein sequences” *Nucleic Acids Research* **2024**, *52*, D368–D375.

[23] J. Abramson, J. Adler, J. Dunger, R. Evans, T. Green, A. Pritzel, O. Ronneberger, L. Willmore, A. J. Ballard, J. Bambrick, S. W. Bodenstein, D. A. Evans, C.-C. Hung, M. O’Neill, D. Reiman, K. Tunyasuvunakool, Z. Wu, A. Žemgulytė, E. Arvaniti, C. Beattie, O. Bertolli, A. Bridgland, A. Cherepanov, M. Congreve, A. I. Cowen-Rivers, A. Cowie, M. Figurnov, F. B. Fuchs, H. Gladman, R. Jain, Y. A. Khan, C. M. R. Low, K. Perlin, A. Potapenko, P. Savy, S. Singh, A. Stecula, A. Thillaisundaram, C. Tong, S. Yakneen, E. D. Zhong, M. Zielinski, A. Žídek, V. Bapst, P. Kohli, M. Jaderberg, D. Hassabis, J. M. Jumper, “Accurate structure prediction of biomolecular interactions with AlphaFold 3” *Nature* **2024**, *630*, 493–500.

[24] T. D. Goddard, C. C. Huang, E. C. Meng, E. F. Pettersen, G. S. Couch, J. H. Morris, T. E. Ferrin, “UCSF ChimeraX: Meeting modern challenges in visualization and analysis” *Protein Sci* **2018**, *27*, 14–25.

[25] E. F. Pettersen, T. D. Goddard, C. C. Huang, E. C. Meng, G. S. Couch, T. I. Croll, J. H. Morris, T. E. Ferrin, “UCSF ChimeraX: Structure visualization for researchers, educators, and developers” *Protein Sci* **2021**, *30*, 70–82.

[26] E. C. Meng, T. D. Goddard, E. F. Pettersen, G. S. Couch, Z. J. Pearson, J. H. Morris, T. E. Ferrin, “UCSF ChimeraX: Tools for structure building and analysis” *Protein Science* **2023**, *32*, e4792.

[27] Z. Wu, N. Yan, L. Feng, A. Oberstein, H. Yan, R. P. Baker, L. Gu, P. D. Jeffrey, S. Urban, Y. Shi, “Structural analysis of a rhomboid family intramembrane protease reveals a gating mechanism for substrate entry” *Nat. Struct. Mol. Biol.* **2006**, *13*, 1084–1091.

[28] G. Jones, P. Willett, R. C. Glen, A. R. Leach, R. Taylor, “Development and validation of a genetic algorithm for flexible docking” *J Mol Biol* **1997**, *267*, 727–748.

[29] O. Korb, T. Stützle, T. E. Exner, “Empirical scoring functions for advanced protein-ligand docking with PLANTS” *J Chem Inf Model* **2009**, *49*, 84–96.

[30] S. G. Hyberts, K. Takeuchi, G. Wagner, “Poisson-Gap Sampling and Forward Maximum Entropy Reconstruction for Enhancing the Resolution and Sensitivity of Protein NMR Data” *J. Am. Chem. Soc.* **2010**, *132*, 2145–2147.

[31] S. G. Hyberts, A. G. Milbradt, A. B. Wagner, H. Arthanari, G. Wagner, “Application of iterative soft thresholding for fast reconstruction of NMR data non-uniformly sampled with multidimensional Poisson Gap scheduling” *J Biomol NMR* **2012**, *52*, 315–327.

[32] V. Y. Orekhov, V. A. Jaravine, “Analysis of non-uniformly sampled spectra with multi-dimensional decomposition” *Progress in nuclear magnetic resonance spectroscopy* **2011**, *59*, 271–292.

[33] X. Qu, M. Mayzel, J.-F. Cai, Z. Chen, V. Orekhov, “Accelerated NMR Spectroscopy with Low-Rank Reconstruction” *Angewandte Chemie International Edition* **2015**, *54*, 852–854.

[34] K. Kazimierczuk, V. Yu. Orekhov, “Accelerated NMR Spectroscopy by Using Compressed Sensing” *Angewandte Chemie International Edition* **2011**, *50*, 5556–5559.

[35] F. Delaglio, S. Grzesiek, G. W. Vuister, G. Zhu, J. Pfeifer, A. Bax, “NMRPipe: a multidimensional spectral processing system based on UNIX pipes” *J. Biomol. NMR* **1995**, *6*, 277–293.

[36] E. Nimerovsky, A. C. Varkey, M. Kim, S. Becker, L. B. Andreas, “Simplified Preservation of Equivalent Pathways Spectroscopy” *JACS Au* **2023**, *3*, 2763–2771.

[37] S. P. Skinner, R. H. Fogh, W. Boucher, T. J. Ragan, L. G. Mureddu, G. W. Vuister, “CcpNmr AnalysisAssign: a flexible platform for integrated NMR analysis” *J Biomol NMR* **2016**, *66*, 111–124.

[38] D. Rogers, M. Hahn, “Extended-connectivity fingerprints” *J Chem Inf Model* **2010**, *50*, 742–754.

[39] K. R. Vinothkumar, K. Strisovsky, A. Andreeva, Y. Christova, S. Verhelst, M. Freeman, “The structural basis for catalysis and substrate specificity of a rhomboid protease” *The EMBO Journal* **2010**, *29*, 3797–3809.

[40] B. Cordier, M. K. Lemberg in *Methods in Enzymology*, Elsevier, **2017**, pp. 99–126.

[41] C. Bohg, C. Öster, B. Türkaydin, M. Lisurek, P. Sanchez-Carranza, S. Lange, T. Utesch, H. Sun, A. Lange, “The opening dynamics of the lateral gate regulates the activity of rhomboid proteases” *Science Advances* **2023**, *9*, eadh3858.
